# Supplementary material for: Regulation of membrane fluidity by RNF145‐triggered degradation of the lipid hydrolase ADIPOR2
Source: EMBO J. 2022 Aug 22;41(19):e110777. doi: 10.15252/embj.2022110777 (PMC9531299; doi:10.15252/embj.2022110777)
Supplement: Supplementary file 1 — Appendix S1 [file EMBJ-41-e110777-s005.pdf]

# Appendix Figures

# Appendix Materials and Methods

## Volkmar N. et al.,


| Contents | Page |
|---|---|
| **Appendix Figure S1.** Confirmation of knockdown efficiencies and expression of ADIPOR2 constructs. | **2** |
| **Appendix Figure S2.** RNF145/ADIPOR2 knockdown efficiencies in cells used for FRAP and SMT analysis. | **3** |
| **Appendix Materials and Methods:**<br><br>**Collection and processing of Mass spectrometry data.** Workflow (A) and data processing (B) of data represented in **Figure 1A & B** and **EV Fig 1A**. | **4-7** |

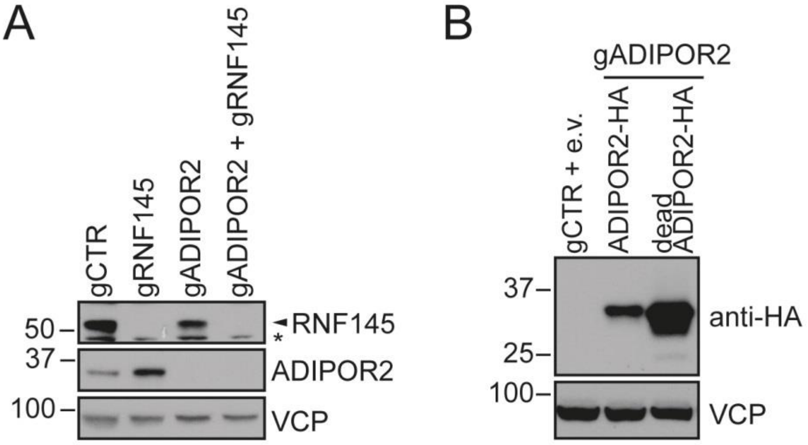


**Appendix Figure S1. Confirmation of knockdown efficiencies and expression of ADIPOR2 constructs. (A)** Cells (gCTR or gRNF145) in used for experiments in **Figure 5A** were analysed by immunoblotting before treatment with FAs. LE, long exposure; *non-specific bands. **(B)** Immunoblot analysis of cells used in **Figure 5C**. ADIPOR2-depleted cells were stably complemented with epitope-tagged WT ADIPOR2 (ADIPOR2-HA) or a catalytically inactive ADIPOR2 mutant (dead ADIPOR2-HA). Expression levels of recombinant ADIPOR2 variants before exposure to FAs were determined by immunoblot analysis using a specific anti-HA antibody.

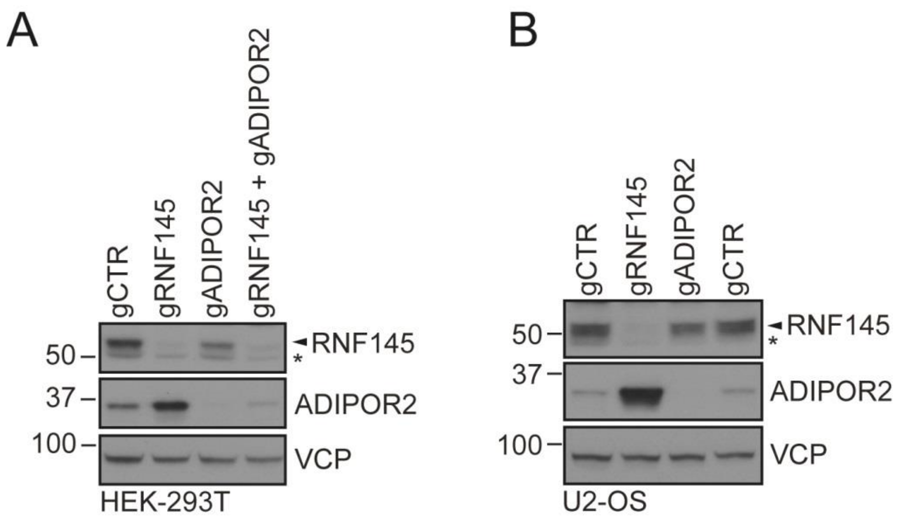


**Appendix Figure S2. RNF145/ADIPOR2 knockdown efficiencies in cells used for FRAP and SMT analysis. (A)** CRISPR/Cas9-mediated stable depletion of RNF145 (gRNF145), ADIPOR2 (gADIPOR2), or B2M (gCTR) in HEK-293T cells used in FRAP analysis (**Figure 6A**). **(B)** CRISPR/Cas9-mediated stable depletion of RNF145 (gRNF145), ADIPOR2 (gADIPOR2), or B2M (gCTR) in U2-OS cells used for SMT analysis (**Figure 6B**). Cell lines were generated by transfecting U2-OS cells with pools of 4 sgRNAs against the indicated targets. Knockdown cell pools were selected by puromycin for 72h.

# (A) MS Method - TMT

- Start Time (min): **0**
- End Time (min): **190**
- Cycle Time (sec): **3**

Master Scan:
- MS OT
  - Detector Type: **Orbitrap**
  - Orbitrap Resolution: **120000**
  - Mass Range: **Normal**
  - Use Quadrupole Isolation: **True**
  - Scan Range (m/z): **400-1500**
  - RF Lens (%): **60**
  - AGC Target: **Custom**
  - Normalized AGC Target (%): **125**
  - Maximum Injection Time Mode: **Custom**
  - Maximum Injection Time (ms): **50**
  - Microscans: **1**
  - Data Type: **Profile**
  - Polarity: **Positive**
  - Source Fragmentation: **Disabled**
  - Scan Description:

Filters:
- MIPS
  - Monoisotopic Peak Determination: **Peptide**
- Charge State
  - Include charge state(s): **2-7**
  - Include undetermined charge states: **False**
- Dynamic Exclusion
  - Use Common Settings: **False**
  - Exclude after n times: **1**
  - Exclusion duration (s): **90**
  - Mass Tolerance: **ppm**
  - Low: **10**
  - High: **10**
  - Exclude Isotopes: **True**
  - Perform dependent scan on single charge state per precursor only: **True**
- Intensity
  - Filter Type: **Intensity Threshold**
  - Intensity Threshold: **5.0e3**

- Data Dependent
  - Data Dependent Mode: **Cycle Time**
  - Time between Master Scans (sec): **3**

- Scan Event Type 1:
  - Scan:
  - ddMS$^2$ IT CID
    - Isolation Mode: **Quadrupole**
    - Isolation Window (m/z): **0.7**
    - Isolation Offset: **Off**
    - Activation Type: **CID**
    - Collision Energy Mode: **Fixed**
    - CID Collision Energy (%): **35**
    - CID Activation Time (ms): **10**
    - Activation Q: **0.25**
    - Multistage Activation: **False**
    - Detector Type: **Ion Trap**
    - Ion Trap Scan Rate: **Rapid**
    - Mass Range: **Normal**
    - Scan Range Mode: **Auto**
    - AGC Target: **Custom**
    - Normalized AGC Target (%): **80**
    - Maximum Injection Time Mode: **Auto**
    - Microscans: **1**
    - Data Type: **Centroid**
    - Scan Description:

Filters:
- Precursor Selection Range
  - Selection Range Mode: **Mass Range**
  - Mass Range (m/z): **400-2000**
- Precursor Ion Exclusion
  - Exclusion mass width: **m/z**
  - Low: **19**
  - High: **7**
- Isobaric Tag Loss Exclusion
  - Reagent: **TMT**

- Data Dependent
  - Data Dependent Mode: **Scans Per Outcome**

- Scan Event Type 1:
  - Scan:
  - ddMS$^3$ OT HCD
    - MS$^n$ Level: **3**
    - Synchronous Precursor Selection: **True**
    - Number of SPS Precursors: **10**
    - MS Isolation Window (m/z): **2**
    - MS2 Isolation Window (m/z): **2**
    - Isolation Offset: **Off**
    - Activation Type: **HCD**
    - HCD Collision Energy (%): **65**
    - Detector Type: **Orbitrap**
    - Orbitrap Resolution: **50000**
    - Mass Range: **Normal**
    - Scan Range Mode: **Define m/z range**
    - Scan Range (m/z): **100-1000**
    - AGC Target: **Custom**
    - Normalized AGC Target (%): **40**
    - Maximum Injection Time Mode: **Custom**
    - Maximum Injection Time (ms): **120**
    - Microscans: **1**
    - Data Type: **Profile**
    - Use EASY-IC™: **False**
    - Scan Description:
    - Number of Dependent Scans: **3**

# MS Method – AP-MS

Start Time (min): **0**
End Time (min): **87**
Cycle Time (sec): **2**

Master Scan:
- MS OT
  - Detector Type: **Orbitrap**
  - Orbitrap Resolution: **120000**
  - Mass Range: **Normal**
  - Use Quadrupole Isolation: **True**
  - Scan Range (m/z): **400-1600**
  - RF Lens (%): **60**
  - AGC Target: **Standard**
  - Maximum Injection Time Mode: **Custom**
  - Maximum Injection Time (ms): **50**
  - Microscans: **1**
  - Data Type: **Profile**
  - Polarity: **Positive**
  - Source Fragmentation: **Disabled**
  - Scan Description:

Filters:
- Intensity
  - Filter Type: **Intensity Threshold**
  - Intensity Threshold: **5.0e3**
- MIPS
  - Monoisotopic Peak Determination: **Peptide**
- Dynamic Exclusion
  - Use Common Settings: **False**
  - Exclude after n times: **1**
  - Exclusion duration (s): **20**
  - Mass Tolerance: **ppm**
  - Low: **5**
  - High: **5**
  - Exclude Isotopes: **True**
  - Perform dependent scan on single charge state per precursor only: **True**
- Charge State
  - Include charge state(s): **2-4**
  - Include undetermined charge states: **False**

- Data Dependent
  - Data Dependent Mode: **Cycle Time**
  - Time between Master Scans (sec): **2**

- Scan Event Type 1:
  - Scan:
  - $ddMS^2$ IT HCD
    - Isolation Mode: **Quadrupole**
    - Isolation Window (m/z): **1.6**
    - Isolation Offset: **Off**
    - Activation Type: **HCD**
    - Collision Energy Mode: **Fixed**
    - HCD Collision Energy (%): **34**
    - Detector Type: **Ion Trap**
    - Ion Trap Scan Rate: **Rapid**
    - Mass Range: **Normal**
    - Scan Range Mode: **Auto**
    - AGC Target: **Standard**
    - Maximum Injection Time Mode: **Auto**
    - Microscans: **1**
    - Data Type: **Centroid**
    - Scan Description:

# (B) Searching Schematic

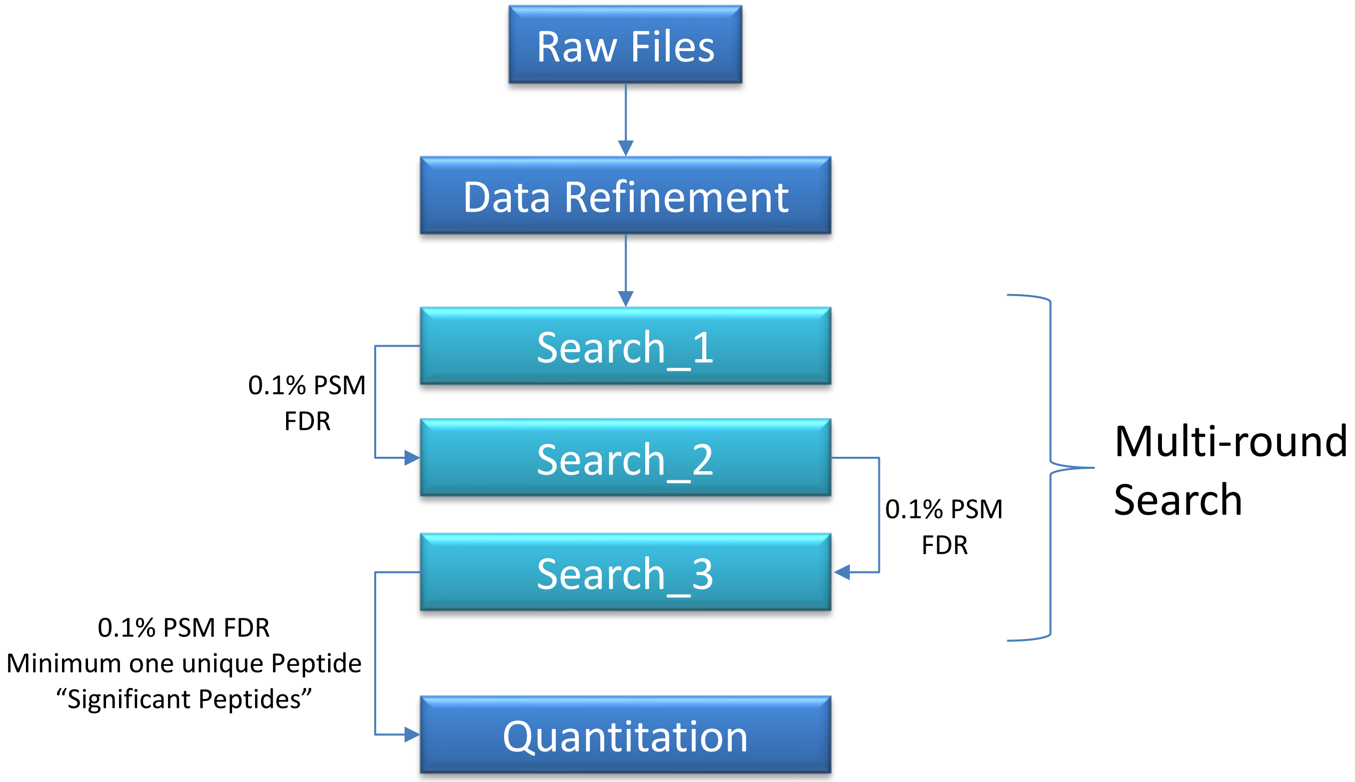


## Data Refinement

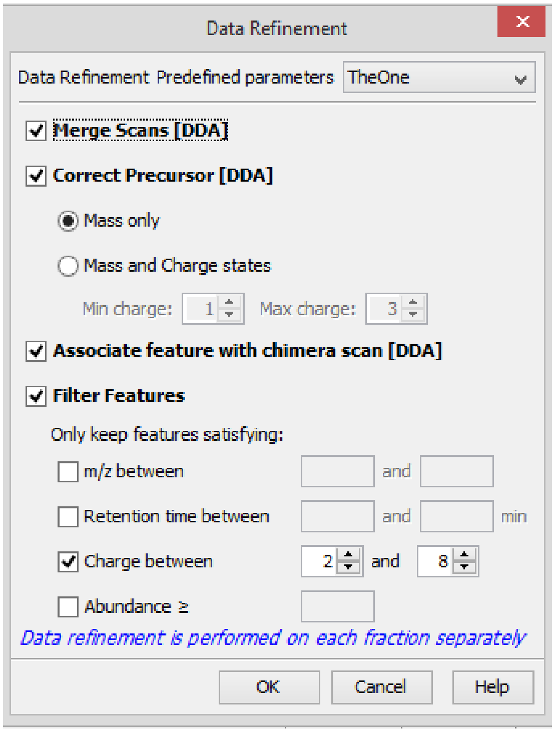


## Search_1

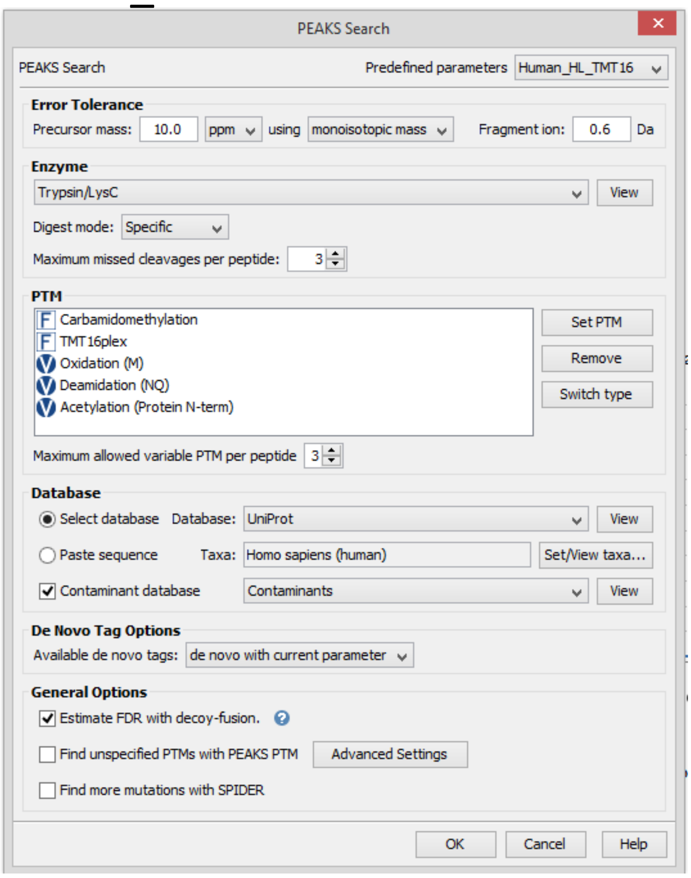

## Cleavage Rules

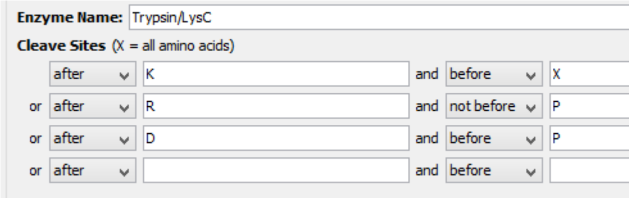


## Search_2

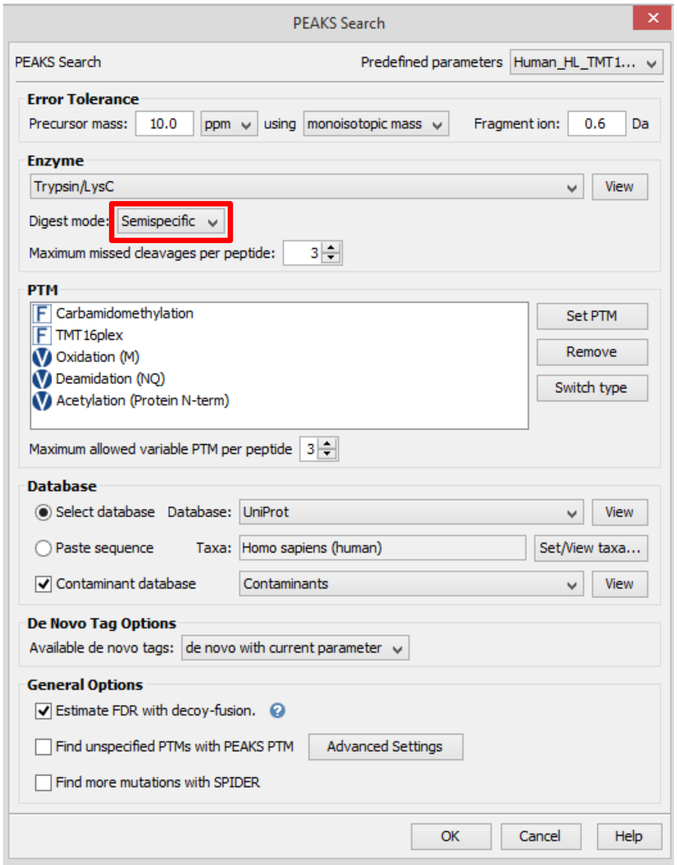


## Search_3

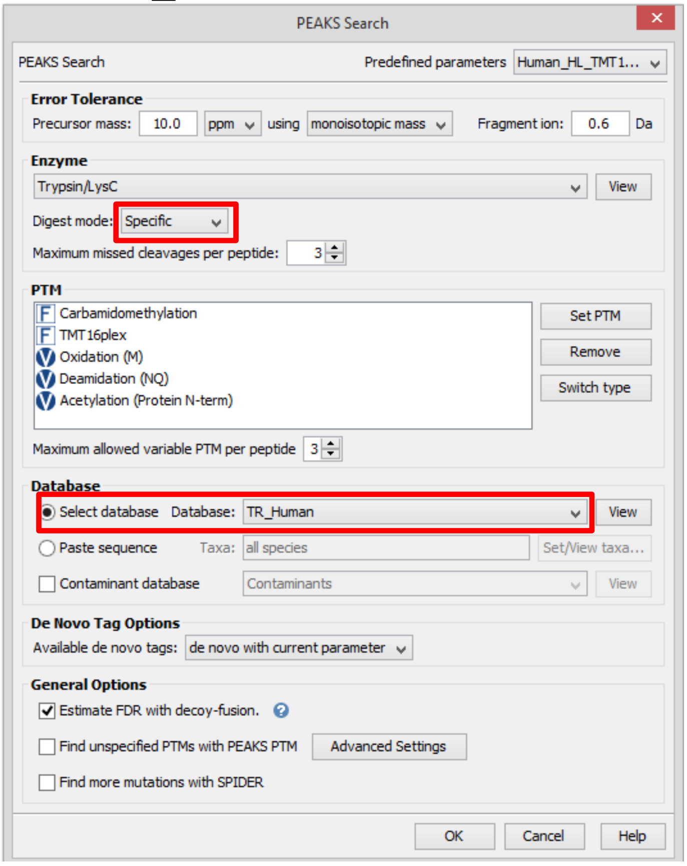


## Quantitation

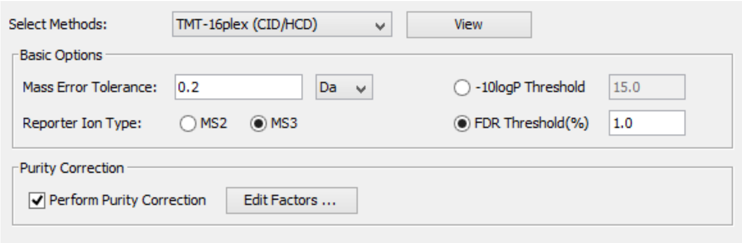


**Appendix Materials and Methods; Collection and processing of Mass spectrometry data.** Workflow (A) and data processing (B) of data represented in **Figure 1A & B** and **EV Fig 1A**.
