# Supplementary material for: Regulation of membrane fluidity by RNF145‐triggered degradation of the lipid hydrolase ADIPOR2
Source: EMBO J. 2022 Aug 22;41(19):e110777. doi: 10.15252/embj.2022110777 (PMC9531299; doi:10.15252/embj.2022110777)
Supplement: Supplementary file 2 — Expanded View Figures PDF [file EMBJ-41-e110777-s006.pdf]

Expanded View Figures

Figure EV1. RNF145 levels are regulated by FAs. Subcellular localisation of ADIPOR2.

A, B Whole-cell TMT-based quantitative proteomic analysis of HEK-293T cells treated as described in Fig 1A. Protein levels are expressed as relative abundance in PA vs BSA (A) or OA vs BSA-treated cells (B). Proteins with fold abundance changes ≥ |1.5| ($q \leq 0.05$) are labelled. ADIPOR2 and RNF145 are indicated in bold red. Proteins with roles in lipid metabolism and/or known to be fatty acid-regulated are shown in green. $N = 3$ biological replicates.

C RNF145 shows altered expression upon treatment with PA or OA. HEK-293T cells treated with PA/OA (400 µM) or BSA (vehicle control) for 6 h were examined by immunoblotting. An asterisk (*) indicates non-specific bands. $N = 3$ biological replicates.

D Validation of result in Fig 1B using independent HeLa single-cell clones knocked out for RNF145 (RNF145 KO #1–3). Since RNF145 and gp78 also coordinate the degradation of HMG-CoA reductase (HMGCR), we addressed whether gp78 is involved in ADIPOR2 degradation by knocking out gp78 (gp78 KO #1, #2) or RNF145 in combination with gp78 (RNF + gp78 KO #1–3). The stabilising effect of RNF145 on ADIPOR2 was not notably enhanced by concomitant gp78 knockout (lanes 7–9). An asterisk (*) indicates non-specific bands.

E Huh7 (i) and U2-OS cells (ii) were transfected with sgRNAs targeting either RNF145 (gRNF145) or B2M (gCTR) followed by immunoblot analysis of endogenous ADIPOR2. The asterisk (*) denotes non-specific bands.

F HEK-293T cells were transfected with 4 different sgRNAs targeting RNF145 (gRNF145 #1–4) or a sgRNA targeting B2M (gCTR). The asterisk (*) denotes non-specific bands. LE, long exposure.

G Regulation of RNF145 and ADIPOR2 in a primary cell line model. Primary human microvascular endothelial cells (MVECs) were supplemented with PA (400 µM), OA (400 µM) or BSA (vehicle control) for 6 h and RNF145 and ADIPOR2 expression analysed by immunoblotting.

H HEK-293T cells stably expressing a farnesylated-Em (mEmerald-Farnesyl-5) membrane marker and ADIPOR2-S were visualised by immunofluorescence microscopy. Micrographs ((a)–(c)) represent magnifications of the indicated areas in the merge panel. Blue arrows highlight areas of co-localisation between farnesyl-Em and ADIPOR2-S, possibly representing ER-plasma membrane contact sites. White arrows indicate regions where the ER and plasma membrane signals do not overlap. Scale bars = 10 µm or 5 µm ((a)–(c)).

© 2022 The Authors

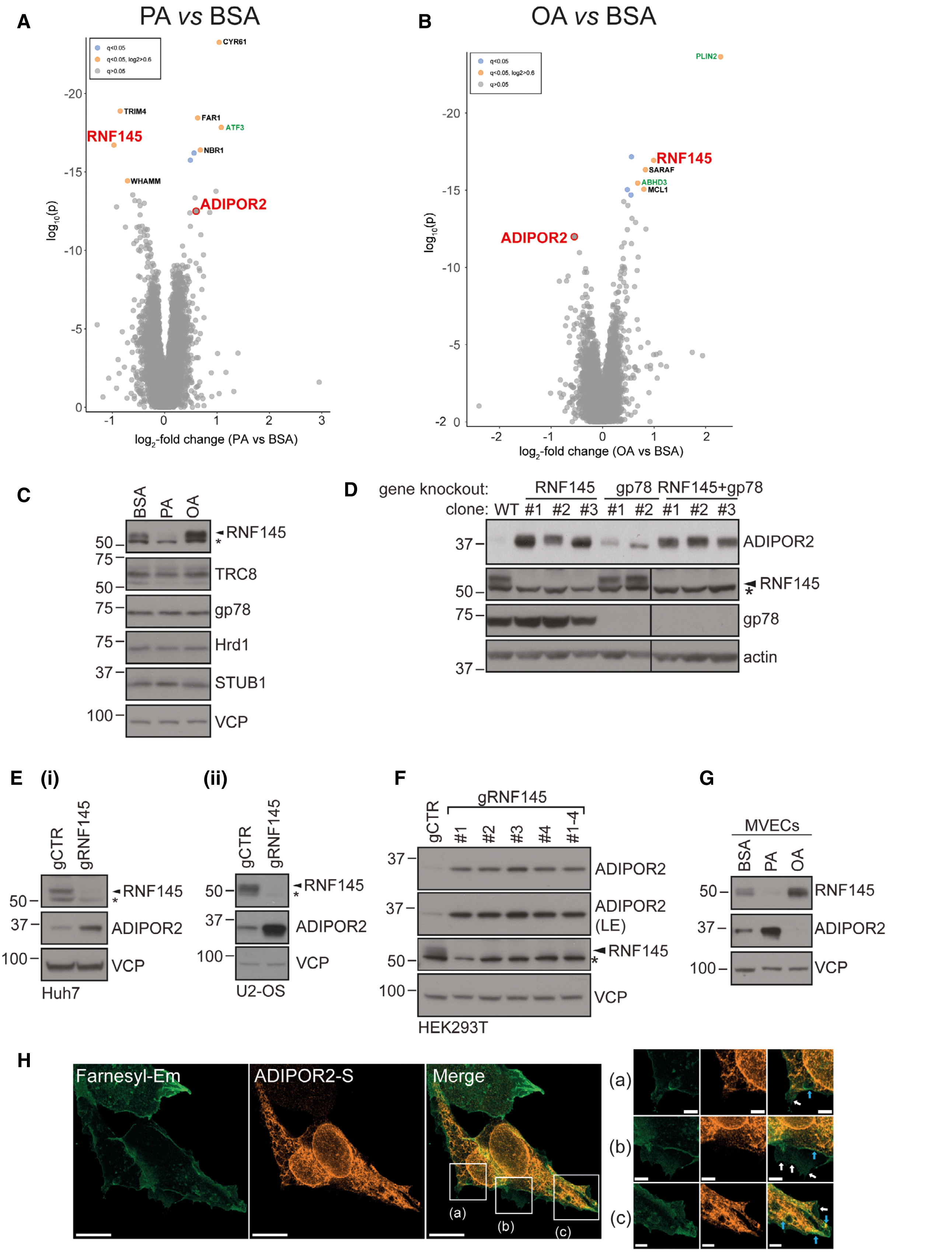

Figure EV1.

 © 2022 The Authors

Figure EV2. RNF145 depletion stabilises ADIPOR2-mRuby2 and ADIPOR2 is an ERAD substrate.

A ADIPOR2-depleted HEK-293T cells reconstituted with C-terminally mRuby2-tagged ADIPOR2 (ADIPOR2-HA-mRuby2) were depleted of RNF145 (gRNF145; red) or B2M (gCTR; blue) and fluorescence intensity was measured by flow cytometry.

B Immunoblot (IB) analysis of ADIPOR2-HA-mRuby2 expressed in ADIPOR2-depleted (gADIPOR2) cells. ADIPOR2-HA-mRuby2 was detected using a specific anti-HA antibody.

C ADIPOR2 is rapidly degraded at steady-state and can be rescued by proteasome inhibition. Representative ^{35}S pulse-chase in HeLa cells stably expressing ADIPOR2-HA ± MG132 (10 μg/ml, 2 h). *non-specific bands. Quantification of ^{35}S-labelled ADIPOR2-HA normalised to $t = 0$ h is shown below each lane.

D ADIPOR2-HA was stably expressed in HEK-293T cells, HA-affinity purified and interaction partners identified by tandem mass spectrometry. Proteins shown were not present in the negative control (empty vector expressing HEK-293Ts) and are therefore considered specific interaction partners of ADIPOR2-HA.

E Flow cytometry analysis of ADIPOR2-depleted HEK-293T cells complemented with ADIPOR2 tagged C-terminally with Clover (ADIPOR2-HA-Clover) and transfected with indicated sgRNAs. Knockdown efficiencies are shown in (ii). ADIPOR2-HA-Clover expression is shown in (iii). *non-specific bands.

F HEK-293T cells expressing ADIPOR2-HA-Clover were treated with VCP inhibitor (NMS-873, 10 μM, 6 h) or proteasome inhibitor (MG132, 10 μg/ml, 6 h) and ADIPOR2-HA-Clover expression was analysed by flow cytometry.

© 2022 The Authors

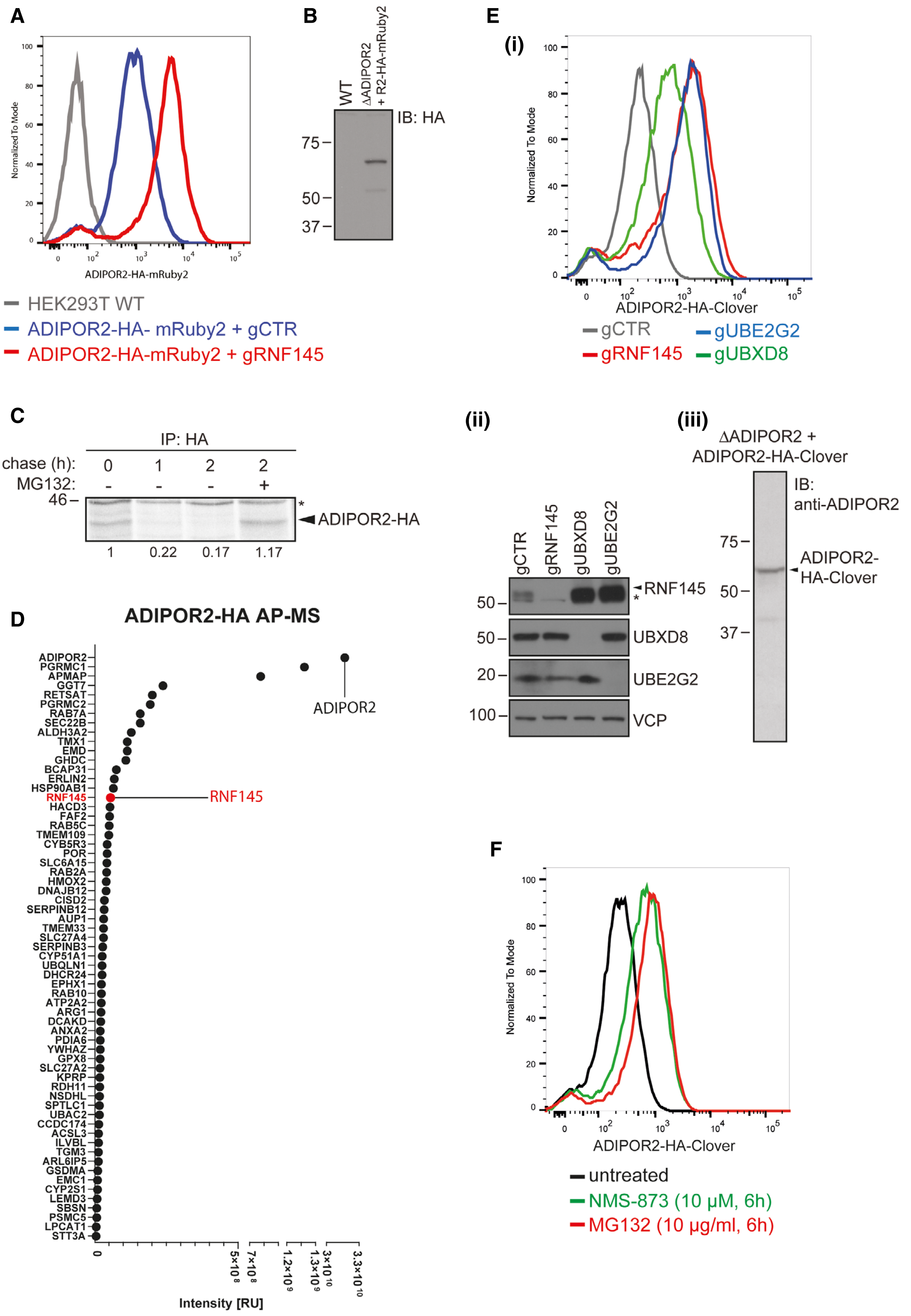

Figure EV2.

 © 2022 The Authors

Figure EV3. Regulation of RNF145 transcript and protein levels in the presence of excess OA/PA.

A HEK-293T cells were treated with PA, OA (each 400 μM, 6 h), or BSA (vehicle control). Quantitative PCR analysis for RNF145 was performed on whole-cell mRNA extracts, normalised to actin transcript levels and compared to BSA treatment (dotted line). Boxplots represent the median, first and third quantiles. Upper and lower whiskers extend to values up to 1.5-fold interquartile range. *P* values were calculated with a Wilcoxon rank-sum test. Benjamini-Hochberg-adjusted *P* values ≤ 0.05 are indicated (*).

B Assessment of the involvement of OA sensing by UBXD8 in the RNF145-mediated degradation of ADIPOR2. HEK-293T cells depleted of UBXD8 (gUBXD8) were stably complemented with an empty vector, UBXD8 (UBXD8 WT) or the OA-insensitive UBXD8^{K167E,R168E,R171E,K239E,R241E,R242E} mutant (mUBXD8), treated with PA or OA (200 μM, 6 h) and indicated proteins visualised by immunoblotting. *non-specific bands.

C Two clonal ADIPOR2 knockout HEK-293T cell lines (clone #1 or 2) or wild-type (WT) cells were treated with PA, OA or BSA vehicle control (veh.) and analysed by immunoblotting. LE, long exposure. *non-specific bands.

D Loss of UBE2G2 renders RNF145 levels fatty acid-independent. HEK293 cells were depleted of UBE2G2 (gUBE2G2) or B2M (gCTR) as described in Fig 2D and treated with BSA, PA or OA (200 μM) for 6 h. *non-specific bands.

E HeLa cells expressing HMGCR-Clover or HeLa HMGCR-Clover cells depleted of INSIG1/2 form Menzies *et al* (2018) were treated with BSA (control) or 400 μM PA or OA for 6 h, lysed and prepared for western blot with ADIPOR2 antibodies (i). (ii) Knockout of INSIG1 and 2 was confirmed using flow cytometry to measure HMGCR-Clover stabilisation. Cells were sterol depleted (DMEM +10% LPDS + 10 mM mevastatin + 50 mM mevalonate) for 20 h and treated with sterols (25-hydroxycholesterol (2 mg/ml), cholesterol (20 mg/ml)) for 2 h to trigger INSIG1/2-dependent HMGCR degradation.

© 2022 The Authors

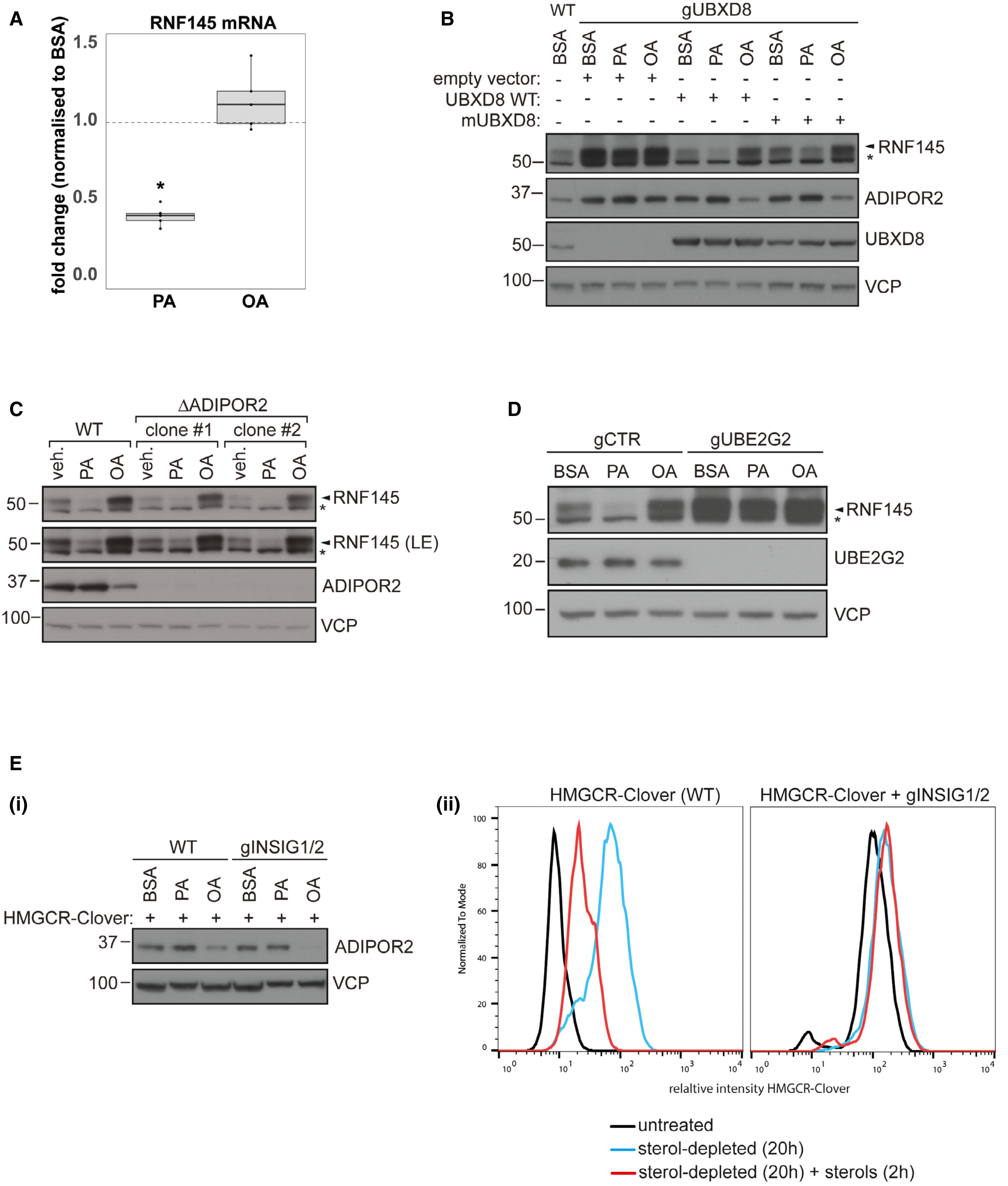

Figure EV3.

 © 2022 The Authors

Figure EV4. Quantitative analysis of lipids, lipid precursors and fatty acids in HEK-293T cells.

A Cells stably depleted of RNF145 (gRNF145), ADIPOR2 (gADIPOR2), or B2M (gCTR) were treated with BSA for 20h. All values were normalised to gCTR (dotted line). Boxplots represent the median, first and third quantiles. Upper and lower whiskers extend to values up to 1.5-fold interquartile range. $N = 6$ biological replicates. Changes were compared to gCTR and significance testing was performed using the Wilcoxon Rank Sum Test. $^{**}q \leq 0.01$.

B Lipidomic analysis of lipids containing deuterated PA (D3-PA) or its derivatives. Heat maps represent abundance changes relative to gCTR at the indicated time points. In PC (32:0), both side chains are deuterated. Statistical significance was calculated using one-way ANOVA. Benjamini-Hochberg-adjusted P-values ≤ 0.05 are indicated (*). PC, phosphatidylcholine; LPC, lysophosphatidylcholine; DAG, diacylglycerol; TAG, triacylglycerol.

© 2022 The Authors

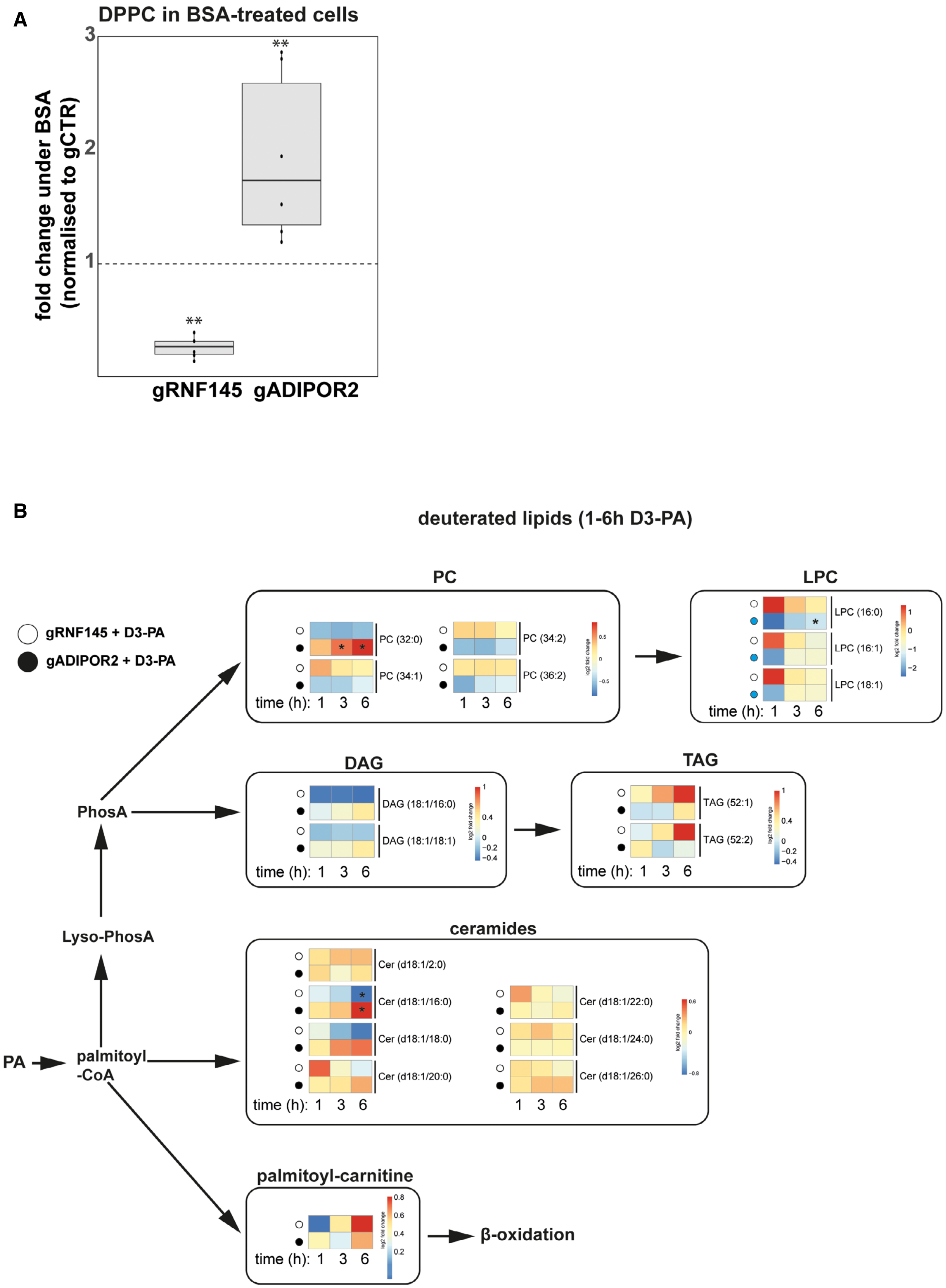

Figure EV4.

© 2022 The Authors
