# Supplementary material for: Regulation of membrane fluidity by RNF145‐triggered degradation of the lipid hydrolase ADIPOR2
Source: EMBO J. 2022 Aug 22;41(19):e110777. doi: 10.15252/embj.2022110777 (PMC9531299; doi:10.15252/embj.2022110777)
Supplement: Supplementary file 5 — PDF+ [file EMBJ-41-e110777-s002.pdf]

Article

TRANSPARENT PROCESS · OPEN ACCESS

THE EMBO JOURNAL

Regulation of membrane fluidity by RNF145-triggered degradation of the lipid hydrolase ADIPOR2

Norbert Volkmar[1,†,‡], Christian M Gawden-Bone[1,†], James C Williamson[1], Jonathon Nixon-Abell[2], James A West[1], Peter H St George-Hyslop[2], Arthur Kaser[1] & Paul J Lehner[1,*]

Abstract

The regulation of membrane lipid composition is critical for cellular homeostasis. Cells are particularly sensitive to phospholipid saturation, with increased saturation causing membrane rigidification and lipotoxicity. How mammalian cells sense membrane lipid composition and reverse fatty acid (FA)-induced membrane rigidification is poorly understood. Here we systematically identify proteins that differ between mammalian cells fed saturated versus unsaturated FAs. The most differentially expressed proteins were two ER-resident polytopic membrane proteins: the E3 ubiquitin ligase RNF145 and the lipid hydrolase ADIPOR2. In unsaturated lipid membranes, RNF145 is stable, promoting its lipid-sensitive interaction, ubiquitination and degradation of ADIPOR2. When membranes become enriched in saturated FAs, RNF145 is rapidly auto-ubiquitinated and degraded, stabilising ADIPOR2, whose hydrolase activity restores lipid homeostasis and prevents lipotoxicity. We therefore identify RNF145 as a FA-responsive ubiquitin ligase which, together with ADIPOR2, defines an autoregulatory pathway that controls cellular membrane lipid homeostasis and prevents acute lipotoxic stress.

Keywords adiponectin receptor 2; lipid homoestasis; membrane rigidity; palmitic acid; RNF145
Subject Category Post-translational Modifications & Proteolysis
DOI 10.15252/embj.2022110777 | Received 26 January 2022 | Revised 18 July 2022 | Accepted 20 July 2022 | Published online 22 August 2022
The EMBO Journal (2022) 41: e110777
See also: **C Alsayyah & R Ernst** (October 2022)

Introduction

The lipid composition of biological membranes dictates their fundamental physical and chemical properties including thickness, curvature, permeability and fluidity (Antonny *et al*, 2015; Harayama & Riezman, 2018). These properties enable essential cellular processes including protein assembly, folding, trafficking (Van Meer *et al*, 2008; Claypool, 2009; Antonny *et al*, 2015; Dowhan *et al*, 2019), and signal transduction (Sezgin *et al*, 2017; Gawden-Bone & Griffiths, 2019).

As an essential component of complex lipids, dietary fatty acids (FAs; composing the hydrophobic component of the lipid) determine multiple membrane properties (Borradaile *et al*, 2006). Through their incorporation into membrane phospholipids, FAs modulate cell membrane stiffness. High levels of saturated FAs increase membrane rigidity, due to enhanced lipid packing, while unsaturated FAs promote membrane fluidity by increasing lipid spacing (Holthuis & Menon, 2014).

To maintain optimal membrane fluidity under a constantly fluctuating dietary FA supply, complex homeostatic systems regulate membrane lipid content and preserve lipid bilayer integrity (Sinensky, 1974; Ernst *et al*, 2016; Levental *et al*, 2020). Prolonged exposure to high levels of saturated FAs, such as palmitic acid (PA), or genetic defects in lipid metabolising enzymes (Palareti *et al*, 2016; van Rijn *et al*, 2018) can impair or overwhelm these homeostatic mechanisms, triggering ER stress (Karaskov *et al*, 2006; Cunha *et al*, 2008; Pineau *et al*, 2009; Volmer & Ron, 2015), lipotoxicity (Weinberg, 2006; Ly *et al*, 2017) and ultimately cell death.

Proteins that sense changes in membrane lipid composition and facilitate the reversal of membrane rigidification are essential to avoid lipotoxicity. However, the homeostatic "sense-and-response" pathways required to maintain membrane lipid composition are not well understood in mammalian cells and are most comprehensively investigated for cholesterol. How cells sense membrane phospholipid saturation is unclear.

Studies in *S. cerevisiae*, *C. elegans* and human cell lines have identified multiple modulators of saturated FA-induced lipotoxicity (Ruiz *et al*, 2018, 2019a; Piccolis *et al*, 2019; Zhu *et al*, 2019). These include the transcription factors controlling lipid homeostasis (i.e. SREBP1), their major downstream targets (i.e. the desaturase SCD1, acyl-CoA synthases/transferases (ACATs, GPATs)), enzymes of the ubiquitin proteasome system (UPS) and adiponectin receptor 2 (ADIPOR2) (Piccolis *et al*, 2019; Zhu *et al*, 2019; Ruiz *et al*, 2019a).

1 Cambridge Institute of Therapeutic Immunology & Infectious Disease (CITIID), Jeffrey Cheah Biomedical Centre, University of Cambridge, Cambridge, UK
2 Cambridge Institute for Medical Research (CIMR), Cambridge, UK
*Corresponding author. Tel: +44 (0)1223 767800; E-mail: pjl30@cam.ac.uk
†These authors contributed equally to this work
‡Present address: Institute for Molecular Systems Biology (IMSB), ETH Zürich, Zürich, Switzerland

© 2022 The Authors. Published under the terms of the CC BY 4.0 license.

ADIPOR2 and its *C. elegans* orthologue Paqr-2 are lipid hydrolases that counteract saturated FA-induced membrane rigidification, although, how this is achieved and where in the cell this occurs are poorly understood (Pei *et al*, 2011; Svensk *et al*, 2013, 2016; Tanabe *et al*, 2015; Holland *et al*, 2017; Vasiliauskaité-Brooks *et al*, 2017; Chen *et al*, 2019; Zhu *et al*, 2019; Ruiz *et al*, 2019b; Jain *et al*, 2020).

Genetic deletion or ectopic expression of ADIPOR2 in rodent models of cardiovascular disease and diabetes alters tissue and systemic lipid metabolic status (Inukai *et al*, 2005; Bjursell *et al*, 2007; Liu *et al*, 2007; Yamauchi *et al*, 2007). Interestingly, Paqr-2 regulates membrane fluidity during cold shock and saturated lipid stress (Bodhicharla *et al*, 2018). Paqr-2 is functionally linked to Iglr-2 (Immunoglobulin domain and leucine-rich repeat protein-2), which promotes Paqr-2's lipid hydrolase activity in saturating conditions (Ruiz *et al*, 2021). While ADIPOR2 is critical for lipid homeostasis, little is known about how ADIPOR2 activity adjusts membranes to prevent an over-accumulation of saturated FAs. Mammals lack a direct Iglr-2 orthologue, so whether and how the expression and activity of the mammalian ADIPOR2 are regulated by exogenous FAs (or their derivatives) remain important, unresolved questions.

Here, we have taken an unbiased proteomic approach to identify master regulators of membrane lipid composition. We discovered a ubiquitin-based, lipid-sensitive, auto-regulatory pathway that prevents cellular lipotoxicity induced by saturated FAs. We identify ADIPOR2 as an ER-localised, novel substrate of the uniquely lipid-sensitive ER-resident E3 ubiquitin ligase RNF145. RNF145 is auto-regulated and adjusts its activity in response to changes in dietary FAs. We identify RNF145 as a ubiquitin-based, auto-regulatory rheostat that, together with ADIPOR2, maintains essential membrane properties to support cell viability.

Results

Fatty acids differentially regulate RNF145 and ADIPOR2

To determine how cells maintain their membrane lipid composition under high levels of dietary saturated or unsaturated FAs, we used tandem-mass tag (TMT)-assisted whole-cell proteomics in HEK-293T cells exposed to either palmitic acid (PA; C16:0) or oleic acid (OA, C18:1) (Fig 1A(i)). Out of 9,462 unique cellular proteins that were identified, 61 were FA-sensitive (≥ ±1.5-fold change, $q \leq 0.05$) (Figs 1A(ii) (orange) and EV1A and B). These differentially regulated proteins included many with known roles in lipid metabolism, such as perilipin-2 (PLIN2) (Xu *et al*, 2005; Masuda *et al*, 2006; Takahashi *et al*, 2016; Krahmer *et al*, 2018) and squalene monooxygenase (SQLE) (Stevenson *et al*, 2014), both of which were increased in OA (Fig 1A(ii), in green).

The ER-resident, polytopic ubiquitin E3 ligase RNF145 was one of the most differentially regulated proteins (3.9-fold decrease in PA vs OA, $q \leq 0.05$) (Fig 1A(ii), red). RNF145's sensitivity to FAs was unique and not seen with other ER-resident E3 ligases detected in either the proteomics dataset or by immunoblot analysis (e.g. gp78, Hrd1, the RNF145 homologue TRC8, or the cytoplasmic E3 ligase STUB1; Fig EV1C).

The FA-sensitive regulation of RNF145 (Fig 1A(ii), red) suggested that one or more of its substrates might be involved in the cellular response to altered membrane lipid composition. To identify proteins targeted for degradation by RNF145, we quantified changes in the proteome of three independent RNF145 knockout (KO) HeLa single cell clones. Out of 9,060 detected proteins, ADIPOR2 was the most marked and reproducibly upregulated protein (~ 4.3 fold increase) in the absence of RNF145 (Figs 1B, red and EV1D for immunoblot analysis).

Importantly, ADIPOR2 was also one of the top hits from the initial proteomics screen, where its FA sensitivity was reciprocal to that of RNF145 (Figs 1(ii) and EV1A and B), as would be predicted for an RNF145 substrate. ADIPOR2 expression increased following RNF145 depletion in multiple cell lines (Fig EV1E–G). The majority of S-tagged ADIPOR2 co-localised with an ER-restricted mEmerald marker (SP–KDEL-Em) (Fig 1C(i)) or with HA-tagged RNF145 which also localised in the ER (Fig 1C(ii)), but not with the plasma membrane marker farnesylated EGFP (Fig EV1H). Thus, in HEK-293T cells, ADIPOR2 is a predominantly ER-localised membrane protein that co-localises with RNF145.

The differential FA response of ADIPOR2 and RNF145 was also observed in primary human microvascular endothelial (MVEC) cells (Fig EV1G) and extended to FAs of varying chain length and saturation (Fig 1D). Reduced activity of the primary Δ9-desaturase

Figure 1. RNF145 and ADIPOR2 are fatty-acid responsive proteins that localise to the ER.

A Fatty-acid (FA) induced proteomic changes in HEK-293T cells. (i) Experimental workflow; cells were treated with PA (400 µM), OA (400 µM) or BSA (vehicle control) for 6 h in 3 independent experiments and proteomic changes determined by tandem mass tag (TMT)-assisted quantitative mass spectrometry. (ii) Quantitative analysis of proteomic changes in cells treated with PA or OA as described in (i). Proteins with significantly altered abundance (fold change ≥ |1.5|, q ≤ 0.05) are labelled. Proteins involved in lipid metabolism and/or known to be regulated by dietary FAs are indicated in green. RNF145 (bold red) and ADIPOR2 (bold green) were among "fatty-acid responsive" proteins. $N = 3$ biological replicates. A summary of the proteomics data is presented in Dataset EV1.

B ADIPOR2 is upregulated in RNF145-deficient cells. Quantitative proteomic analysis of HeLa RNF145 knockout (KO) cells *versus* wild type (WT) (i). ADIPOR2 is indicated in red (ii). Significantly changed proteins (q ≤ 0.05) are highlighted indicated (blue). $N = 3$ biological replicates.

C ADIPOR2 and RNF145 are primarily localised in the endoplasmic reticulum. HEK-293T cells stably expressing tagged constructs were used for immunofluorescence microscopy. (i) Staining of the ER marker protein SP-KDEL-Em (PDI signal peptide fused to mEMERALD-KDEL) and ADIPOR2-S. DNA was stained with DAPI. A magnified view is provided in the tiled micrographs (right). Scale bars = 10 µm / 5 µm (tiles). (ii) RNF45-HAx3 and ADIPOR2-S were stably expressed in UBE2G2-depleted cells and visualised using anti-HA and anti-S tag antibodies. DNA was stained using DAPI. A magnified view is provided in the tiled micrographs (right). Scale bars = 10 µm / 5 µm (tiles).

D RNF145 and ADIPOR2 respond to a range of FAs. HEK-293T cells were treated for 6 h with saturated FA (SFAs; 400 µM (C12:0 – C18:0); 200 µM (C20:0)) or unsaturated FAs (UFAs; 400 µM (C18:1-n9); 200 µM (C20:4-n6, C20:5-n3)). VCP serves as a loading control. *non-specific bands.

E Inhibition of the primary lipid desaturase SCD1 affects RNF145 and ADIPOR2 levels. HEK-293T cells were treated with the SCD1 inhibitor MF-438 (SCDi, 100 nM, 18 h) and RNF145/ADIPOR2 expression analysed by immunoblotting. LE, long exposure. *non-specific bands.

F SCD1 overexpression affects RNF145/ADIPOR2. HEK-293T cells containing an empty control vector or expressing SCD1-HA were cultured in FCS-free DMEM for 24 h and whole-cell lysates analysed. *non-specific bands.

© 2022 The Authors

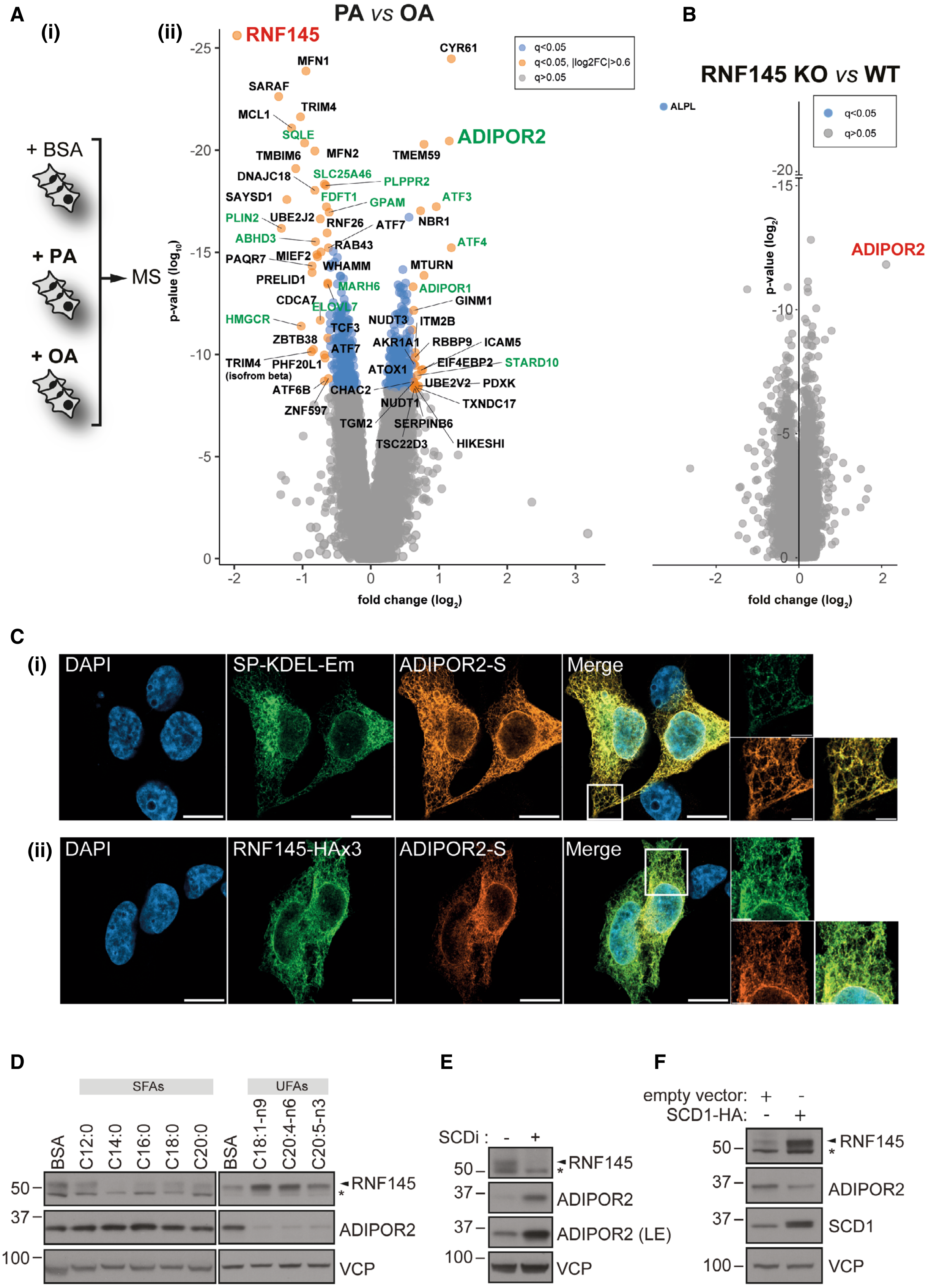
A
(i)
(ii)
PA vs OA
+ BSA
+ PA
+ OA
MS
RNF145
ADIPOR2
q<0.05
q<0.05, |log2FC|>0.6
q>0.05
p-value (log10)
fold change (log2)
B
RNF145 KO vs WT
ALPL
ADIPOR2
p-value (log2)
fold change (log2)
C
DAPI
SP-KDEL-Em
ADIPOR2-S
Merge
RNF145-HAx3
D
SFAs
UFAs
BSA
C12:0
C14:0
C16:0
C18:0
C20:0
C18:1-n9
C20:4-n6
C20:5-n3
RNF145
ADIPOR2
VCP
E
SCDi :
ADIPOR2 (LE)
F
empty vector:
SCD1-HA:
SCD1

Figure 1.

stearoyl-CoA desaturase 1 (SCD1) leads to an accumulation of saturated membrane lipids (Flowers & Ntambi, 2008; ALJohani *et al*, 2017). We therefore used pharmacological inhibition of SCD1 as an orthogonal approach to alter cellular membrane lipid saturation. The effect of SCD1 inhibition with MF-438 (SCDi) was similar to PA supplementation as it induced RNF145 loss and accumulation of ADIPOR2 (Fig 1E), while SCD1 overexpression had the inverse effect (Fig 1F). In summary, changing cellular FA/lipid levels consistently impacts RNF145 and ADIPOR2 protein expression in a reciprocal manner, providing a tissue-independent role for the ER-resident RNF145 E3 ligase in ADIPOR2 regulation and FA homeostasis.

RNF145 triggers ER-associated degradation of ADIPOR2

To determine whether endogenous, transcriptional control of ADIPOR2 is necessary for its modulation by RNF145, we expressed mRuby2-tagged ADIPOR2 under an ectopic promoter followed by RNF145 depletion. The accumulation of ADIPOR2 confirmed that RNF145-dependent regulation of ADIPOR2 was post-transcriptional (Fig EV2A and B). ^{35}S methionine/cysteine pulse chase revealed that endogenous ADIPOR2 had a very short half-life ($t_{1/2}$ = 34 ± 21.6 min) and was stabilised following the loss of RNF145 ($t_{1/2}$ = 117 ± 30.6 min) (Fig 2A) or proteasome inhibition with MG132 (Fig EV2C). This constitutive degradation of ADIPOR2 required the E3 ligase activity of RNF145, as a catalytically inactive RING-domain mutant RNF145$^{C552A, H554A}$-V5 (mRNF145-V5; Menzies *et al*, 2018) failed to degrade ADIPOR2 (Fig 2B). As mRNF145-V5 is more stable than RNF145-V5, this mutant allowed us to demonstrate an interaction between mRNF145-V5 and endogenous ADIPOR2 (Fig 2C). Immunoprecipitation of epitope-tagged ADIPOR2 confirmed its interaction with RNF145 by mass spectrometry (Fig EV2D, red).

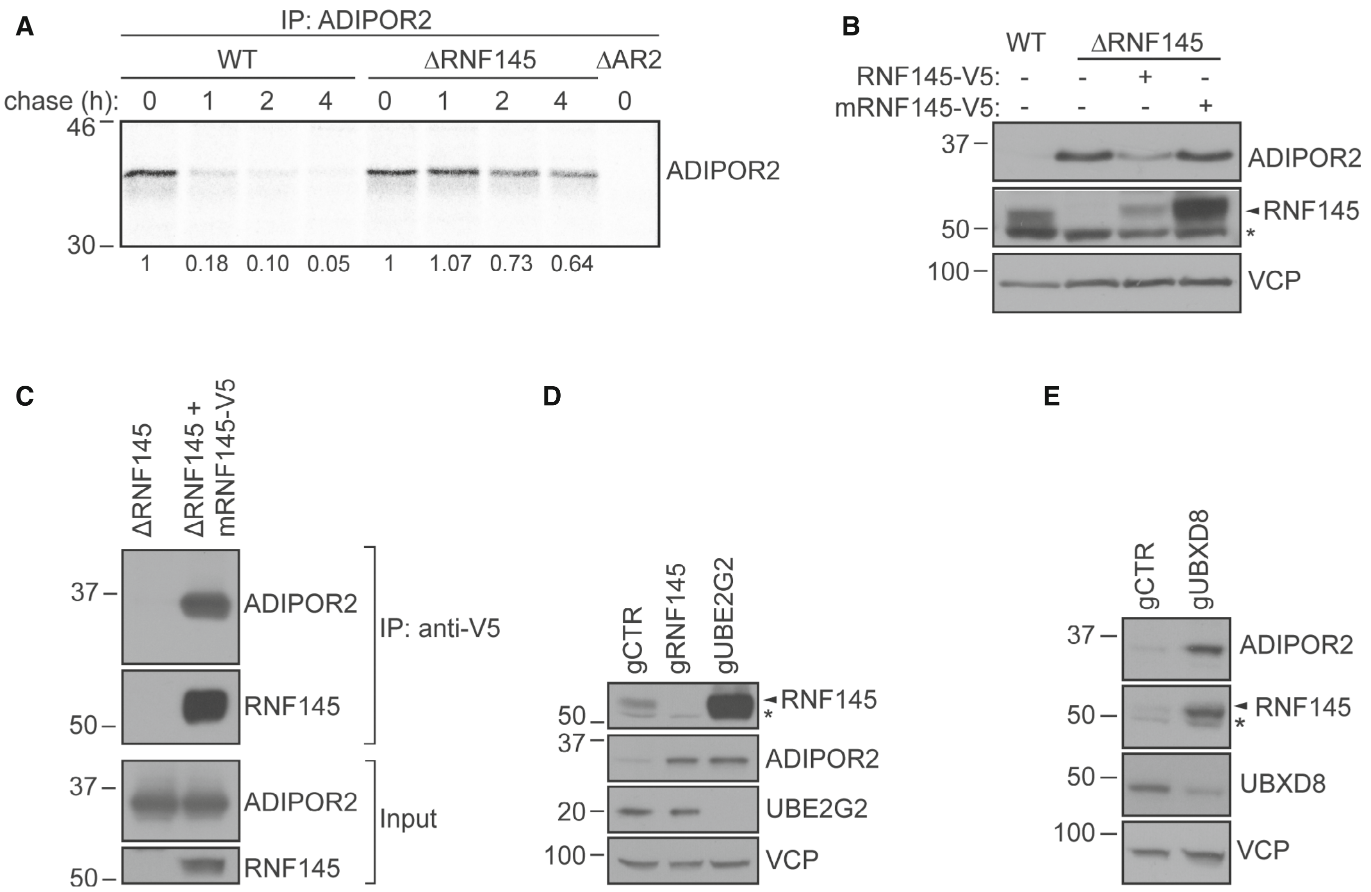

Figure 2. Stability of ADIPOR2 is dependent on the activity of RNF145, UBE2G2 and UBXD8.

A Endogenous ADIPOR2 is stabilised in RNF145 knockout cells. Representative ^{35}S pulse-chase analysis in HeLa WT, RNF145 knockout (ΔRNF145) or ADIPOR2 knockout (ΔAR2) cells. Quantification of ^{35}S-labelled ADIPOR2 normalised to t = 0 h is shown below.

B ADIPOR2 regulation requires the E3 ligase activity of RNF145. HEK-293T RNF145 knockout cells (ΔRNF145) were stably complemented with WT RNF145 (RNF145-V5) or an inactive RNF145^{C552A,H554A} RING domain mutant (mRNF145-V5). *non-specific bands.

C RNF145 binds ADIPOR2. RNF145 was immunoprecipitated from HeLa RNF145 knockout cells (ΔRNF145) complemented with WT RNF145 (RNF145-V5) or an inactive RNF145^{C552A,H554A} RING domain mutant (mRNF145-V5). Cells were treated with VCP inhibitor (NMS-873, 10 μM, 90 min) prior to immunoprecipitation with an anti-V5 antibody.

D The E2 ubiquitin-conjugating enzyme UBE2G2 is involved in RNF145-mediated ADIPOR2 degradation. Immunoblot analysis of HEK-293T cells depleted of RNF145 (gRNF145), UBE2G2 (gUBE2G2) or B2M (control; gCTR). *non-specific bands.

E ADIPOR2 degradation depends on UBXD8. HEK-293T cells were depleted of B2M (gCTR) or UBXD8 (gUBXD8) and analysed by immunoblotting. *non-specific bands.

© 2022 The Authors

These findings show that RNF145 physically interacts with ADIPOR2, mediates its ubiquitination, and initiates ADIPOR2's entry into the ER-associated degradation (ERAD) pathway (Stevenson *et al*, 2016). RING-type E3 ubiquitin ligases do not themselves bind ubiquitin but associate with their substrate and mediate the transfer of ubiquitin from their E2 ubiquitin-conjugating enzymes. Depletion of UBE2G2, the cognate E2 ubiquitin-conjugating enzyme of RNF145 (Menzies *et al*, 2018), stabilised endogenous ADIPOR2 (Fig 2D). A multiprotein complex that includes the AAA ATPase VCP and the ubiquitin adaptor and VCP cofactor UBXD8, cooperates with E3 ligases by extracting ubiquitinated proteins from the ER membrane for proteasome-dependent degradation. Accordingly, ADIPOR2 was stabilised by either depletion of UBXD8 or pharmacological VCP inhibition (Alexandru *et al*, 2008; Schuberth & Buchberger, 2008; Figs 2E and EV2E and F).

Thus, ADIPOR2 is a substrate of ERAD, with the UBE2G2-RNF145 complex mediating its recognition and ubiquitination to initiate degradation.

RNF145 auto-degradation is responsive to fatty acids

During our analysis of ADIPOR2 degradation, we found that RNF145 is itself subject to ERAD. In the presence of PA, the low expression levels of RNF145 were stabilised by proteasome inhibition or UBXD8 depletion (Fig 3A and B). Similarly, RNF145 steady-state degradation was strongly impaired when inactivating mutations were introduced into its RING domain (Menzies *et al*, 2018). These findings suggested that RNF145 triggers its own auto-ubiquitination and ERAD, which is regulated by FAs.

We therefore tested whether cellular lipid composition affected RNF145 ubiquitination. In PA, endogenous RNF145 polyubiquitination increased, and was lost following the addition of OA (Fig 3C). Cycloheximide (CHX) chase experiments confirmed that in OA, RNF145 was stabilised (Fig 3D) with no significant effect on RNF145 transcript levels (fold increase: 1.14 ± 0.19 SD), which decreased in PA (Fig EV3A). Substitution of all 22 RNF145 lysine residues with arginine residues (RNF145-V5 K>R) stabilised the ligase, rendering it much less responsive to changes in FA composition (Fig 3E). To further evaluate the role of RNF145 auto-ubiquitination, we compared expression of ectopic wild-type RNF145-V5, with the RING-domain mutant (mRNF145-V5) in RNF145 KO cells. In PA, ectopic wild-type RNF145-V5 was degraded while expression of mRNF145-V5 was unchanged (Fig 3F). These changes in RNF145 therefore reflect its rapid FA-induced auto-ubiquitination and are critical for regulating its abundance in response to changes in membrane lipid composition.

UBXD8, which links ubiquitinated proteins with the VCP complex, is required for the degradation of RNF145 and ADIPOR2 (Fig 2E). Unsaturated FAs are known to reduce binding between UBXD8 and INSIG1 and leading to reduced INSIG1 extraction from the ER membrane and consequently its stabilisation (Lee *et al*, 2008, 2010; Kim *et al*, 2013). To test whether a similar UBXD8-mediated mechanism might operate in the stabilisation and accumulation of RNF145 in response to OA, we complemented UBXD8-depleted cells with either WT UBXD8 or an FA-insensitive UBXD8 mutant (mUBXD8, Kim *et al*, 2013) and challenged them with OA. While reintroduction of the FA-insensitive UBXD8 variant lead to a global decrease in RNF145 levels, it could not prevent OA-mediated RNF145 stabilisation (Fig EV3B). These findings suggest that UBXD8 does not directly regulate the OA-induced engagement of ADIPOR2 by RNF145.

The lipid-sensitive degradation of ADIPOR2 is RNF145-dependent

Our finding that ADIPOR2 interacts with RNF145, together with the observation that RNF145 degradation is under FA control, implies that FA-triggered changes in ADIPOR2 expression are likely to be a direct consequence of changes in RNF145. Consistent with this model, in the absence of RNF145, ADIPOR2 levels were stable and completely unresponsive to FA changes (PA/OA) (Fig 4A, cf. lanes 2, 3 and 5, 6). By contrast, loss of ADIPOR2 did not affect RNF145's sensitivity to changes in dietary FAs (Fig EV3C). FA-induced alterations in ADIPOR2 abundance are therefore dependent on the activity of RNF145, indicating RNF145 senses changes in membrane lipid composition and regulates ADIPOR2 levels in response to these changes.

It was therefore important to determine whether RNF145's interaction with ADIPOR2 (Fig 2C) is itself lipid-sensitive, providing an additional mechanism of regulation of ADIPOR2 activity. We initially had to stabilise endogenous RNF145 so that it was itself insensitive to changes in FAs, an effect achieved by depleting cells of UBE2G2, RNF145's cognate E2 (Fig EV3D). Under these conditions, RNF145 remained stable, unresponsive to PA or OA but showed a clear lipid-sensitive interaction with ADIPOR2 (Fig 4B). Binding of RNF145 to ADIPOR2 was increased in OA but attenuated in PA (Fig 4B, cf. lane 3 and 4–5 for BSA vs PA/OA). Consistent with this finding, the RNF145-dependent ubiquitination of ADIPOR2 was also increased in OA-treated cells (Fig 4C) and ADIPOR2 was stabilised under OA conditions when all lysine residues in ADIPOR2 were mutated to arginine residues (Fig 4D).

The insulin-induced genes 1 and 2 (INSIG1/2) are intermediaries, allowing RNF145 to interact with its substrate 3-hydroxy-3-methylglutaryl-coenzyme A reductase (HMGCR) (Jiang *et al*, 2018; Menzies *et al*, 2018). We therefore tested whether the INSIG proteins were required for the RNF145-dependent regulation of ADIPOR2 and show that a double knockout of INSIG1/2, did not affect the RNF145-mediated regulation of ADIPOR2 (Fig EV3E).

In summary, OA promotes the interaction between ADIPOR2 and its E3 ligase RNF145, stimulating ADIPOR2 ubiquitination via UBE2G2, dislocation (via VCP and UBXD8) and resulting in proteasome-mediated turnover of ADIPOR2. In contrast, in PA, RNF145 is unable to interact with ADIPOR2, leading to its autodegradation, thus promoting ADIPOR2 stability (Fig 4E).

RNF145 and ADIPOR2 maintain membrane lipid homeostasis

To understand the involvement of RNF145 and ADIPOR2 in FA metabolism and lipotoxicity, we measured cell viability under high PA concentrations. Prolonged exposure (96 h) of ADIPOR2-depleted cells to PA markedly enhanced lipotoxic cell death, while in identical conditions viability increased in RNF145-deficient cells (Fig 5A; Appendix Fig S1A for knockdown efficiencies). Despite prolonged PA treatment, ADIPOR2 in control cells (gCTR) never reached the high levels of ADIPOR2 expression seen in the complete absence of RNF145 (Fig 5B, compare lanes 3 and 4). Furthermore, the subsequent depletion of RNF145 from ADIPOR2-deficient cells failed to

© 2022 The Authors

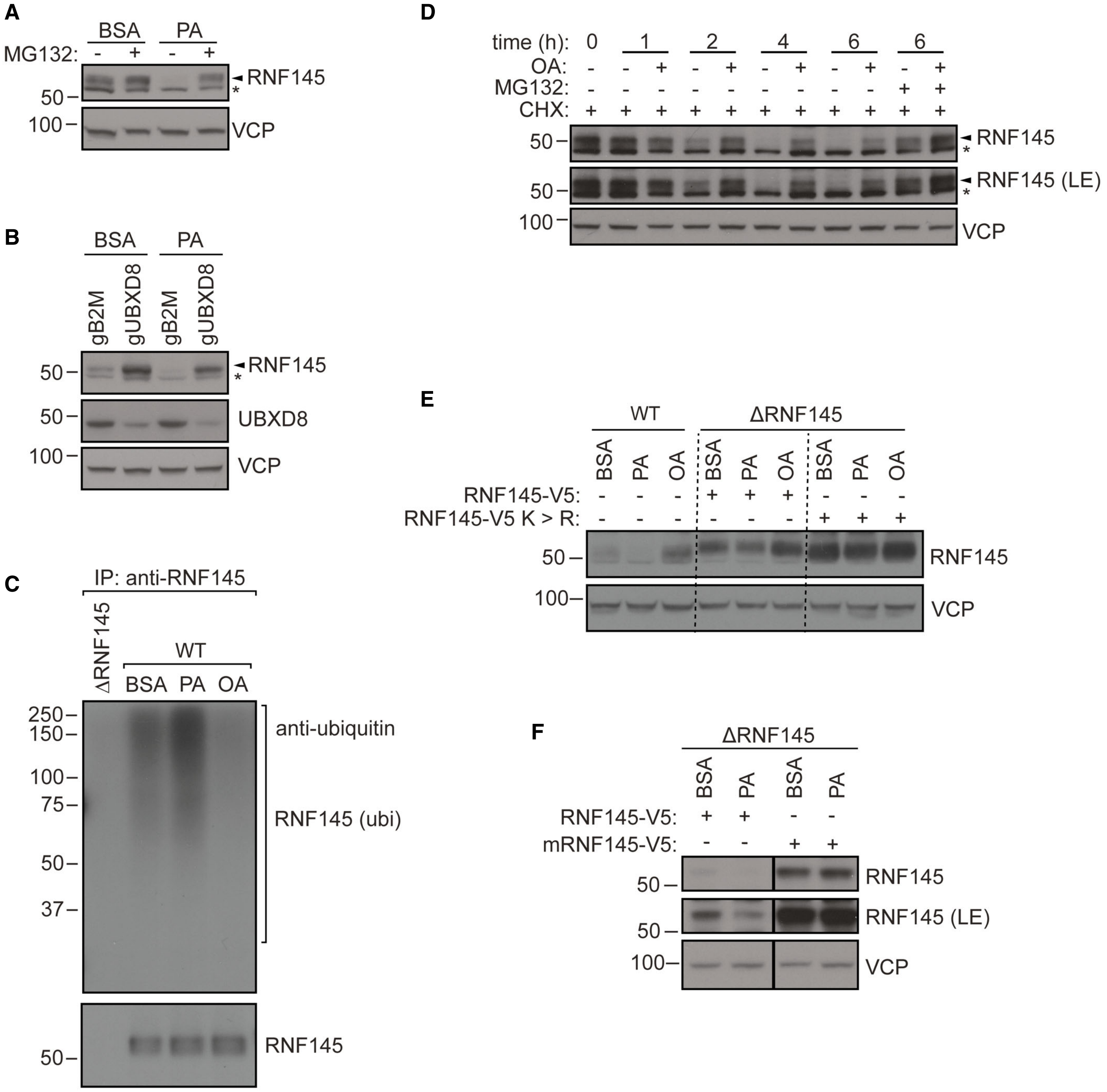

Figure 3. ADIPOR2's fatty acid response is modulated by RNF145.

- A Fatty acid-mediated regulation of RNF145 is proteasome-dependent. HEK-293T cells were treated with BSA, PA (400 μM, 6 h) ± MG132 (10 μg/ml, 6 h) and analysed by immunoblotting. *non-specific bands.
- B UBXD8 participates in PA-modulated RNF145 degradation. CRISPR/Cas9-mediated depletion of UBXD8 (gUBXD8) or B2M (gB2M, control) in HEK-293T cells, followed by exposure to PA (400 μM, 6 h) or BSA (6 h, vehicle control). *non-specific bands.
- C PA and OA treatment alter RNF145 ubiquitination. Immunoprecipitation of RNF145 from cells treated with BSA (control), PA or OA (each 200 μM, 3 h). During fatty acid treatments, VCP inhibitor (NMS-873, 10 μM) was present to stabilise ubiquitinated proteins. RNF145 ubiquitination (ubi) was detected using an anti-ubiquitin antibody.
- D HEK-293T cells were treated with cycloheximide (CHX, 1 μg/ml) and OA (400 μM, 6 h) ± MG132 (10 μg/ml) for the indicated times. LE, long exposure; *non-specific bands.
- E Lysine-less RNF145 is stabilised under PA treatment. RNF145 knockout HEK-293T cells (ΔRNF145) were complemented with RNF145-V5 or a lysine-less variant of RNF145 (RNF145-V5K>R) and treated with BSA or PA (400 μM, 6 h). Dotted lines demarcate the different cell lines.
- F Adjustment of RNF145 levels to PA requires its ubiquitin ligase activity. Immunoblot of whole-cell lysates from RNF145 knockout HEK-293T (ΔRNF145) cells stably complemented with RNF145-V5 or RNF145$^{C552A,\ H554A}$-V5 (mRNF145-V5) and treated with BSA or PA (400 μM, 6 h). LE, long exposure.

 © 2022 The Authors

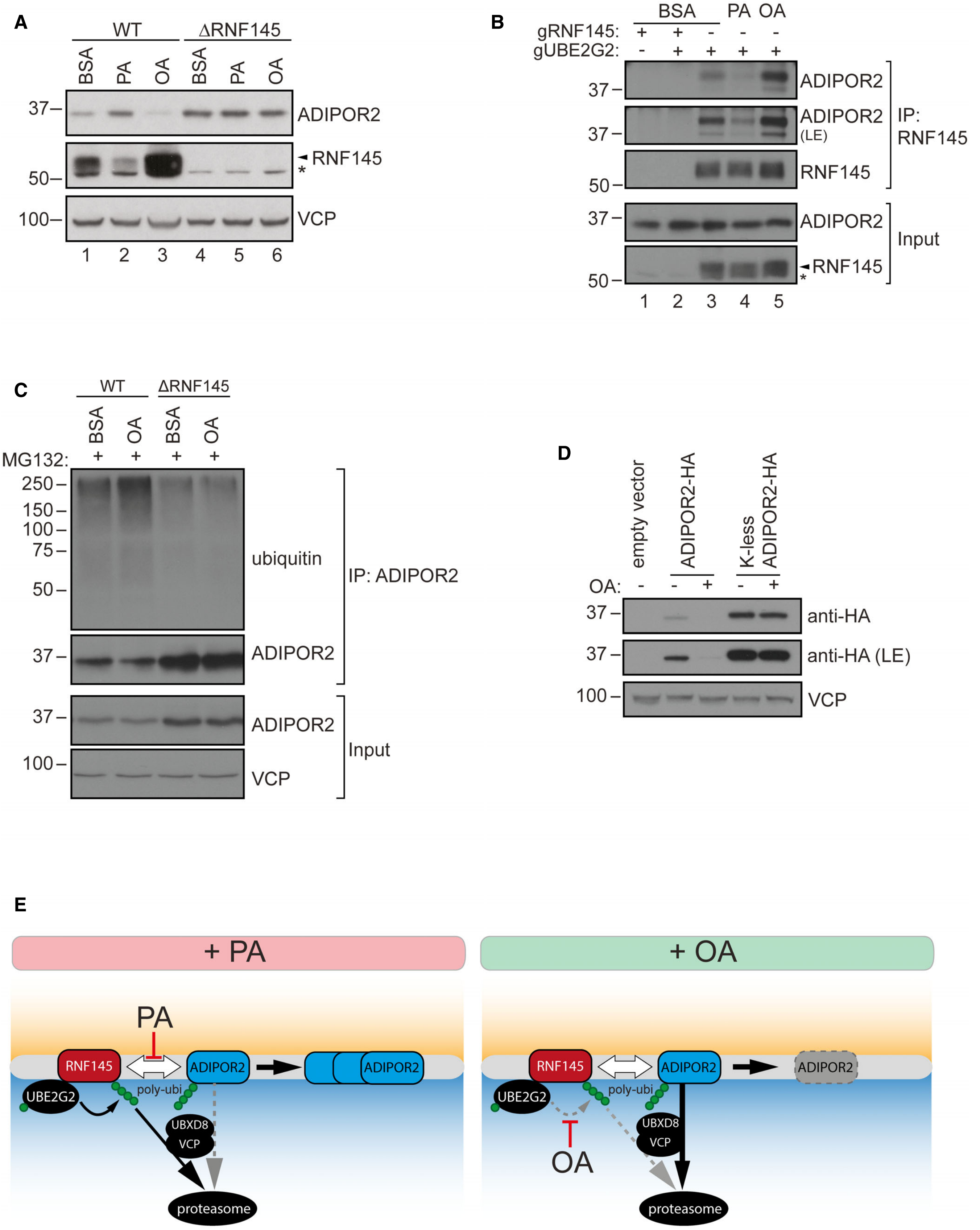
A
WT
ΔRNF145
BSA
PA
OA
BSA
PA
OA
37
ADIPOR2
RNF145
*
50
100
VCP
1 2 3 4 5 6
B
BSA
PA OA
gRNF145: + + - - -
gUBE2G2: - + + + +
37
ADIPOR2
ADIPOR2 (LE)
IP: RNF145
RNF145
50
ADIPOR2
Input
RNF145
*
1 2 3 4 5
C
WT
ΔRNF145
BSA OA BSA OA
MG132: + + + +
250
150
100
75
50
ubiquitin
IP: ADIPOR2
37
ADIPOR2
ADIPOR2
Input
100
VCP
D
empty vector
ADIPOR2-HA
K-less ADIPOR2-HA
OA: - - + - +
anti-HA
anti-HA (LE)
VCP
E
+ PA
+ OA
PA
RNF145
ADIPOR2
UBE2G2
poly-ubi
UBXD8
VCP
proteasome
OA

Figure 4.

◀ **Figure 4. The interaction between RNF145 and ADIPOR2 is fatty acid-sensitive.**

A ADIPOR2 regulation by fatty acids is completely dependent on RNF145. WT or RNF145 knockout HEK-293T cells (ΔRNF145) were treated with BSA, PA or OA (400 µM, 6 h) and analysed by immunoblotting. Non-specific bands are indicated (*).

B RNF145 – ADIPOR2 interaction is fatty acid-modulated. Immunoprecipitation (IP) of endogenous RNF145 from HEK-293T cells depleted of UBE2G2 (gUBE2G2) and/or RNF145 (gRNF145) and treated for 3 h with 400 µM PA, OA or BSA (control). LE, long exposure. *non-specific bands.

C Immunoprecipitation (IP) of endogenous ADIPOR2 from WT or ΔRNF145 cells treated with MG132 (5 µg/ml, 6 h) ± OA (200 µM, 6 h). Ubiquitinated ADIPOR2 was detected using a specific anti-ubiquitin antibody (clone VU-1). Note that ADIPOR2 levels in RNF145 knockout HEK-293T cells are increased.

D ADIPOR2 knockout HEK-293T cells were stably complemented with ADIPOR2-HA, an ADIPOR2 mutant in which all lysines were mutated to arginines (K-less ADIPOR2-HA), or an empty vector control and exposed to OA (200 µM, 6 h) or BSA (control). ADIPOR2 variants were visualised by immunoblotting using a specific anti-HA antibody. LE, long exposure.

E Model of RNF145-mediated ADIPOR2 degradation. RNF145 uses UBE2G2 to target ADIPOR2 for UBXD8/VCP-mediated extraction from the ER membrane and subsequent proteasomal degradation. PA decreases RNF145 binding to ADIPOR2, while OA promotes ADIPOR2's interaction with and ubiquitination by RNF145 (white arrows).

increase cell viability, showing that RNF145's protective effect is entirely dependent on ADIPOR2 (Fig 5A). The PA sensitivity of ADIPOR2-deficient cells could be rescued by re-expression of wild type, but not catalytically inactive, HA-tagged ADIPOR2 (Fig 5C; Appendix Fig S1B for expression levels). The increased lipid hydrolase activity associated with increased ADIPOR2 expression in RNF145 KO cells is therefore essential for preventing lipotoxicity.

To determine how ADIPOR2's lipid hydrolase activity maintains membrane homeostasis, we performed quantitative lipidomics. The addition of deuterated PA allowed us to trace the fate of dietary PA in cells lacking ADIPOR2 or expressing the catalytically dead version of the enzyme. In these cells, PA was diverted into the production of the membrane-rigidifying phospholipid dipalmitoylphosphatidylcholine (PC(16:0,16:0)/DPPC) and ceramides (Cer (d18:1/16:0), Cer (d18:1/20:0)) (Fig 5D). Furthermore, storage lipids (triacylglycerides, TAG(52:2)) that normally sequester otherwise cytotoxic FAs were reduced in ADIPOR2-deficient cells (Fig 5D). Therefore, ADIPOR2's catalytic activity counteracts the accumulation of cytotoxic lipid species such as DPPC or long-chain saturated sphingolipids.

Phosphatidylcholines (PCs) are among the most abundant structural membrane lipids, constituting approximately 40 mol% of lipid mass (Vance, 2015; Yang *et al*, 2018). Lipid metabolic profiling in RNF145-deficient cells, where ADIPOR2 expression is high, revealed a marked reduction in DPPC (~ 2.7-fold decrease ± 0.151 SD), while DPPC accumulated in ADIPOR2-deficient cells (~ 5.9-fold increase ± 2.2 SD) (Fig 5E). The same trends were already evident at a lower magnitude in vehicle control (BSA)-treated cells (Fig EV4A). Taken together, our findings show that in ADIPOR2-deficient cells, PA supplementation aggravates an already underlying lipid imbalance.

To differentiate changes in *de novo* lipid biosynthesis from pre-existing lipid pools, we traced and quantified deuterated PA incorporation in RNF145- or ADIPOR2-depleted cells over a 6h period. In ADIPOR2-depleted cells, labelled PA rapidly accumulated in DPPC 2.9 ± 0.16 fold ($q \leq 0.05$) (Fig EV4B) with reduced PA conversion to unsaturated PCs (e.g. PC(34:1), PC(34:2), PC(36:2)) and increased PA shunting into multiple long-chain saturated ceramides (Cer(d18:1/16:0), Cer(d18:1/18:0), Cer(d18:1/20:0)). In contrast, RNF145-depleted cells showed less incorporation of PA into DPPC and ceramides, while storage of incoming PA in *de novo* synthesised triacylglycerides (TAG(52:1), TAG(52:2)) was increased (Fig EV4B).

Therefore, RNF145 mediates the processing of saturated FAs through its regulation of ADIPOR2. Our data strongly indicate that RNF145-sensed changes in the lipid properties of the membrane coordinate ADIPOR2 expression. ADIPOR2 in turn regulates DPPCs, saturated acyl chains and ceramides to prevent lipotoxicity and

Figure 5. RNF145 depletion attenuates PA-induced cytotoxicity and promotes membrane fluidity. ▶

A Loss of ADIPOR2 sensitises cells to PA-induced cytotoxicity. HEK-293T cells were depleted of RNF145 (purple), ADIPOR2 (blue), B2M (CTR; grey), or both ADIPOR2 and RNF145 (blue + purple), exposed to PA (200 µM, 96 h) and stained with crystal violet. Quantification of crystal-violet stained cells is shown alongside a representative micrograph (below). The response of each cell line to PA was normalised to its BSA-treated condition (dotted line). Statistically significant differences to cells containing gCTR are indicated (robust one-way ANOVA with Benjamini-Hochberg multiple testing correction). Boxplots represent the median, first and third quantiles. Upper and lower whiskers extend to values up to 1.5-fold interquartile range. $N = 3$ biological replicates, $**q \leq 0.01$. Double knockdowns were generated by sequential constitutive CRISPR/Cas9-mediated depletion of RNF145 and ADIPOR2. Knockdown efficiencies are shown in Appendix Fig S1A.

B Cells were treated as in (A) and lysates analysed by immunoblotting. LE, long exposure; *non-specific bands.

C HEK-293T cells depleted of B2M (gCTR) or ADIPOR2 (gADIPOR2) were complemented with empty vector (blue), full-length ADIPOR2-HA (green), or a catalytically inactive variant of ADIPOR2-HA (dead ADIPOR2; red) and treated with PA (200 µM, 96 h) or BSA (vehicle control, 96 h). Measurements from PA-treated cells were normalised to their BSA-treated condition (dotted line). Cell growth was quantified, and representative crystal violet stainings are shown. A steady-state anti-HA immunoblot of cell lines used is shown in Appendix Fig S1B. $N = 3$–5 biological replicates. Boxplots represent the median, first and third quantiles. Upper and lower whiskers extend to values up to 1.5-fold interquartile range. $**q \leq 0.01$ (robust one-way ANOVA with Benjamini-Hochberg multiple testing correction).

D ADIPOR2's hydrolytic activity is necessary to prevent excessive accumulation of saturated lipids. HEK-293T cells depleted of ADIPOR2 (gADIPOR2; blue) or B2M (gCTR) were stably complemented with an empty vector control, full-length ADIPOR2-HA (green), or catalytically inactive ADIPOR2-HA (red) and incubated with deuterated PA (D3-PA) for 6 h. Deuterated lipids derived from internalised D3-PA were quantified by mass spectrometry. The indicated lipids were normalised to the gCTR condition. $N = 6$ biological replicates. Boxplots represent the median, first and third quantiles. Upper and lower whiskers extend to values up to 1.5-fold interquartile range. $*q \leq 0.05$ (one-way ANOVA with *post-hoc* Benjamini-Hochberg multiple testing correction).

E Lipidomic analysis of phosphatidylcholines in HEK-293T cells depleted of RNF145 (gRNF145; white), ADIPOR2 (gADIPOR2; grey), or B2M (gCTR) and subjected to PA treatment (150 µM, 20 h). Chain length and saturation/desaturation are indicated. Relative lipid abundance was obtained by normalising to absolute lipid abundance in gCTR cells. Statistically significant differences to gCTR are shown (Wilcoxon Rank Sum Test with Benjamini-Hochberg multiple testing correction). Mean ± SD are shown. Boxplots represent the median, first and third quantiles. Upper and lower whiskers extend to values up to 1.5-fold interquartile range. $N = 6$ biological replicates, $*q \leq 0.05$.

 © 2022 The Authors

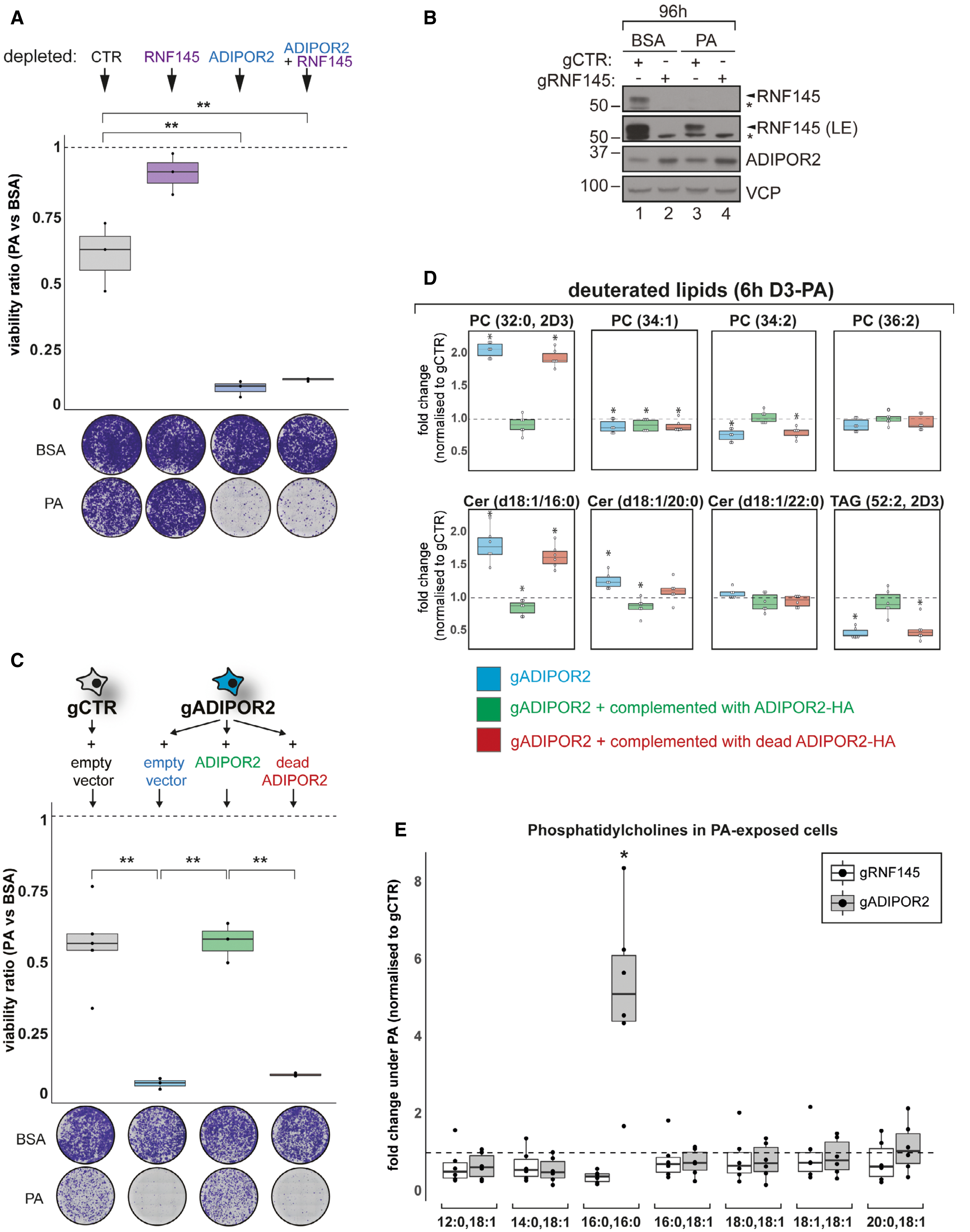
A
depleted: CTR RNF145 ADIPOR2 ADIPOR2 + RNF145
viability ratio (PA vs BSA)
BSA
PA
B
96h
BSA PA
gCTR: + - + -
gRNF145: - + - +
RNF145
RNF145 (LE)
ADIPOR2
VCP
1 2 3 4
C
gCTR
gADIPOR2
empty vector
empty vector
ADIPOR2
dead ADIPOR2
viability ratio (PA vs BSA)
BSA
PA
D
deuterated lipids (6h D3-PA)
PC (32:0, 2D3)
PC (34:1)
PC (34:2)
PC (36:2)
Cer (d18:1/16:0)
Cer (d18:1/20:0)
Cer (d18:1/22:0)
TAG (52:2, 2D3)
fold change (normalised to gCTR)
gADIPOR2
gADIPOR2 + complemented with ADIPOR2-HA
gADIPOR2 + complemented with dead ADIPOR2-HA
E
Phosphatidylcholines in PA-exposed cells
fold change under PA (normalised to gCTR)
gRNF145
gADIPOR2
12:0,18:1
14:0,18:1
16:0,16:0
16:0,18:1
18:0,18:1
18:1,18:1
20:0,18:1

Figure 5.

membrane rigidity caused by the accumulation of these lipids in the membrane.

RNF145 regulates the lipid homeostatic response to palmitic acid

The reduction of DPPC in RNF145-deficient cells suggested that constitutively high ADIPOR2 levels induce cyto-protective changes to membrane lipid composition that increase membrane fluidity, and the converse occurs upon ADIPOR2 loss. To test this idea, we monitored the diffusion rate of a fluorophore-conjugated C12:0 lipid using fluorescent recovery after photobleaching (FRAP) analysis. Under PA-rich conditions, depletion of RNF145 decreased the average recovery time ($t_{1/2}$ = 1.75 s, SEM ± 0.12 s) in ER membrane compartments as compared to wild-type cells ($t_{1/2}$ = 2.60 s, SEM ± 0.09 s). By contrast, ADIPOR2 depletion increased the average recovery time ($t_{1/2}$ = 3.26 s, SEM ± 0.1 s) (Fig 6A; Appendix Fig S2A for knockdown efficiencies). Cells depleted of both RNF145 and ADIPOR2 showed a recovery time ($t_{1/2}$ = 2.98 s, SEM ± 0.04 s) similar to ADIPOR2-depleted cells, demonstrating that the RNF145 effect is ADIPOR2-dependent (Fig 6A).

The ability of resident membrane proteins to diffuse throughout the ER network is necessary for the many functions of this organelle. The changes to ER membrane fluidity in ADIPOR2- and RNF145-depleted cells, as suggested by our FRAP data, raises the possibility that generalised diffusion of resident ER proteins might consequently be affected. This was assessed by single molecule tracking (SMT) of an ER-resident reporter containing a Sec61β transmembrane domain (TMD) fused to a Halo tag (Halo-Sec61β-TMD) and labelled with a photoactivatable fluorescent Halo ligand (paJF646). Stochastic and sparse photoactivation of the Halo ligand allowed us to observe Halo-Sec61β-TMD particles in a single molecule regime and track their diffusion throughout the tubular ER. Crucially, overexpressed Halo-Sec61β-TMD acts as a biophysical reporter where the extracted mean particle velocities correlate with membrane fluidity in the tubular ER. The instantaneous velocities of Halo-Sec61β-TMD were significantly increased in RNF145-deficient cells (17.09 ± 0.034 (SEM) μm/s vs. 15.42 ± 0.037 (SEM) μm/s in control cells) but decreased in ADIPOR2-depleted cells (14.29 ± 0.038 (SEM) μm/s) (Fig 6B(i/ii); Appendix Fig S2B for knockdown efficiencies). Similarly, the effective diffusion coefficients (D_{eff}) were increased in RNF145-depleted cells (0.42 $\mu m^2/s$ vs. 0.35 $\mu m^2/s$ in control cells) but decreased in cells lacking ADIPOR2 (0.32 $\mu m^2/s$) (Fig 6B(iii)). Thus, both RNF145 and ADIPOR2 contribute to the maintenance of membrane fluidity as monitored by both lipid and protein probes.

Discussion

The balance between saturated and unsaturated membrane lipids must be tightly regulated as it is a critical determinant of lipid packing and membrane fluidity. Excess accumulation of saturated FAs like PA is cytotoxic and results in lipid bilayer stress and induction of the unfolded protein response (Mota *et al*, 2016; Halbleib *et al*, 2017; Palomer *et al*, 2018). Imbalances in lipid homeostasis also contribute to the severity of conditions such as liver steatosis, beta cell failure, complications of obesity and diabetes (Ipsen *et al*, 2018; Lytrivi *et al*, 2020).

How mammalian cells achieve lipid homeostasis is not well understood and prompted us to identify proteins that are acutely responsive to dietary FAs as part of their involvement in membrane lipid homeostasis. We identified RNF145 as an E3 ligase that is exquisitely sensitive to changes in lipid composition. RNF145's auto-ubiquitination and degradation are potently inhibited by unsaturated FAs (oleic, arachidonic or eicosapentaenoic acids), allowing RNF145 to regulate its substrate, ADIPOR2. Thus, RNF145 adjusts the levels of the lipid hydrolase ADIPOR2 in a FA-sensitive manner. ADIPOR2, in turn, modulates lipid bilayer properties and the incorporation of PA (and other saturated FAs) into phospholipids to maintain membrane homeostasis and cell viability under lipotoxic conditions.

At least two interrelated mechanisms allow RNF145 to sense, and ADIPOR2 to respond to changes in lipid composition: (i) Increasing membrane lipid saturation promotes the auto-degradation of RNF145, releasing ADIPOR2 for lipid hydrolysis and restoration of the membrane lipid composition. (ii) Decreasing lipid saturation stabilises RNF145 and increases its lipid-sensitive binding, ubiquitination and degradation of ADIPOR2. In the absence of RNF145, ADIPOR2 appears insensitive to changes in membrane lipid composition and is therefore entirely dependent on regulation by RNF145. Thus, RNF145 "fine-tunes" ADIPOR2 levels to meet the cellular requirement for membrane lipid hydrolysis under acute dietary FA fluctuations.

Sensing changes in membrane lipid composition provides a substantial challenge as lipids, the substrates to be regulated, are themselves embedded within the membrane bilayer. Integral membrane proteins are ideally suited to constantly monitor physicochemical membrane properties and communicate with downstream effectors via a conformational relay system. RNF145 appears particularly sensitive to FA saturation rather than chain length and may respond to global changes in membrane properties (i.e. membrane fluidity/viscosity and thickness) instead of individual lipid species. Exactly

Figure 6. Membrane fluidity is inversely affected in RNF145 and ADIPOR2 knockout cells.

A Fluorescence recovery after photobleaching (FRAP) in HEK-293T cells depleted of RNF145 (gRNF145), ADIPOR2 (gADIPOR2) or B2M (gCTR), and treated with PA (150 μM, 4 h). Knockout efficiencies are shown in Appendix Fig S2A. Mean of $n \geq 3$ (biological replicates) ± SD are shown. Boxplots represent the median, first and third quantiles. Upper and lower whiskers extend to values up to 1.5-fold interquartile range. Statistical significance was calculated using one-way ANOVA. *$q \leq 0.05$, ***$q \leq 0.001$, ****$q \leq 0.0001$.

B Single-molecule tracking (SMT) of Halo-Sec61β-TMD in U2-OS cells depleted of RNF145 (gRNF145), ADIPOR2 (gADIPOR2), or B2M (gCTR) and treated with PA (150 μM, 4 h). Representative SMT tracks are shown colour-coded by their mean track velocities (i). Mean instantaneous velocity (V_i) distributions and effective diffusion coefficients per cell (D_{eff}) across 3 independent experiments are shown in (ii) and (iii), respectively. The mean instantaneous velocities for gCTR (green), gRNF145 (blue) and gADIPOR2 (red) are indicated by vertical lines (ii). The total analysed cell numbers and tracks were: 20 cells (14,153 tracks) for gCTR, 26 cells (18,411 tracks) for gRNF145, 23 cells (14,806 tracks) for gADIPOR2. Statistical significance was tested using one-way ANOVA analysis with Holm-Sidak's (V_i data) or Tukey's multiple comparison test (D_{eff} data). Statistically significant differences are shown (*$q \leq 0.05$, ***$q \leq 0.001$). Knockout efficiencies are shown in Appendix Fig S2B. N = 3 biological replicates.

© 2022 The Authors

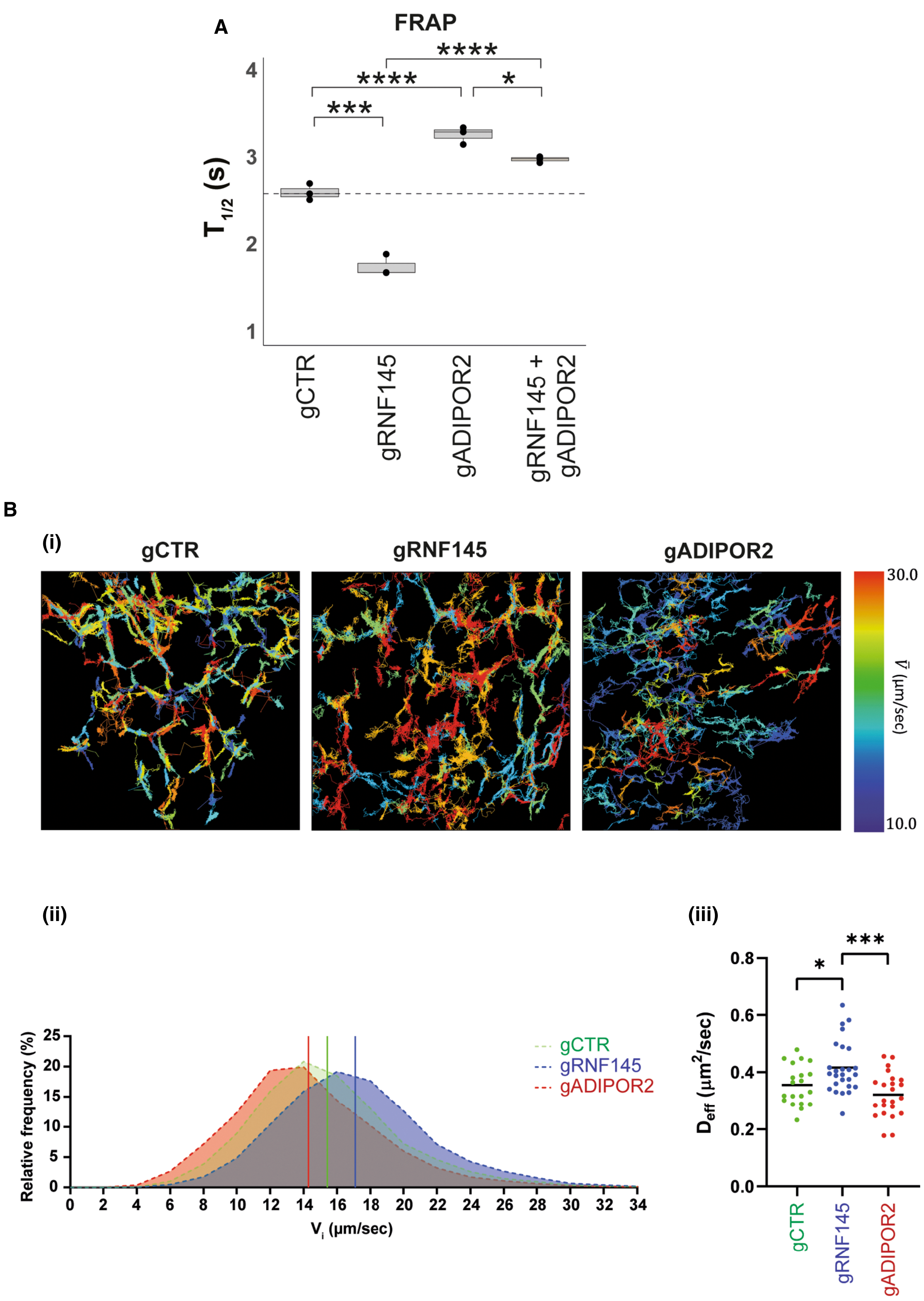
A
FRAP
****
****
*
***
$T_{1/2}$ (s)
4
3
2
1
gCTR
gRNF145
gADIPOR2
gRNF145 + gADIPOR2
B
(i)
gCTR
gRNF145
gADIPOR2
30.0
$\bar{V}$ (µm/sec)
10.0
(ii)
Relative frequency (%)
V_i (µm/sec)
gCTR
gRNF145
gADIPOR2
(iii)
*
***
D_{eff} (µm^2/sec)
gCTR
gRNF145
gADIPOR2

Figure 6.

how RNF145 can directly sense membrane properties through its extensive transmembrane domains is an exciting avenue of future research.

The role of ERAD proteins in lipid homeostasis is best understood in yeast, where the ER-resident transcription factors Mga2 and Spt23 regulate expression of the yeast acyl-CoA desaturase (*OLE1*) to enable fatty acid desaturation (Covino *et al*, 2016; Ballweg *et al*, 2020). Homodimeric, membrane-bound Mga2 and Spt23 are proteolytically activated in an FA-dependent manner by the combined action of the cytosolic E3 ligase Rsp5 and the yeast ERAD machinery (Hoppe *et al*, 2000; Rape *et al*, 2001; Surma *et al*, 2013; Ballweg & Ernst, 2017; Ballweg *et al*, 2020).

Mga2 senses the lipid-packing density in the membrane, rather than membrane fluidity itself. Although mammals lack orthologues of the yeast Mga2 and Spt23 membrane sensors, the increased complexity of membrane lipid composition suggests that mammalian cells are likely to have evolved additional analogous lipid sense-and-response modules. These would register distinct lipid species and membrane parameters to initiate an integrated cellular response. The sensing modalities are likely to differ, depending on which membrane properties are being monitored. Nevertheless, they seem to utilise a shared subset of ERAD components. We observed the involvement of VCP and UBXD8 in the FA-dependent (and steady-state) degradation of RNF145/ADIPOR2, similar to the role of Ubx2 in Mga2/Spt23 processing.

Like Mga2, the regulated turnover of ADIPOR2 by RNF145 is contingent on a lipid environment that changes steady-state membrane properties. It will be interesting to determine how RNF145 senses changes in membrane lipid properties. The RNF145/ADIPOR2 sense-and-response module seems distinct from that of Rsp5/Mga2 since RNF145 is itself an integral membrane protein and its auto-ubiquitination and turnover have an important role in the modulation of ADIPOR2.

Reminiscent of the OLE1 transcription pathway, but in response to cholesterol, the ER-resident SREBP cleavage-activating chaperone (SCAP), in complex with the SREBP2 transcription factor, couples membrane cholesterol sensing to cellular cholesterol demand. SCAP senses excess sterols via a loop in its sterol-sensing domain (SSD). Sterol binding induces a conformation which sequesters the SCAP-SREBP2 complex in an inactive state with INSIG1/2 and thereby suppresses transcription of SREBP2 target genes (Brown *et al*, 2002, 2018; Radhakrishnan *et al*, 2004).

HMG-CoA reductase (HMGCR) controls the rate-limiting step of the cholesterol *de novo* biosynthesis pathway. In excess sterols, RNF145 and gp78 are recruited to, ubiquitinate and promote the degradation of HMGCR, via the INSIG proteins. The involvement of RNF145 in regulating both HMGCR and ADIPOR2 might reflect a close co-ordination between the regulation of FAs and sterols. Our current data suggest that although RNF145 uses its transmembrane region to interact with ADIPOR2, INSIGs are not required for ADIPOR2 regulation (Fig EV3E). RNF145 may therefore integrate sterol and FA information differently. It is interesting to note that the "INSIG-dependent" regulation of HMGCR is shared with another E3 ligase, gp78, which can compensate for the loss of RNF145. Conversely, gp78 does not play a major role in ADIPOR2 regulation and RNF145 seems the only E3 ligase sufficient and necessary for ADIPOR2 turnover in the cell lines tested.

An RNF145-sensed change in membrane lipid composition regulates its own turnover and governs the interaction and degradation of ADIPOR2 by RNF145. ADIPOR2 therefore appears to be the passive player in this interaction, responding to the demands of RNF145. Structural analysis of the seven transmembrane domains of ADIPOR2 revealed a large internal cavity containing an OA molecule with solvent-accessible openings to the cytoplasm and lipid bilayer (Vasiliauskaité-Brooks *et al*, 2017). An alternative, but not mutually exclusive, model is that OA binding to ADIPOR2 induces a conformational change within its TMD region which promotes ADIPOR2 interaction with and subsequent ubiquitination by RNF145.

Our quantitative lipidomics analysis provides further insights into how ADIPOR2 buffers increase lipid saturation. In tissues, the majority of imported PA is readily incorporated into complex lipids, giving rise to highly saturated phospholipid or ceramide species, which directly affect membrane properties (Miras *et al*, 1983; Bruce & Salter, 1996). Among these lipids, the highly rigidifying DPPC is commonly present at low amounts, but becomes a major phospholipid species with PA supplementation (Woldseth *et al*, 1993). In hepatocytes, the initially rapid *de novo* synthesis of DPPC is followed by its equally fast decline, likely due to lipid remodelling from an unknown protein(s), restoring optimal membrane lipid composition (Woldseth *et al*, 1993). The metabolic fates of PA in ADIPOR2 and RNF145 KO cells differ. In the absence of ADIPOR2, PA continues to accumulate in both DPPC and sphingolipids (long-chain saturated ceramide species), resulting in decreased membrane fluidity, decreased protein mobility, and a loss in cell viability due to PA toxicity. The converse occurs following RNF145 depletion (thus increasing ADIPOR2), which caused reduced DPPC accumulation and increased PA storage in less cytotoxic TAGs (Listenberger *et al*, 2003).

In vitro, ADIPOR2 can convert C18 ceramide into free FAs and sphingosine (Vasiliauskaité-Brooks *et al*, 2017), but its low ceramidase activity suggests other potential hydrolase functions. Our data therefore strongly implies that ADIPOR2 acts as a more general lipid hydrolase that predominantly targets the saturated FA tails of phospholipid species. Thus, ADIPOR2's hydrolytic activity would release PA from its glycerol backbone (for PC) and sphingosine (for ceramide). This suggests that after the initial conversion of PA to DPPC, ADIPOR2 catalyses the hydrolysis of PA from DPPC as membrane rigidity increases. Released PA can be modified (e.g. desaturated, elongated) and used for the biosynthesis of complex lipids (promoting membrane remodelling and membrane relaxation), stored in TAGs or beta-oxidised (for energy generation). An increase of TAGs and palmitoyl-carnitine in RNF145 KO cells supports this mechanism (Fig EV4B).

ADIPOR2's hydrolase activity therefore effectively removes saturated phospholipids, such as DPPC, from the membrane, enabling phospholipid remodelling (Kita *et al*, 2019), promoting membrane fluidity and restoring membrane lipid homeostasis. Our findings are consistent with a screen for suppressors of the ADIPOR2 orthologue Paq-r2 in *C. elegans*, which implicated multiple genes in the PC biosynthesis pathway, hinting at imbalances in PC metabolism in ADIPOR2-deficient cells (Svensk *et al*, 2013). Since RNF145 regulates ADIPOR2 levels in response to lipids, this E3 ligase is a master regulator of cellular lipid homeostasis and membrane fluidity.

 © 2022 The Authors

Materials and Methods

Plasmids and expression constructs

Single guide RNA (sgRNA) sequences were inserted into pSpCas9 (BB)-2A-Puro V1/2 (RRID: Addgene 48139 and 62988, kind gift of Dr. Feng Zhang) as described earlier (Ran *et al*, 2013). RNF145 expression plasmids were cloned as reported previously (Menzies *et al*, 2018). The coding sequences of ADIPOR2 and SCD1 were amplified from cDNA isolated from HEK-293T cells using primers encoding a C-terminal HA tag and cloned into pHRSIN-P_{SFFV}-GFP-P_{PGK}-Hygromycin^R (Demaison *et al*, 2002), replacing GFP with the transgene. ADIPOR2-HA-Clover or ADIPOR2-HA-Ruby2 was inserted into pHRSIN-P_{SFFV}-P_{PGK}-Hygromycin^R by Gibson assembly cloning. ADIPOR2-HA, lysine to arginine, and catalytically dead mutants, were made as gene blocks (IDT, Belgium) and inserted into pHRSIN-P_{SFFV}-P_{PGK}-Hygromycin^R in between the BamHI NotI cut sites. RNF145-V5 and mRNF145-V5 were previously described (Menzies *et al*, 2018) or were subcloned from RNF145-V5 and mutated to produce a lysine to arginine mutant that was then moved into pHRSIN-P_{SFFV}-P_{PGK}-Hygromycin^R. For Airyscan microscopy, RNF145 were subcloned from RNF145-V5 with addition of an HAx3 epitope tag, ADIPOR-S was subcloned from ADIPOR2-HA and the HA sequence was replaced with sequence for the S epitope tag. Both were inserted, via Bam HI and Not I restriction sites, into the multiple cloning site of pHRSIN-P_{SFFV}-P_{PGK}-Hygromycin^R (RNF145-HAx3 and ADIPOR2-S) or pHRSIN-P_{SFFV}-P_{PGK}-Blasticidin (ADIPOR2-S). For single molecule tracking experiments, mEmerald-Sec61β-TMD was previously described (RRID: Addgene 90992; Nixon-Abell *et al*, 2016) and Halo-Sec61β-TMD was generated by replacing mEmerald with the HaloTag core sequence using Gibson assembly (generated in the Blackstone lab (Harvard Medical School, US)). Plasmids used for lentivirus production were pCMV delta R8.2 (RRID: Addgene 12263) and pMDG.2 (RRID: Addgene 12259), purchased from Addgene. The UBXD8 (K167E, R168E, R171E, K239E, R241E, R242E) mutant (Kim *et al*, 2013) was a kind gift from Jin Ye's laboratory (University of Texas Southwestern Medical Center, US).

Compounds

Compounds used in this study were as follows: bovine serum albumin (Albumine Bovine/Fraction V, for Biochemistry, pH 7.0 Thermo Fisher, 10053863), cOmplete protease inhibitor (EDTA-free; Roche, 27368400), Crystal Violet (Sigma, C3886), cycloheximide (Sigma-Aldrich, C-7698), digitonin (Merck, 300410), Dulbecco's Modified Eagle's Medium high glucose (DMEM; Sigma-Aldrich, 6429-500 ml), EasyTag™ EXPRESS^{35}S Protein Labelling Mix (PerkinElmer, NEG772002MC), fatty-acid free BSA (Sigma, A6003), foetal calf serum (FCS; Life Technologies (catalogue no: 10270, lot: 42G4179K)), FuGene 6 (Promega, E2691), HA peptide (Sigma-Aldrich, I2149), hygromycin B (Invitrogen, 10687010), IgG Sepharose™ 6 Fast Flow (GE Healthcare, 17-0969-01), iodoacetamide (IAA; Sigma-Aldrich, I1149-5G), FAs: dodecanoic acid (Sigma-Aldrich, L4250), myristic acid (Sigma-Aldrich, M3128), palmitic acid (Sigma-Aldrich, P5585), pentadecanoic acid (Sigma-Aldrich, P1625), stearic acid (Sigma-Aldrich, S4751), arachidic acid (Sigma-Aldrich, A3631), oleic acid (Sigma-Aldrich, O1383), eicosapentaenoic acid (C20:5n-Sigma-Aldrich, E2011), arachidonic acid (Sigma, A3611); lipoprotein-deficient serum (Biosera, FB-1001L/100); [Methyl-14C] methylated protein molecular weight markers (PerkinElmer, NEC811001UC), (S)-MG132 (Cayman Chemicals, 10012628), MF-438 (Merck, 569406), NMS-873 (Selleckchem, S728501), Opti-MEM™ (Thermo Fisher Scientific, 11524456), penicillin–streptomycin (10,000 U/ml; Thermo Fisher Scientific, 15140122), phenylmethylsulfonyl fluoride (PMSF; Roche, 20039220), poly-L-lysine (P4707, Sigma-Aldrich), Precision Plus Protein™Dual Colour Standards (Bio-Rad, 161–0374), ProLong™ Glass Antifade Mountant (Thermo Fisher Scientific, P36984), DAPI (Cell Signaling Technology), Protein A-HRP (Southern Biotech, 7300-05), Protein A-Sepharose^R (Sigma-Aldrich, P3391-1.5G), Protein A magnetic beads (Thermo Fisher Scientific, 88846), anti-HA magnetic beads (Thermo Fisher Scientific, 88836), puromycin (Cayman Chemicals, 13884), TransIT-293 (Mirus, MIR2700), TransIT-HeLa Monster (Mirus, MIR 2905), V5 peptide (Sigma-Aldrich, V7754-4MG), HA peptide (Sigma-Aldrich, I2149), Human derived fibronectin (Sigma Aldrich, F2006). Antibiotics: puromycin (Cayman chemicals, 13887), hyrgomycin B (Thermo Fisher Scientific, 10687-010), blasticidin (Corning, 3513-03-9). For mass spectrometry experiments all chemicals and reagents listed were of AR grade or better and were purchased from Merck unless otherwise stated.

Antibodies

Specific antibodies for the following proteins were used for immunoblotting: Hrd1 (rabbit; RRID:AB_2199838 Abgent, AP2184a), STUB1 (rabbit; RRID:AB_303412 Abcam ab2917), TRC8 (rabbit; RRID:AB_2238721 Santa Cruz, sc-68373), gp78 (rabbit; RRID:AB_2226463, ProteinTech, 16675-1-AP), tubulin (mouse; RRID:AB 477593 Sigma, T9026), VCP (mouse; RRID:AB_298039 abcam, ab11433), β-actin (mouse; RRID:AB_476743 Sigma-Aldrich, A5316), RNF145 (rabbit; ProteinTech, 24524-I-AP), ADIPOR1 (rabbit; RRID:AB_2893422 Novus, NBP2-67631), ADIPOR2 (rabbit; RRID:AB_2222052 ProteinTech, 14361-1-AP), SCD1 (rabbit; RRID:AB_823634 Cell Signaling Technologies, #2438), ubiquitin (mouse; RRID:AB_2716558 Life Sensors, VU101), UBE2G2 (mouse; RRID:AB_1130984 Santa Cruz, sc-100613), UBXD8 (RRID:AB_2893425, Abcam, ab154064), S-tag (Rabbit; RRID:AB_2920662 Abcam, ab183674), V5 tag (mouse; RRID:AB_471093 Abcam ab27671), HA tag (rat; RRID:AB_390919 Roche, 11867423001), HA tag (mouse; RRID:AB_10691311, Cell Signalling Technologies #2367), HRP-conjugated goat anti-mouse, anti-rabbit and anti-rat (goat; RRID:AB_2307392, RRID:AB_2307391 and RRID:AB_2338133, Jackson ImmunoResearch), TrueBlot® Anti-Rabbit-HRP (RRID:AB_2610847, Rockland, 18-8816-31), TrueBlot® Anti-Mouse-HRP ULTRA (RRID:AB_2610849, Rockland, 18-8817-30). Alexa Fluor 488 Goat anti-mouse (goat; RRID:AB_2633275 Thermo Fisher Scientific, A32723), Alexa Fluor 555 Goat anti-rabbit (goat; RRID:AB_2633281 Thermo Fisher Scientific, A32732) were used as secondary antibodies for immunofluorescence microscopy. Alexa Fluor 647 (rabbit anti-mouse; Thermo Fisher Scientific) was used for cytometric analysis.

Cell culture

All cell lines were cultured in DMEM +10% FCS + penicillin/streptomycin (complete DMEM) at 37°C, 5% CO_2 unless otherwise stated.

© 2022 The Authors

HeLa cells (female, RRID:CVCL_0030) were obtained from ECACC, HEK-293T (female, RRID:CVCL_0063), HepG2 (male, RRID: CVCL_0027) and U2-OS (female, RRID:CVCL_0042) cells were sourced from ATCC Huh7 (male) and primary microvascular endothelial cells were a kind gift of James Nathan (University of Cambridge, UK). All cell lines tested mycoplasma negative (Lonza MycoAlert, LT07-118). Fatty acid treatments were performed by seeding cells at low densities (for exact numbers refer to specific assays), rinsing the cells with PBS and subsequent addition of pre-complexed fatty acids (or BSA as vehicle control) (see "Preparation of fatty acids") in FCS-free DMEM or complete DMEM (see "Cell viability assays"). Sterol depletion assays were performed as described previously (Menzies *et al*, 2018).

CRISPR/Cas9-mediated gene depletion

CRISPR/Cas9-mediated genomic editing was performed as described previously (Ran *et al*, 2013). Knockout/−down cell lines were generated by transient transfection with pSpCas9(BB)-2A-Puro (PX459) V2.0 (Addgene #62988; kind gift of Dr. Feng Zhang (Broad Institute, US)), containing a target-specific sgRNA sequence (listed in Table 1). Typically, a mixture of 3–4 different sgRNAs per target gene was used to generate pooled knockout populations (annotated by the prefix "g" (i.e. when a "g" suffix is used such as "gRNF145" to describe a cell line, this indicates a heterogeneous pool of cells, not a single-cell clone)), while single cell clones were established using one sgRNA per gene (resulting single cell clones are designated as "KO" or "Δ" throughout this document), unless stated otherwise. B2M depletion (typically referred to as "gCTR") served as a negative control and was performed as described above using a single specific and previously validated sgRNA vector (Menzies *et al*, 2018). Cell transfection with one or multiple sgRNA vectors was performed using TransIT-HeLa MONSTER (Mirus) for HeLa cells, TransIT-293 (Mirus) for HEK-293T cells, or FuGene 6 (Promega) for U2-OS and Huh7 cells. An overall amount of 1 μg DNA (i.e. in the case of co-transfecting four different sgRNA vectors: 0.25 μg per vector) was used per well of a 12-well plate for reverse-transfection of $5*10^5$ HEK-293T or $1.5*10^5$ HeLa cells according to the manufacturer's instructions. U2-OS cells were reverse-transfected in 6-well tissue culture plates ($4*10^5$ cells/well). Cells were cultured post-transfection for 24 h and resistant, sgRNA-containing cells, were selected with puromycin (2 μg/ml) for 72 h. Single-cell clones were generated from these knockout populations using limiting dilution or fluorescence-assisted single-cell sorting. To generate cell lines depleted of multiple genes, sgRNAs specific for several different genes were either concomitantly transfected or knockout/−down populations were depleted of additional gene(s) by sequential transfection and selection as described herein. Gene knockouts were validated by immunoblotting, immunoprecipitation and/or genomic sequencing.

Lentivirus production and transductions

Lentivirus was produced as described previously (Menzies *et al*, 2018). In short, HEK-293T cells were transfected with a lentiviral expression vector containing the gene of interest, and two packaging vectors (pCMVΔR8.91 and pMD.G) at a ratio of 1:0.65:0.35. Virus-containing media was collected 48 h *post* transfection, filtered (0.45 μm pore size) and used for the transduction of target cells. In general, between 50,000 and 100,000 cells were transduced in 12-well plates (M.O.I. ≤ 1), followed by selection of stable expressors with hygromycin B (200 μg/ml) or blasticidin (10 μg/ml).

Flow cytometry analysis

Cells were collected by trypsinisation and centrifugation, resuspended in cold PBS and fluorescent proteins measured on an LSR Fortessa (BD) flow cytometer. Untransfected cells were used as negative control. Data processing and visualisation were performed using the FlowJo software package.

Cell viability assays

HEK-293T cells were seeded in technical duplicate to poly-L lysine (PLL) coated 24-well plates (4,000 cells per well) 16–24 h prior to treatment. The medium was then replaced with 500 μl fatty acid-containing complete DMEM and left for 96 h. Cells were fixed in ice-cold methanol:acetone (1:1; 10 min) and stained with 0.1% (w/v) Crystal Violet (Sigma-Aldrich) in 10% ethanol (10 min at RT). Excess staining solution was removed by rising in ddH_2O and plates were air-dried. Images were acquired performing high-resolution tile scans of each individual well using an Olympus IX51 microscope with Olympus cellSens (Olympus life science solutions, EVIDENT). Quantification was performed by measuring the integrated pixel density of the crystal violet-stained surface area above background noise using ImageJ (Fiji).

Quantitative PCR

RNA was purified from cells using the RNeasy Plus Mini Kit (Qiagen, Venlo, Netherlands) followed by reverse transcription using SuperScript™ III reverse transcriptase (Invitrogen) and an Oligo(dT) 15 primer (Promega, C110A). Transcript abundance was measured in technical triplicate using SYBR® Green PCR Master Mix (Applied Biosystems) and a real-time PCR thermocycler (7500 Real Time PCR System, Applied Biosystems). The following primer pairs were used for target amplification: RNF145 fw: 5′-AATCGGGGCATGACAG AAGG-3′, RNF145 rev: 5′-AGCACGGAAGTGTTTCCACA-3′; ADIPOR2 fw: 5′- ATGGCCAGCCTCTACATCAC-3′, ADIPOR2 rev: 5′-GCCGATC ATGAAACGAAACT-3′ actin fw: 5′-CTGGGAGTGGGTGGAGGC-3′, actin rev: 5′-TCAACTGGTCTCAAGTCAGTG-3′. RNA quantification was performed using the ΔΔCT method. Actin transcript levels served for normalisation.

SDS-PAGE and immunoblotting

Cells collected in cold PBS were centrifuged (1,000 *g*, 4 min, 4°C), resuspended in lysis buffer (1% (w/v) digitonin, 1x cOmplete protease inhibitor, 0.5 mM PMSF, 10 mM IAA, 2 mM NEM, 10 mM TRIS, 150 mM NaCl, pH 7.4) and left on ice (40 min). The post-nuclear fraction was isolated by centrifugation (17,000 *g*, 15 min, 4°C) and protein concentrations were determined by Bradford assay. Sample concentrations were adjusted with lysis buffer and 6× Laemmli buffer +100 mM dithiothreitol (DTT) before heating at

 © 2022 The Authors

Table 1. sgRNA sequences.

sgRNA	Forward
B2M (control guide)	GGCCGAGATGTCTCGCTCCG
RNF145 sg1	TGTTAAATGTGGCCCTG
RNF145 sg2	TTGGATGTCCTGTACAGAT
RNF145 sg3	GGAATTCAGAAAAGAGCCAG
RNF145 sg4	GGCTGCAAAGGAGAAACTGG
UBE2G2 sg1	CATGGGCTACGAGAGCAGCG
UBE2G2 sg2	TTACCTGCTACAATTCCTTC
UBE2G2 sg3	AGAATTAACACTGAATCCTC
ADIPOR2 sg1	ACAGGAAGAATACACAACCT
ADIPOR2 sg2	CAGTCTGGTAGTACATCATG
ADIPOR2 sg3	ATGATGGGCTTGTAAGAGAG
ADIPOR2 sg4	CTCTTCGTGTACCATCCAGT
gp78 sg1	ACTGGGCCACATCGCGGGCC
gp78 sg2	TGAGGCCCGTGTAGGTGCGG
UBXD8 sg1	ATGAAACAATGTCCCCAACG
UBXD8 sg2	GAAGGATCGAAGGATGACTG
UBXD8 sg3	ACAGCATAACTGGAACATAG

50°C (15 min). Samples were separated by SDS-PAGE on 4–12% continuous gradient polyacrylamide Bis-Tris gels (Thermo Fisher Scientific, NP0322BOX) and MOPS SDS-running buffer (Thermo Fisher, NP0001) or tris-glycine single-percentage gels. For immunodetection, proteins were transferred to methanol-activated PVDF membranes (Merck, IPVH00010). Membranes were incubated in 5% milk + PBST (PBS + 0.2% (v/v) Tween-20) (1 h) before transfer into PBST +2% (w/v) BSA or 5% milk containing primary antibody and left under constant motion at 4°C overnight or, alternatively, at RT (1 h). Proteins from whole-cell lysate were detected using peroxidase (HRP)-conjugated secondary antibodies. Immunoprecipitated proteins were detected using TrueBlot® HRP-conjugated secondary antibodies (Rockland) or Protein A-conjugated HRP (for RNF145) or species-appropriate secondary antibodies conjugated to HRP (Jackson).

Immunoprecipitation

Cells seeded to 15 cm tissue culture plates ($1*10^7$ cells per plate) the previous day were washed with PBS and cultured in FCS-free DMEM ± BSA or OA (200 μM) or PA (200 μM) for 3 h. Where indicated, cells were concomitantly treated with NMS-873 (50 μM). Cells were collected in cold PBS, concentrated (1,000 *g*, 4°C, 4 min), and lysed in IP buffer 1 (1% (w/v) digitonin, 10 μM $ZnCl_2$, 1x cOmplete protease inhibitor, 0.5 mM PMSF, 10 mM IAA, 10 mM TRIS, 150 mM NaCl, pH 7.4), post-nuclear fractions obtained by centrifugation (17.000xg, 4°C, 15 min), adjusted to 0.5% (w/v) digitonin and pre-cleared for 1 h with protein A magnetic beads. Endogenous ADIPOR2 and V5-tagged RNF145 were immunoprecipitated for 3 h at 4°C under constant overhead rotation using anti-HA magnetic beads or protein A magnetic beads in combination with anti-V5 or anti-ADIPOR2 antibody. Beads were collected on a DynaMag-2 magnetic rack (Thermo fisher, 12321D), washed at 4°C for 5 min with IP buffer 2 (0.5% (w/v) digitonin, 10 μM $ZnCl_2$, 10 mM Tris, 150 mM NaCl, pH 7.4) and 4 × 5 min at 4°C with IP buffer 3 (0.1% (w/v) digitonin, 10 μM $ZnCl_2$, 10 mM Tris, 150 mM NaCl, pH 7.4). HA-tagged proteins were eluted twice using 2 × 20 μl HA peptide (5 mg/ml, Sigma-Aldrich, I2149) in PBS pH 7.4, 0.5% (w/v) digitonin, 2x cOmplete protease inhibitor, for 30 min at 37°C under constant agitation. V5-tagged proteins were eluted twice using 2 × 20 μl V5 peptide (1 mg/ml, Sigma-Aldrich, V7754) in PBS pH 7.4, 0.5% (w/v) digitonin, 2x cOmplete protease inhibitor, for 30 min at 37°C under constant agitation on an Eppendorf Thermomixer. For immunoblotting, eluted samples were adjusted with Laemmli buffer and denatured at 50°C (15 min).

Ubiquitination assays

HEK-293T cells were seeded to PLL-coated tissue culture dishes ($5*10^6$ per 15 cm dish) and the following day rinsed with PBS and incubated in DMEM + oleic acid (400 μM) or BSA (as vehicle control) ± MG132 (5 μg/ml) or DMSO (as MG132 vehicle control) for 6 h. Cells were collected in ice-cold PBS, centrifuged (1,000 *g*, 4°C, 4 min), and lysed for 30 min on ice in IP buffer 4 (1% (v/v) triton-X 100, 1x cOmplete protease inhibitor, 0.5 mM PMSF, 10 mM IAA, 10 mM TRIS, 150 mM NaCl, pH 7.4). Post-nuclear fractions were obtained by centrifugation (17,000 *g*, 4°C, 15 min). Protein concentration was determined by Bradforrd assay and 2.5 mg post-nuclear lysate per sample (2.5 mg/ml) was pre-cleared using a mixture of 15 μl mouse IgG sepharose and 15 μl Protein A 50% bead slurry for 1 h, 4°C, under overhead rotation. ADIPOR2 was immunoprecipitated for 16 h from the pre-cleared samples using 30 μl Protein A 50% bead slurry and 3 μl anti-ADIPOR2 antibody (ProteinTech, 14361-1-AP) per sample. Beads were collected by centrifugation, washed for 5 min with IP buffer 4 and 4 × 5 min with IP buffer 5 (0.1% (v/v) triton-X 100, 1x cOmplete protease inhibitor, 0.5 mM PMSF, 10 mM IAA, 10 mM TRIS, 150 mM NaCl, pH 7.4). Proteins were eluted in 50 μl 2x Laemmli buffer +100 mM DTT on an Eppendorf Thermomixer at 60°C (15 min, 650 rpm) and separated via SDS-PAGE. Ubiquitin was detected using an anti-ubiquitin antibody (LifeSensors, VU101) according to the manufacturer's instructions.

^{35}S-methionine/cysteine pulse-chase

1×10^7 cells were seeded to a 10 cm tissue culture dish 24 h prior to trypsinisation. Cells were washed with PBS, and incubated for 30 min at 37°C in methionine/cysteine-free DMEM (Sigma) containing 10% dialyzed FCS and 20 mM HEPES. Metabolic labelling was performed by adding ^{35}S-methionine/cysteine [Met/Cys, EXPRESS^{35}S Protein Labelling Mix (PerkinElmer) in methionine/cysteine-free DMEM (Sigma)], 10% dialyzed FCS, 20 mM HEPES for 10 min at 37°C. The labelling reaction was quenched by adding complete DMEM. $3*10^6$ cells per time point were collected by transfer into ice-cold PBS, and cell pellets resuspended in 500 μl lysis buffer (1% TX100, 1 μM $ZnCl_2$, TBS, pH 7.4) on ice. Post-nuclear, detergent-soluble fractions were isolated by centrifugation and pre-cleared using a mixture of protein A sepharose and IgG sepharose beads. Endogenous ADIPOR2 was immunoprecipitated for 16 h using 30 μl 50% protein A sepharose bead slurry and 3 μl anti-ADIPOR2 antibody under overhead rotation at 4°C. HA-tagged ADIPOR2 was enriched with 30 μl 50%

© 2022 The Authors

protein HA-sepharose bead slurry. Immunoprecipitated material was washed 5 times for 5 min with ice-cold lysis buffer and overhead rotation, eluted in 25 µl 2x Laemmli + 100 mM DTT, followed by heating at 65°C for 15 min. Radiolabelled proteins were separated by SDS-PAGE and quantified by phosphoimaging using ImageQuantTL (cytiva). Half-lives of ADIPOR2 were calculated by fitting a linear regression model to the log10-transformed band intensities.

Cycloheximide chase assays

HEK293T cells were seeded to 3.5 cm tissue culture dishes ($4*10^5$ cells per dish), rinsed the following day with PBS and pre-treated for 20 min with cycloheximide (CHX, 1 µg/ml) in the presence or absence of MG132 (10 µg/ml) (0 h time point). The medium was then replaced with complete DMEM + CHX (1 µg/ml) + PA (400 µM), OA (400 µM) or BSA and incubated for the indicated times at 37°C, 5% CO_2. Cells were collected by scraping in ice-cold PBS and processed as described above (see "SDS-PAGE and immunoblotting").

Preparation of fatty acids

Fatty acid stock solutions were prepared with minor modifications as described previously (Luo *et al*, 2012). In brief, fatty acids were complexed with bovine serum albumin (BSA) (Argos, 268131000) or fatty-acid free BSA (Sigma, A0281). To this end, a 200 mM fatty acid stock solution was prepared in ethanol, diluted to 4 mM in warm (37°C), sterile-filtered DMEM containing 10% BSA and mixed for 2–16 h (37°C).

Quantitative proteomics

Treatment and collection of cells

For quantitative proteomics in Fig 1A, HEK-293T cells were seeded to 6-well tissue culture dishes ($4*10^5$ cells/well, 3 wells per condition), rinsed with PBS the following day and grown in FCS-free DMEM + BSA or PA (400 µM) or OA (400 µM) for 6 h. Cells were rinsed and collected in cold PBS. Half of the sample was further processed for MS/MS, while the remainder was used for immunoblot analysis. For the experiment shown in Fig 1B, single HeLa cell knockout clones (ΔRNF145, 3 independent clones), or WT HeLa cells, were seeded to 10 cm tissue culture dishes (10^7 cells/dish) and mechanically collected in cold PBS the following day. Differential protein expression was determined by TMT-labelling and LC–MS.

Lysis and protein quantification

Cells were washed twice with cold PBS and harvested by scraping into a 50 ml falcon tube and spun at 400 *g* for 5 min at 4°C. Pellets were resuspended in 400 µl cold PBS and transfer to 0.65 ml Bioruptor tubes (Diagenode) and pelleted again. Excess PBS was removed before resuspending the cells in 50 µl resuspension buffer (76 mM HEPES pH 7.55, 6 mM $MgCl_2$, Benzonase (1,400 µ/ml) and 15 mM TCEP) by pipetting. 18.75 µl 20% LDS was then immediately added to the cell suspension using a low retention pipette tip (RPT, StarLab) and pipetted to mix. Nucleic acids were fragmented by sonication in a Bioruptor Pico sonicator (Diagenode) for 10 min, 30 s on/off at 4°C. Samples were then incubated for 15 min at 37° to complete reduction. Samples were alkylated by adding 6 µl of 187.5 mM MMTS (final 15 mM) and incubating at RT for 15 min. Each sample was then approximately 100 µl volume. 5 µl aliquots of each sample was taken were diluted 2× in water and compared to a standard curve of BSA in the same buffer using a reducing agent compatible BCA assay (Thermo Fisher Scientific). 25 µg of each sample was taken, and the volumes of each lysate equalised using resuspension buffer +5% LDS.

S-trap digestion

To each sample a 10% volume of 12% phosphoric acid is added to acidify samples to ~ pH 2, completing denaturation. 6x volumes of wash buffer (100 mM HEPES pH 7.1, 90% Methanol) was added and the resulting solution was loaded onto a µS-trap (Protifi) using a positive pressure manifold ((PPM), Tecan M10), not more than 150 µl of sample at a time (~ 80 PSI). In-house fabricated adaptors were used to permit the use of S-traps with the manifold. Samples were then washed 4x with 150 µl wash buffer. To remove any remaining wash buffer, S-traps were centrifuged at 4,000 *g* for 2 min. To each S-trap, 30 µl of digestion solution (50 mM HEPES pH 8, 0.1% Sodium Deoxycholate (SDC)) containing 1.25 µg Trypsin/lysC mix (Promega) was added. S-Traps were then loosely capped and placed in low adhesion 1.5 ml microfuge tubes in a ThermoMixer C (Eppendorf) with a heated lid and incubated at 37°C for 6 h. Where digestion was carried out overnight, the thermomixer was set to 4°C after 6 h. Peptides were recovered by adding 40 µl digestion buffer to each trap an incubating at RT for 15 min before slowly eluting with positive pressure (2–3 PSI). Traps were subsequently eluted with 40 µl 0.2% formic acid (FA) and 40 µl 0.2% FA, 50% Acetonitrile (ACN) in the same manner. Eluted samples were then dried in a vacuum centrifuge equipped with a cold trap. For AP-MS experiments sample preparation differed as follows: Eluates were either eluted with 5% SDS or adjusted to 5% SDS after elution, prior to loading onto S-Traps and washing. SDC was omitted from the digestion buffer and 250 ng of typsin/LysC mix was used.

TMT labelling and clean up

Samples were resuspended in 21 µl 100 mM TEAB pH 8.5. After cooling to room temperature, 0.2 mg TMT reagents (Thermo Fisher Scientific) were resuspended in 9 µl anhydrous ACN which was added to the respective samples and incubated at room temperature for 1 h. A 3 µl aliquot of each sample was taken and pooled to check TMT labelling efficiency and equality of loading by LC–MS. Samples were stored at −80°C in the interim. After checking each sample was at least 98% TMT labelled, total reporter ion intensities were used to normalise the pooling of the remaining samples such that the final pool should be as close to a 1:1 ratio of total peptide content between samples. This final pool was then dried in a vacuum centrifuge to evaporate the majority of ACN from labelling. The sample was acidified to a final 0.1% Trifluoracetic Acid (TFA) (~ 200 µl volume) and formic acid was added until the SDC visibly precipitated; 4 volumes of ethyl acetate were then added and the sample vortexed vigorously for 30s. Sample was then centrifuged at 15,000 *g* for 5 min at RT to effect phase separation. A gel loading pipette tip was used to withdraw the lower (aqueous) phase to a fresh low adhesion microfuge tube. If any obvious SDC contamination remained the two-phase extraction with ethyl acetate was repeated. The sample was then partially dried in a vacuum

 © 2022 The Authors

centrifuge and brought up to a final volume of 1 ml with 0.1% TFA. Formic acid was added until the pH was ≤2, confirmed by spotting onto pH paper. The sample was then cleaned up by SPE using a 50 mg tC18 SepPak cartridge (Waters) and a PPM. The cartridge was wetted with 1 ml 100% Methanol followed by 1 ml ACN, equilibrated with 1 ml 0.1% TFA and the sample loaded slowly. The sample was passed twice over the cartridge. The cartridge was washed 3x with 1 ml 0.1% TFA before eluting sequentially with 250 µl 40% ACN, 70% ACN and 80% ACN and dried in a vacuum centrifuge.

Basic pH reversed-phase fractionation

TMT labelled samples were resuspended in 40 µl 200 mM ammonium formate at pH 10 and transferred to a glass HPLC vial. BpH-RP fractionation was conducted on an Ultimate 3000 UHPLC system (Thermo Fisher Scientific) equipped with a 2.1 mm × 15 cm, 1.7 µ Kinetex EVO column (Phenomenex). Solvent A was 3% ACN, solvent B was 100% ACN, solvent C was 200 mM ammonium formate (pH 10). Throughout the analysis, solvent C was kept at a constant 10%. The flow rate was 500 µl/min and UV was monitored at 280 nm. Samples were loaded in 90% A for 10 min before a gradient elution of 0–10% B over 10 min (curve 3), 10–34% B over 21 min (curve 5), 34–50% B over 5 min (curve 5) followed by a 10 min wash with 90% B. 15 s (100 µl) fractions were collected throughout the run. Fractions containing peptide (as determined by A280) were recombined across the gradient to preserve orthogonality with online low pH RP separation. For example, fractions 1, 25, 49, 73, 97 were combined and dried in a vacuum centrifuge and stored at −20°C until LC–MS analysis. A total of 24 Fractions were generated in this manner.

Mass spectrometry

Samples were analysed on an Orbitrap Fusion instrument online with an Ultimate 3000 RSLC nano UHPLC system (Thermo Fisher Scientific). Samples were resuspended in 10 µl 5% DMSO/1% TFA. A 5 µl of each fraction was Injected for TMT experiments, the whole sample was injected for AP-MS experiments. Trapping solvent was 0.1% TFA, analytical solvent A was 0.1% formic acid, solvent B was ACN with 0.1% formic acid. Samples were loaded onto a trapping column (300 µm × 5 mm PepMap cartridge trap (Thermo Fisher Scientific)) at 10 µl/min for 5 min. Samples were then separated on a 75 cm × 75 µm i.d. A 2 µm particle size PepMap C18 column (Thermo Fisher Scientific). The gradient was 3–10% B over 10 min, 10–35% B over 155 min, 35–45% B over 9 min followed by a wash at 95% B for 5 min and re-equilibration at 3% B. Eluted peptides were introduced by electrospray to the MS by applying 2.1 kV to a stainless-steel emitter (5 cm × 30 µm (PepSep)). During the gradient elution, mass spectra were acquired with the parameters detailed in Appendix Supplementary Materials and Methods (A) using Tune v3.3 and Xcalibur v4.3 (Thermo Fisher Scientific).

Data processing

Data were processed with PeaksX+, v10.5 (Bioinfor). Processing parameters are shown in detail in the Appendix Supplementary Materials and Methods (B). Briefly, "*.raw" files were searched iteratively in three rounds, with unmatched DeNovo spectra (at 0.1% PSM FDR) from the previous search used as the input for the next one. The three iterations were as follows (1) Swissprot Human (27/03/2020) + common contaminants (2) The same databases as search 1 but permitting semi-specific cleavage (3) trEMBL Human (27/03/2020), with specific cleavage rules. Proteins were then quantified using the parameters outlined in Appendix Supplementary Materials and Methods (B). Identified proteins and their abundances were output to "*.csv", imported to R and submitted to statistical analysis using LIMMA, a moderated *t*-test available through the Bioconductor package (Bioconductor.org). LIMMA *P* values were corrected for multiple hypothesis testing using the Benjamini-Hochberg method to generate an FDR (*q* value) for each comparison.

Lipidomics

Cell preparation and treatments

HEK-293T depleted of B2M (gCTR), RNF145 (gRNF145) or ADIPOR2 (gADIPOR2) were generated as described in section "CRISPR/Cas9-mediated gene depletion". Cells were seeded to PLL-coated 10 cm tissue culture dishes ($3*10^6$ cells/dish) and, on the following day, rinsed with 10 ml PBS and incubated in FCS-free DMEM + BSA/PA (150 µM)/OA (400 µM) for 20 h. Cells were collected by rinsing four times in 10 ml PBS, followed by accutase treatment and further processed for lipidomic profiling.

Extraction of lipid metabolites

Lipids were extracted in chloroform:methanol (2:1) as described previously (Folch *et al*, 1957). All solvents used were of HPLC or LCMS grade. 1 ml of chloroform: methanol (2:1) was added to cell pellets in Eppendorf tubes (Star Labs), followed by vortexing and sonication for 5 min, addition of 400 µl ice-cold water per sample and an additional round of vortexing and sonication for 5 min. Samples were centrifuged (20,000 *g*, 5 min) and the organic (bottom layer) fraction transferred into glass vials (Chromacol) for the organic layers and kept on dry ice. The resulting layers were dried (as described below) and stored at −70°C prior to analysis.

LC–MS sample preparation

Cell lipid extracts were dried using a centrifugal evaporator (Savant, Thermo Fisher Scientific) and reconstituted in 500 µl chloroform: methanol (1:1), followed by dilution of 10 µl of the resulting solution into 90 µl 2-propanol:acetonitrile:water (2:1:1) containing a deuterated internal standard lipid mixture (2.5 µg/ml per lipid). The following internal standards (Avanti Lipids) were used: N-palmitoyl-d31-D-erythro-sphingosine, 1-palmitoyl-d31-2-oleoyl-sn-glycero-3-phosphate (sodium salt), 1-palmitoyl(d31)-2-oleyl-sn-glycero-3-phosphocholine, 1-palmitoyl(d31)-2-oleyl-sn-glycero-3-phosphoethanolamine, 1-palmitoyl-d31-2-oleoyl-sn-glycero-3-[phospho-rac-(1-glycerol)] (sodium salt), 1-palmitoyl-d31-2-oleoyl-sn-glycero-3-phosphoinositol (ammonium salt), 1,2-dimyristoyl-d54-sn-glycero-3[phospho-L-serine] (sodium salt), N-palmitoyl(d31)-d-erythro-sphingosylphosphorylcholine, 1,2-dipalmitoyl-d62-sn-glycero-3-[phospho-L-serine], cholesteryl-2,2,3,4,4,6-d6-octadecanoate, pentadecanoic-d29 acid, heptadecanoic-d33-acid, eicosanoic-d39 acid, tetradecylphosphocholine-d42, glyceryl tri (pentadecanoate-d29), glyceryl-tri(hexadecanoate-d31) and glyceryl tri(octadecanoate-d35). All internal standards were prepared from primary stocks at 1 mg/ml in chloroform:methanol (1:1) or methanol where appropriate. Saponified lipid extracts for total fatty acid analysis were directly reconstituted in the above internal

© 2022 The Authors

standard mix without dilution. The samples were then vortexed, transferred into a 300 µl glass vial (Chromacol) and sealed (Agilent).

LC–MS analysis of lipids

For LC–MS analysis, a Q Exactive Plus orbitrap coupled to a Vanquish Horizon ultrahigh performance liquid chromatography system was used. LC–MS analysis was performed using a Waters CSH C18 column (50 × 2.1 mm, 2.0 µm). The mobile phase A consisted of acetonitrile:water (6:4) + ammonium formate (10 mM) and mobile phase B of 2-propanol: acetonitrile (9:1) + ammonium formate (10 mM). For gradient elution of compounds, the mobile phase B started at 40% with an increase to 43% at 1 min, 50% at 1.1 min, followed by a linear gradient to 54% B over 4.9 min and a further increase to 70% over 0.1 min followed by a linear gradient to 99% B over 2.9 min with re-equilibration for 1.5 min, resulting in a total run time of 10.5 min. The flow rate was 0.4 ml/min and the injection volume 5 µl. The needle wash used was 2-propanol: acetonitrile:water (2:1:1) + formic acid (0.1%).

Source parameters used for the mass spectrometer were a vapouriser temperature of 425°C and an ion transfer tube temperature of 300°C with an ion spray voltage of 3.5 kV (2.5 kV for negative ion mode) and a sheath gas of 50, auxiliary gas of 13 and a sweep gas of 3 arbitrary units with an S-lens RF (radio frequency) of 70%. For MS analysis, a full scan of 150–2,000 m/z was used for both positive and negative ion mode at a resolution of 30,000 ppm with fast positive–negative switching. Unknown compound identification was assisted using targeted higher energy collisional dissociation fragmentation (HCD) at a normalised collision energy of 30%. All solvents and additives were obtained from ThermoFisher Scientific or Merck and of LCMS or Optima grade.

LC–MS data processing

All data were acquired using Xcalibur (v. 4.1, ThermoFisher Scientific). Targeted processing was performed by Xcalibur and unbiased analysis using Compound Discoverer (v. 3.1, ThermoFisher Scientific). For unbiased analysis, metabolites were verified using the high resolution m/z LIPID MAPS Lipidomics Gateway database (https://lipidmaps.org/) with reference to fragmentation data where appropriate. Where necessary, peak areas corresponding to metabolite levels were normalised to internal standards or total ion content and presented as relative areas. All data were collated and targeted quantitation was processed using Excel (2016 version, Microsoft).

Files associated with the raw data for Figs 5D and E, and EV4 are supplied as Dataset files (Dataset EV2).

Single-molecule tracking

U2-OS cells were depleted of B2M, RNF145, or ADIPOR2 as described above (see "CRISPR/Cas9-mediated gene depletion") and transfected with a construct expressing the minimal transmembrane domain (TMD) sequence of Sec61β fused to mEmerald (mEm-Sec61β-TMD) as described previously (Nixon-Abell *et al*, 2016). Cells were seeded at ~40% confluency onto 400 µg/ml Matrigel (Corning)-coated, high-precision 1.5 mm coverslips (MatTek), followed by transfection using FuGene HD (Promega) at a 3:1 FuGENE (µl): DNA (µg) ratio 24 h after seeding. 0.75 µg mEm-Sec61β-TMD and 0.25 µg of Halo-Sec61β-TMD DNA were used per 35 mm chamber. Cells were treated with 150 µM PA and imaged 18–24 h post transfection.

For single-molecule tracking, Halo-Sec61β-TMD was labelled with the photoactivatable paJF646 ligand in optiMEM (Gibco) at a final concentration of 500 nM as described previously (Grimm *et al*, 2015). Sample imaging was performed at 5% CO2 and 37°C in a humidified stage-top incubator (PeCon GmbH), with a Definite Focus 2 device (Carl Zeiss) for thermal drift correction and ZEN 3.0 SR software (Carl Zeiss) for data acquisition.

Cells were imaged on a Zeiss Elyra 7 (Carl Zeiss) in HiLO illumination mode using simultaneous excitation from a 500 mW 488 nm OPSL and 500 mW 642 nm diode laser. Laser power density measurements at the sample site were calculated to be 96 W/cm^2 (643 nm laser) and 5.14 W/cm^2 (488 nm laser). Stochastic photoconversion of the Halo-bound paJF646 ligand was induced using a 50 mW 405 nm laser used at variable low intensities. The resulting fluorescence was collected (alpha Plan-Apochromat 63x/1.46 Oil Corr M27 TIRF objective), directed through a quad beam splitter (LBF: 405/488/561/642 nm) into a DuoLink SR fitted with an SBS LP 560 splitter and detected by two 1,280 × 1,280 pixel (size: 6.5 µm × 6.5 µm) pco.edge 4.2 sCMOS cameras (PCO). Data acquisition at the maximum achievable framerate (167 Hz) was achieved using a 128 × 128 pixel central subarray of the camera chip.

By using the mEmerald-Sec61β-TMD fluorescence, we selected subcellular regions containing peripheral tubular ER and converted the resulting localisations collected from the paJF646 labelled Halo Sec61β-TMD to tracks using the Trackmate FIJI plugin (NIH) (Tinevez *et al*, 2017). For particle detection, a Laplacian of Gaussian (LoG) filter was applied to the image series with a spot diameter of 500 nm and a threshold value of 3.0–7.0. Sub-pixel localisation was achieved using a quadratic fitting regime. A Linear Assignment Problem (LAP) mathematical framework adapted from (Jaqaman *et al*, 2008), was used to generate tracks of a linking max distance and gap-closing max distance of 800 nm, and a gap-closing max frame gap of 5 frames. Only tracks comprised of ≥ 50 localisations were considered for analysis.

The motion of individual molecules was analysed by extracting the distance between subsequent localisations in each trajectory of Halo Sec61β-TMD. When divided by the time interval between frames, this yielded the instantaneous speed (V). The mean effective diffusion coefficient in two dimensions (D_{eff}) was estimated from the size of single steps.

Step size or V_i were extracted from each track and plotted as a frequency histogram, with D_{eff} calculated from all tracks for each cell according to $D_{eff} = \langle x^2 \rangle / 4t$, using the mean squared displacement (MSD – $\langle x^2 \rangle$) and time interval (t). We note that the assumption of linear 2D diffusion does not account for the effects of structural confinement and flow that are relevant to accurately estimate reliable diffusion coefficients throughout the ER network. However, since we have no evidence to suggest that these variables are significantly altered between the various genotypes interrogated here, the reported D_{eff} values in this manuscript offer important comparative insights. Indeed, changes in protein dynamics reported thus likely reflect global changes in the material properties of the membrane.

Indirect immunofluorescence Airyscan microscopy

HEK-293T cells (2 × 10^5/well) were infected with lentivirus encoding ADIPOR2-S (pHRSIN-P_{SFFV}-ADIPOR2-S-P_{PGK}-Blast) or RNF145-HAx3 (pHRSIN-P_{SFFV}-RNF145-HAx3-P_{PGK}-Hygro) and ADIPOR2-S

 © 2022 The Authors

encoding lentivirus overnight before selection with antibiotics (hygromycin B or blasticidin). After recovery, cells were transfected using 0.25 μg/μl mEmerald-Farnesyl-5 (Addgene #54093) or an N-terminal PDI-derived signal peptide and C-terminal KDEL-containing mEmerald plasmid at 1 μg/μl with TransIt-293 (Mirus). After 16 h, cells were washed and plated on Fibronectin-coated coverslips at 2.5×10^4 cells per well. Coverslips were coated with human-derived fibronectin (Sigma) 50 μg/μl diluted in PBS from a stock of 1 mg/ml fibronectin in PBS. Coverslips were incubated with 50 μg/μl fibronectin for 1 h at 37°C before washing in DMEM (Sigma) and blocked for 1 h in DMEM-containing FCS at 37°C. After plating, cells were left to attach overnight, then directly fixed in 4% PFA in 1xPBS diluted from EM grade 10% PFA stock (Polyscience) and fixed for 15 min at 37°C. Samples were washed 3 times with Tris-buffered saline pH 7.4 (TBS). Cell membranes were permeabilised in 0.1% triton-X-100 (Sigma) in TBS, then blocked in 2% BSA/TBS (w/v; Thermo Fisher Scientific) in TBS for 20 min. Cells were stained with primary antibody diluted in 2% BSA/TBS (1 h), washed with TBS, followed by staining with secondary antibody in 2% BSA/TBS with DAPI (30 min) and three washing steps in PBS, then by distilled H_2O and mounted on slides overnight using ProLong™ Glass Antifade mountant (Thermo Fisher Scientific, P36980). Images were taken on a Zeiss 980 microscope fitted with an AIRYSCAN2 module and solid state lasers 405, 488, 561 and 630 nm (Carl Zeiss, Germany). Images were acquired by capturing each channel sequentially at full resolution and maximum scan speed before processing with SR- auto processing using the Zen blue software (Carl Zeiss).

Statistics

If not stated otherwise, mean values ± standard deviation (SD) are shown. Boxplots represent the median, first and third quantiles. Upper and lower whiskers extend to values up to 1.5-fold interquartile range. Data points beyond the whiskers were plotted individually. Statistical significance was calculated using the unpaired Student's *t*-test with a significance threshold of $p = 0.05$, or Wilcoxon Rank Sum Test with Benjamini-Hochberg multiple testing correction and an adjusted significance threshold of $q = 0.05$. Ratiometric data were log10-transformed prior to statistical testing. For the lipidomics and colony formation assays, a one-way ANOVA was performed with post-hoc Benjamini-Hochberg multiple testing correction. For proteomic data, q values were calculated using Benjamini-Hochberg multiple testing correction. *N* designates the number of independently performed biological repeats, typically on different days, except for Fig 1B, where *n* represents the number of different knockout clones per gene. For the single-molecule tracking experiments, distribution of the data was assessed by D'Agostino-Pearson and Kolmogorov–Smirnov tests. Blinding was not performed on individual experiments.

Data availability

The proteomics data produced in this study have been deposited on the PRIDE repository (Perez-Riverol *et al*, 2022, https://www.ebi.ac.uk/pride/) under the identifier PXD034835. An abridged version is available in the Dataset EV1. Lipidomics data are supplied with this submission as supplement file Dataset EV2.

Resource availability

All unique/stable reagents generated in this study are available from the Lead Contact with a completed Materials Transfer Agreement.

Expanded View for this article is available online.

Acknowledgements
We are grateful to the Cell phenotyping hub and imaging facility support staff at the CITIID. This work was supported by the Wellcome Trust, through a Principal Research Fellowship to PJL (210688/Z/18/Z) and the NIHR Cambridge Biomedical Research Centre. AK was supported by Wellcome trust investigator awards 106260/Z/14/Z and 222497/Z/21/Z. Work by JNA was supported by a Henry Wellcome Fellowship (218651/Z/19/Z). PHStGH was supported by the Canadian Institutes of Health Research via a Foundation Grant and Canadian Consortium on Neurodegeneration in Aging Grant, Alzheimer Society of Ontario, a Wellcome Trust Collaborative Award (203249/Z/16/Z) and a US Alzheimer Society Zenith Grant (ZEN-18-529769). We would like to thank Brian Ortmann and James Nathan (CITIID Cambridge) for gifting the MVECs, the Lavis group (HHMI Janelia) for providing the photoactivatable paJF646 ligand, the Blackstone group (Harvard Medical School) for sharing the HaloTag plasmid, Jin Ye (University of Texas Southwestern Medical Center) for kindly providing the mutant UBXD8 plasmid and David Ron (CIMR Cambridge) and Manu Hedge (MRC Laboratory Cambridge) for advice.

Author contributions
Norbert Volkmar: Conceptualization; resources; data curation; formal analysis; validation; investigation; visualization; methodology; writing – original draft; writing – review and editing. **Christian M Gawden-Bone:** Resources; data curation; formal analysis; validation; investigation; methodology; writing – original draft; writing – review and editing. **James C Williamson:** Resources; data curation; formal analysis. **Jonathon Nixon-Abell:** Data curation; formal analysis; investigation; methodology; writing – original draft; writing – review and editing. **James West:** Resources; data curation; formal analysis; investigation. **Peter H St George-Hyslop:** Resources; supervision. **Arthur Kaser:** Resources; supervision. **Paul J Lehner:** Conceptualization; formal analysis; supervision; funding acquisition; investigation; writing – original draft; writing – review and editing.

In addition to the CRediT author contributions listed above, the contributions in detail are:

Conceptualisation: N.V., P.J.L. Methodology: N.V., C.G.B., J.C.W., J.A.W., J.N.A., P.J.L. Investigation: N.V., C.G.B., J.C.W., J.A.W., J.N.A. Validation: N.V., C.G.B. Formal analysis: N.V. Visualisation: N.V., S.F.B., M.J.M., J.L.S., E.H. Data Curation: J.A.W., J.C.W., P.J.L. Writing—original draft: N.V., C.G.B., P.J.L. Writing—review & editing: N.V., C.G.B., J.C.W., J.A.W., J.N.A., P.J.L. Supervision: P.J.L. Project administration: N.V., C.G.B. Funding acquisition: P.J.L. Additional resources and material: P.S.G.H., A.K.

Disclosure and competing interests statement
The authors declare that they have no conflicts interest.

References

Alexandru G, Graumann J, Smith GT, Kolawa NJ, Fang R, Deshaies RJ (2008) UBXD7 binds multiple ubiquitin ligases and implicates p97 in HIF1α turnover. *Cell* 134: 804 – 816

© 2022 The Authors

ALJohani AM, Syed DN, Ntambi JM (2017) Insights into Stearoyl-CoA Desaturase-1 regulation of systemic metabolism. *Trends Endocrinol Metab* 28: 831 – 842

Antonny B, Vanni S, Shindou H, Ferreira T (2015) From zero to six double bonds: phospholipid unsaturation and organelle function. *Trends Cell Biol* 25: 427 – 436

Ballweg S, Ernst R (2017) Control of membrane fluidity: the OLE pathway in focus. *Biol Chem* 398: 215 – 228

Ballweg S, Sezgin E, Doktorova M, Covino R, Reinhard J, Wunnicke D, Hänelt I, Levental I, Hummer G, Ernst R (2020) Regulation of lipid saturation without sensing membrane fluidity. *Nat Commun* 11: 756

Bjursell M, Ahnmark A, Bohlooly-Y M, William-Olsson L, Rhedin M, Peng XR, Ploj K, Gerdin AK, Arnerup G, Elmgren A *et al* (2007) Opposing effects of adiponectin receptors 1 and 2 on energy metabolism. *Diabetes* 56: 583 – 593

Bodhicharla R, Devkota R, Ruiz M, Pilon M (2018) Membrane fluidity is regulated cell nonautonomously by Caenorhabditis elegans PAQR-2 and its mammalian homolog AdipoR2. *Genetics* 210: 189 – 201

Borradaile NM, Han X, Harp JD, Gale SE, Ory DS, Schaffer JE (2006) Disruption of endoplasmic reticulum structure and integrity in lipotoxic cell death. *J Lipid Res* 47: 2726 – 2737

Brown AJ, Sun L, Feramisco JD, Brown MS, Goldstein JL (2002) Cholesterol addition to ER membranes alters conformation of SCAP, the SREBP escort protein that regulates cholesterol metabolism. *Mol Cell* 10: 237 – 245

Brown MS, Radhakrishnan A, Goldstein JL (2018) Retrospective on cholesterol homeostasis: the central role of Scap. *Annu Rev Biochem* 87: 783 – 807

Bruce JS, Salter AM (1996) Metabolic fate of oleic acid, palmitic acid and stearic acid in cultured hamster hepatocytes. *Biochem J* 316: 847 – 852

Chen Y-L, Tao J, Zhao P-J, Tang W, Xu J-P, Zhang K-Q, Zou C-G (2019) Adiponectin receptor PAQR-2 signaling senses low temperature to promote *C. elegans* longevity by regulating autophagy. *Nat Commun* 10: 2602

Claypool SM (2009) Cardiolipin, a critical determinant of mitochondrial carrier protein assembly and function. *Biochim Biophys Acta* 1788: 2059 – 2068

Covino R, Ballweg S, Stordeur C, Michaelis JB, Puth K, Wernig F, Bahrami A, Ernst AM, Hummer G, Ernst R (2016) A eukaryotic sensor for membrane lipid saturation. *Mol Cell* 63: 49 – 59

Cunha DA, Hekerman P, Ladriere L, Bazarra-Castro A, Ortis F, Wakeham MC, Moore F, Rasschaert J, Cardozo AK, Bellomo E *et al* (2008) Initiation and execution of lipotoxic ER stress in pancreatic-cells. *J Cell Sci* 121: 2308 – 2318

Demaison C, Parsley K, Brouns G, Scherr M, Battmer K, Kinnon C, Grez M, Thrasher AJ (2002) High-level transduction and gene expression in hematopoietic repopulating cells using a human Imunodeficiency virus type 1-based Lentiviral vector containing an internal spleen focus forming virus promoter. *Hum Gene Ther* 13: 803 – 813

Dowhan W, Vitrac H, Bogdanov M (2019) Lipid-assisted membrane protein folding and Topogenesis. *Protein J* 38: 274 – 288

Ernst R, Ejsing CS, Antonny B (2016) Homeoviscous adaptation and the regulation of membrane lipids. *J Mol Biol* 428: 4776 – 4791

Flowers MT, Ntambi JM (2008) Role of stearoyl-coenzyme a desaturase in regulating lipid metabolism. *Curr Opin Lipidol* 19: 248 – 256

Folch J, Lees M, Sloane SG (1957) A simple method for the isolation and purification of Total Lipides from animal tissues. *J Biol Chem* 226: 497 – 509

Gawden-Bone CM, Griffiths GM (2019) Phospholipids: pulling back the actin curtain for granule delivery to the immune synapse. *Front Immunol* 10: 700

Grimm JB, English BP, Chen J, Slaughter JP, Zhang Z, Revyakin A, Patel R, Macklin JJ, Normanno D, Singer RH *et al* (2015) A general method to improve fluorophores for live-cell and single-molecule microscopy. *Nat Methods* 12: 244 – 250

Halbleib K, Pesek K, Covino R, Hofbauer HF, Wunnicke D, Hänelt I, Hummer G, Ernst R (2017) Activation of the unfolded protein response by lipid bilayer stress. *Mol Cell* 67: 673 – 684.e8

Harayama T, Riezman H (2018) Understanding the diversity of membrane lipid composition. *Nat Rev Mol Cell Biol* 19: 281 – 296

Holland WL, Xia JY, Johnson JA, Sun K, Pearson MJ, Sharma AX, Quittner-Strom E, Tippetts TS, Gordillo R, Scherer PE (2017) Inducible overexpression of adiponectin receptors highlight the roles of adiponectin-induced ceramidase signaling in lipid and glucose homeostasis. *Mol Metab* 6: 267 – 275

Holthuis JCM, Menon AK (2014) Lipid landscapes and pipelines in membrane homeostasis. *Nature* 510: 48 – 57

Hoppe T, Matuschewski K, Rape M, Schlenker S, Ulrich HD, Jentsch S (2000) Activation of a membrane-bound transcription factor by regulated ubiquitin/proteasome-dependent processing. *Cell* 102: 577 – 586

Inukai K, Nakashima Y, Watanabe M, Takata N, Sawa T, Kurihara S, Awata T, Katayama S (2005) Regulation of adiponectin receptor gene expression in diabetic mice. *Am J Physiol Endocrinol Metab* 288: E876 – E882

Ipsen DH, Lykkesfeldt J, Tveden-Nyborg P (2018) Molecular mechanisms of hepatic lipid accumulation in non-alcoholic fatty liver disease. *Cell Mol Life Sci* 75: 3313 – 3327

Jain IH, Calvo SE, Markhard AL, Skinner OS, To T, Ast T, Mootha VK (2020) Genetic screen for cell fitness in high or low oxygen highlights mitochondrial and lipid metabolism. *Cell* 181: 716 – 727.e11

Jaqaman K, Loerke D, Mettlen M, Kuwata H, Grinstein S, Schmid SL, Danuser G (2008) Robust single-particle tracking in live-cell time-lapse sequences. *Nat Methods* 5: 695 – 702

Jiang L-Y, Jiang W, Tian N, Xiong Y-N, Liu J, Wei J, Wu K-Y, Luo J, Shi X-J, Song B-L (2018) Ring finger protein 145 (RNF145) is a ubiquitin ligase for sterol-induced degradation of HMG-CoA reductase. *J Biol Chem* 293: 4047 – 4055

Karaskov E, Scott C, Zhang L, Teodoro T, Ravazzola M, Volchuk A (2006) Chronic palmitate but not oleate exposure induces endoplasmic reticulum stress, which may contribute to INS-1 pancreatic β-cell apoptosis. *Endocrinology* 147: 3398 – 3407

Kim H, Zhang H, Meng D, Russell G, Lee JN, Ye J (2013) UAS domain of Ubxd8 and FAF1 polymerizes upon interaction with long-chain unsaturated fatty acids. *J Lipid Res* 54: 2144 – 2152

Kita Y, Shindou H, Shimizu T (2019) Cytosolic phospholipase a 2 and lysophospholipid acyltransferases. *Biochim Biophys Acta Mol Cell Biol Lipids* 1864: 838 – 845

Krahmer N, Najafi B, Schueder F, Quagliarini F, Steger M, Seitz S, Kasper R, Salinas F, Cox J, Uhlenhaut NH *et al* (2018) Organellar proteomics and Phospho-proteomics reveal subcellular reorganization in diet-induced hepatic steatosis. *Dev Cell* 47: 205 – 221.e7

Lee JN, Zhang X, Feramisco JD, Gong Y, Ye J (2008) Unsaturated fatty acids inhibit proteasomal degradation of Insig-1 at a Postubiquitination step. *J Biol Chem* 283: 33772 – 33783

Lee JN, Kim H, Yao H, Chen Y, Weng K, Ye J (2010) Identification of Ubxd8 protein as a sensor for unsaturated fatty acids and regulator of triglyceride synthesis. *Proc Natl Acad Sci U S A* 107: 21424 – 21429

Levental KR, Malmberg E, Symons JL, Fan Y, Chapkin RS, Ernst R, Levental I (2020) Lipidomic and biophysical homeostasis of mammalian membranes counteracts dietary lipid perturbations to maintain cellular fitness. *Nat Commun* 11: 1339

 © 2022 The Authors

Listenberger LL, Han X, Lewis SE, Cases S, Farese RV, Ory DS, Schaffer JE (2003) Triglyceride accumulation protects against fatty acid-induced lipotoxicity. *Proc Natl Acad Sci U S A* 100: 3077 – 3082

Liu Y, Michael MD, Kash S, Bensch WR, Monia BP, Murray SF, Otto KA, Syed SK, Bhanot S, Sloop KW *et al* (2007) Deficiency of adiponectin receptor 2 reduces diet-induced insulin resistance but promotes type 2 diabetes. *Endocrinology* 148: 683 – 692

Luo Y, Rana P, Will Y (2012) Palmitate increases the susceptibility of cells to drug-induced toxicity: an *in vitro* method to identify drugs with potential contraindications in patients with metabolic disease. *Toxicol Sci* 129: 346 – 362

Ly LD, Xu S, Choi S-K, Ha C-M, Thoudam T, Cha S-K, Wiederkehr A, Wollheim CB, Lee I-K, Park K-S (2017) Oxidative stress and calcium dysregulation by palmitate in type 2 diabetes. *Exp Mol Med* 49: e291

Lytrivi M, Castell AL, Poitout V, Cnop M (2020) Recent insights into mechanisms of β-cell Lipo- and glucolipotoxicity in type 2 diabetes. *J Mol Biol* 432: 1514 – 1534

Masuda Y, Itabe H, Odaki M, Hama K, Fujimoto Y, Mori M, Sasabe N, Aoki J, Arai H, Takano T (2006) ADRP/adipophilin is degraded through the proteasome-dependent pathway during regression of lipid-storing cells. *J Lipid Res* 47: 87 – 98

Menzies SA, Volkmar N, van den Boomen DJ, Timms RT, Dickson AS, Nathan JA, Lehner PJ (2018) The sterol-responsive RNF145 E3 ubiquitin ligase mediates the degradation of HMG-CoA reductase together with gp78 and Hrd1. *eLife* 7: 391789

Miras F, Hernandez J, De La Higuera Torres PJ, Nuñez J, Martin A, De La Higuera RJ (1983) Studies on the fate of labelled palmitic acid in rat lung. *Comp Biochem Physiol C Comp Pharmacol Toxicol* 75: 179 – 183

Mota M, Banini BA, Cazanave SC, Sanyal AJ (2016) Molecular mechanisms of lipotoxicity and glucotoxicity in nonalcoholic fatty liver disease. *Metabolism* 65: 1049 – 1061

Nixon-Abell J, Obara C, Weigel A, Li D, Legant W, Xu C, Pasolli H, Harvey K, Hess H, Betzig E *et al* (2016) Increased spatiotemporal resolution reveals highly dynamic dense tubular matrices in the peripheral ER. *Science* 354: aaf3928

Palareti G, Legnani C, Cosmi B, Antonucci E, Erba N, Poli D, Testa S, Tosetto A (2016) Comparison between different D-dimer cutoff values to assess the individual risk of recurrent venous thromboembolism: analysis of results obtained in the DULCIS study. *Int J Lab Hematol* 38: 42 – 49

Palomer X, Pizarro-Delgado J, Barroso E, Vázquez-Carrera M (2018) Palmitic and oleic acid: the Yin and Yang of fatty acids in type 2 diabetes mellitus. *Trends Endocrinol Metab* 29: 178 – 190

Pei J, Millay DP, Olson EN, Grishin NV (2011) CREST - a large and diverse superfamily of putative transmembrane hydrolases. *Biol Direct* 6: 37

Perez-Riverol Y, Bai J, Bandla C, García-Seisdedos D, Hewapathirana S, Kamatchinathan S, Kundu DJ, Prakash A, Frericks-Zipper A, Eisenacher M *et al* (2022) The PRIDE database resources in 2022: a hub for mass spectrometry-based proteomics evidences. *Nucleic Acids Res* 50: D543 – D552

Piccolis M, Bond LM, Kampmann M, Pulimeno P, Chitraju C, Jayson CBK, Vaites LP, Boland S, Lai ZW, Gabriel KR *et al* (2019) Probing the global cellular responses to lipotoxicity caused by saturated fatty acids. *Mol Cell* 74: 32 – 44.e8

Pineau L, Colas J, Dupont S, Beney L, Fleurat-Lessard P, Berjeaud J-M, Bergès T, Ferreira T (2009) Lipid-induced ER stress: synergistic effects of sterols and saturated fatty acids. *Traffic* 10: 673 – 690

Radhakrishnan A, Sun L-P, Kwon HJ, Brown MS, Goldstein JL (2004) Direct binding of cholesterol to the purified membrane region of SCAP. *Mol Cell* 15: 259 – 268

Ran FA, Hsu PD, Wright J, Agarwala V, Scott DA, Zhang F (2013) Genome engineering using the CRISPR-Cas9 system. *Nat Protoc* 8: 2281 – 2308

Rape M, Hoppe T, Gorr I, Kalocay M, Richly H, Jentsch S (2001) Mobilization of processed, membrane-tethered SPT23 transcription factor by CDC48 (UFD1/NPL4), a ubiquitin-selective chaperone. *Cell* 107: 667 – 677

van Rijn JM, Ardy RC, Kuloğlu Z, Härter B, van Haaften-Visser DY, van der Doef HPJ, van Hoesel M, Kansu A, van Vugt AHM, Thian M *et al* (2018) Intestinal failure and aberrant lipid metabolism in patients with DGAT1 deficiency. *Gastroenterology* 155: 130 – 143.e15

Ruiz M, Bodhicharla R, Svensk E, Devkota R, Busayavalasa K, Palmgren H, Ståhlman M, Boren J, Pilon M (2018) Membrane fluidity is regulated by the C. elegans transmembrane protein FLD-1 and its human homologs TLCD1/2. *eLife* 7: e40686

Ruiz M, Bodhicharla R, Ståhlman M, Svensk E, Busayavalasa K, Palmgren H, Ruhanen H, Boren J, Pilon M (2019a) Evolutionarily conserved long-chain acyl-CoA synthetases regulate membrane composition and fluidity. *eLife* 8: e47733

Ruiz M, Ståhlman M, Borén J, Pilon M (2019b) AdipoR1 and AdipoR2 maintain membrane fluidity in most human cell types and independently of adiponectin. *J Lipid Res* 60: 995 – 1004

Ruiz M, Palmgren H, Henricsson M, Devkota R, Jaiswal H, Maresca M, Bohlooly-Y M, Peng X-R, Boren J, Pilon M (2021) Extensive transcription mis-regulation and membrane defects in AdipoR2-deficient cells challenged with saturated fatty acids. *Biochim Biophys Acta Mol Cell Biol Lipids* 1866: 158884

Schuberth C, Buchberger A (2008) UBX domain proteins: major regulators of the AAA ATPase Cdc48/p97. *Cell Mol Life Sci* 65: 2360 – 2371

Sezgin E, Levental I, Mayor S, Eggeling C (2017) The mystery of membrane organization: composition, regulation and roles of lipid rafts. *Nat Rev Mol Cell Biol* 18: 361 – 374

Sinensky M (1974) Homeoviscous adaptation - a homeostatic process that regulates the viscosity of membrane lipids in *Escherichia coli*. *Proc Natl Acad Sci U S A* 71: 522 – 525

Stevenson J, Luu W, Kristiana I, Brown AJ (2014) Squalene mono-oxygenase, a key enzyme in cholesterol synthesis, is stabilized by unsaturated fatty acids. *Biochem J* 461: 435 – 442

Stevenson J, Huang EY, Olzmann JA (2016) Endoplasmic reticulum-associated degradation and lipid homeostasis. *Annu Rev Nutr* 36: 511 – 542

Surma MA, Klose C, Peng D, Shales M, Mrejen C, Stefanko A, Braberg H, Gordon DE, Vorkel D, Ejsing CS *et al* (2013) A lipid E-MAP identifies Ubx2 as a critical regulator of lipid saturation and lipid bilayer stress. *Mol Cell* 51: 519 – 530

Svensk E, Ståhlman M, Andersson CH, Johansson M, Borén J, Pilon M (2013) PAQR-2 regulates fatty acid desaturation during cold adaptation in C. elegans. *PLoS Genet* 9: e1003801

Svensk E, Devkota R, Ståhlman M, Ranji P, Rauthan M, Magnusson F, Hammarsten S, Johansson M, Borén J, Pilon M (2016) Caenorhabditis elegans PAQR-2 and IGLR-2 protect against glucose toxicity by modulating membrane lipid composition. *PLoS Genet* 12: 3 – 4

Takahashi Y, Shinoda A, Kamada H, Shimizu M, Inoue J, Sato R (2016) Perilipin2 plays a positive role in adipocytes during lipolysis by escaping proteasomal degradation. *Sci Rep* 6: 20975

Tanabe H, Fujii Y, Okada-Iwabu M, Iwabu M, Nakamura Y, Hosaka T, Motoyama K, Ikeda M, Wakiyama M, Terada T *et al* (2015) Crystal structures of the human adiponectin receptors. *Nature* 520: 312 – 316

Tinevez JY, Perry N, Schindelin J, Hoopes GM, Reynolds GD, Laplantine E, Bednarek SY, Shorte SL, Eliceiri KW (2017) TrackMate: an open and extensible platform for single-particle tracking. *Methods* 115: 80 – 90

© 2022 The Authors

Van Meer G, Voelker DR, Feigenson GW (2008) Membrane lipids: where they are and how they behave. *Nat Rev Mol Cell Biol* 9: 112 – 124

Vance JE (2015) Phospholipid synthesis and transport in mammalian cells. *Traffic* 16: 1 – 18

Vasiliauskaité-Brooks I, Sounier R, Rochaix P, Bellot G, Fortier M, Hoh F, De Colibus L, Bechara C, Saied EM, Arenz C *et al* (2017) Structural insights into adiponectin receptors suggest ceramidase activity. *Nature* 544: 120 – 123

Volmer R, Ron D (2015) Lipid-dependent regulation of the unfolded protein response. *Curr Opin Cell Biol* 33: 67 – 73

Weinberg JM (2006) Lipotoxicity. *Kidney Int* 70: 1560 – 1566

Woldseth B, Christensen E, Christophersen B (1993) Incorporation of stearic acid (18:0) and palmitic acid (16:0) in phospholipid molecular species studied in isolated rat liver cells. *Biochim Biophys Acta* 1167: 296 – 302

Xu G, Sztalryd C, Lu X, Tansey JT, Gan J, Dorward H, Kimmel AR, Londos C (2005) Post-translational regulation of adipose differentiation-related protein by the ubiquitin/proteasome pathway. *J Biol Chem* 280: 42841 – 42847

Yamauchi T, Nio Y, Maki T, Kobayashi M, Takazawa T, Iwabu M, Okada-Iwabu M, Kawamoto S, Kubota N, Kubota T *et al* (2007) Targeted disruption of AdipoR1 and AdipoR2 causes abrogation of adiponectin binding and metabolic actions. *Nat Med* 13: 332 – 339

Yang Y, Lee M, Fairn GD (2018) Phospholipid subcellular localization and dynamics. *J Biol Chem* 293: 6230 – 6240

Zhu XG, Nicholson Puthenveedu S, Shen Y, La K, Ozlu C, Wang T, Klompstra D, Gultekin Y, Chi J, Fidelin J *et al* (2019) CHP1 regulates compartmentalized Glycerolipid synthesis by activating GPAT4. *Mol Cell* 74: 45 – 58.e7

License: This is an open access article under the terms of the Creative Commons Attribution License, which permits use, distribution and reproduction in any medium, provided the original work is properly cited.

 © 2022 The Authors

Expanded View Figures

Figure EV1. RNF145 levels are regulated by FAs. Subcellular localisation of ADIPOR2.

A, B Whole-cell TMT-based quantitative proteomic analysis of HEK-293T cells treated as described in Fig 1A. Protein levels are expressed as relative abundance in PA vs BSA (A) or OA vs BSA-treated cells (B). Proteins with fold abundance changes ≥ |1.5| ($q \leq 0.05$) are labelled. ADIPOR2 and RNF145 are indicated in bold red. Proteins with roles in lipid metabolism and/or known to be fatty acid-regulated are shown in green. $N = 3$ biological replicates.

C RNF145 shows altered expression upon treatment with PA or OA. HEK-293T cells treated with PA/OA (400 µM) or BSA (vehicle control) for 6 h were examined by immunoblotting. An asterisk (*) indicates non-specific bands. $N = 3$ biological replicates.

D Validation of result in Fig 1B using independent HeLa single-cell clones knocked out for RNF145 (RNF145 KO #1–3). Since RNF145 and gp78 also coordinate the degradation of HMG-CoA reductase (HMGCR), we addressed whether gp78 is involved in ADIPOR2 degradation by knocking out gp78 (gp78 KO #1, #2) or RNF145 in combination with gp78 (RNF + gp78 KO #1–3). The stabilising effect of RNF145 on ADIPOR2 was not notably enhanced by concomitant gp78 knockout (lanes 7–9). An asterisk (*) indicates non-specific bands.

E Huh7 (i) and U2-OS cells (ii) were transfected with sgRNAs targeting either RNF145 (gRNF145) or B2M (gCTR) followed by immunoblot analysis of endogenous ADIPOR2. The asterisk (*) denotes non-specific bands.

F HEK-293T cells were transfected with 4 different sgRNAs targeting RNF145 (gRNF145 #1–4) or a sgRNA targeting B2M (gCTR). The asterisk (*) denotes non-specific bands. LE, long exposure.

G Regulation of RNF145 and ADIPOR2 in a primary cell line model. Primary human microvascular endothelial cells (MVECs) were supplemented with PA (400 µM), OA (400 µM) or BSA (vehicle control) for 6 h and RNF145 and ADIPOR2 expression analysed by immunoblotting.

H HEK-293T cells stably expressing a farnesylated-Em (mEmerald-Farnesyl-5) membrane marker and ADIPOR2-S were visualised by immunofluorescence microscopy. Micrographs ((a)–(c)) represent magnifications of the indicated areas in the merge panel. Blue arrows highlight areas of co-localisation between farnesyl-Em and ADIPOR2-S, possibly representing ER-plasma membrane contact sites. White arrows indicate regions where the ER and plasma membrane signals do not overlap. Scale bars = 10 µm or 5 µm ((a)–(c)).

© 2022 The Authors

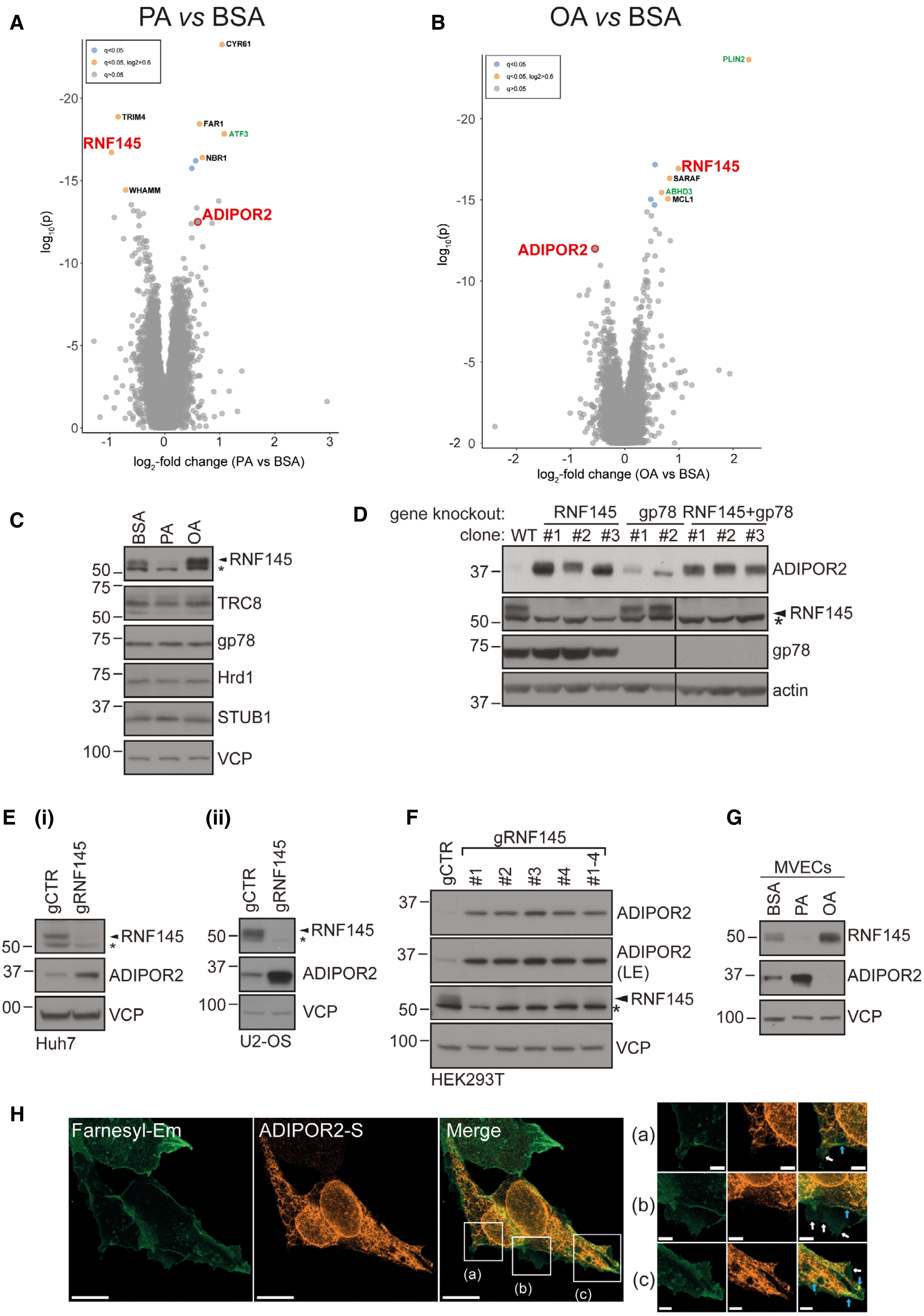

Figure EV1.

 © 2022 The Authors

Figure EV2. RNF145 depletion stabilises ADIPOR2-mRuby2 and ADIPOR2 is an ERAD substrate.

A ADIPOR2-depleted HEK-293T cells reconstituted with C-terminally mRuby2-tagged ADIPOR2 (ADIPOR2-HA-mRuby2) were depleted of RNF145 (gRNF145; red) or B2M (gCTR; blue) and fluorescence intensity was measured by flow cytometry.

B Immunoblot (IB) analysis of ADIPOR2-HA-mRuby2 expressed in ADIPOR2-depleted (gADIPOR2) cells. ADIPOR2-HA-mRuby2 was detected using a specific anti-HA antibody.

C ADIPOR2 is rapidly degraded at steady-state and can be rescued by proteasome inhibition. Representative ^{35}S pulse-chase in HeLa cells stably expressing ADIPOR2-HA ± MG132 (10 μg/ml, 2 h). *non-specific bands. Quantification of ^{35}S-labelled ADIPOR2-HA normalised to $t = 0$ h is shown below each lane.

D ADIPOR2-HA was stably expressed in HEK-293T cells, HA-affinity purified and interaction partners identified by tandem mass spectrometry. Proteins shown were not present in the negative control (empty vector expressing HEK-293Ts) and are therefore considered specific interaction partners of ADIPOR2-HA.

E Flow cytometry analysis of ADIPOR2-depleted HEK-293T cells complemented with ADIPOR2 tagged C-terminally with Clover (ADIPOR2-HA-Clover) and transfected with indicated sgRNAs. Knockdown efficiencies are shown in (ii). ADIPOR2-HA-Clover expression is shown in (iii). *non-specific bands.

F HEK-293T cells expressing ADIPOR2-HA-Clover were treated with VCP inhibitor (NMS-873, 10 μM, 6 h) or proteasome inhibitor (MG132, 10 μg/ml, 6 h) and ADIPOR2-HA-Clover expression was analysed by flow cytometry.

© 2022 The Authors

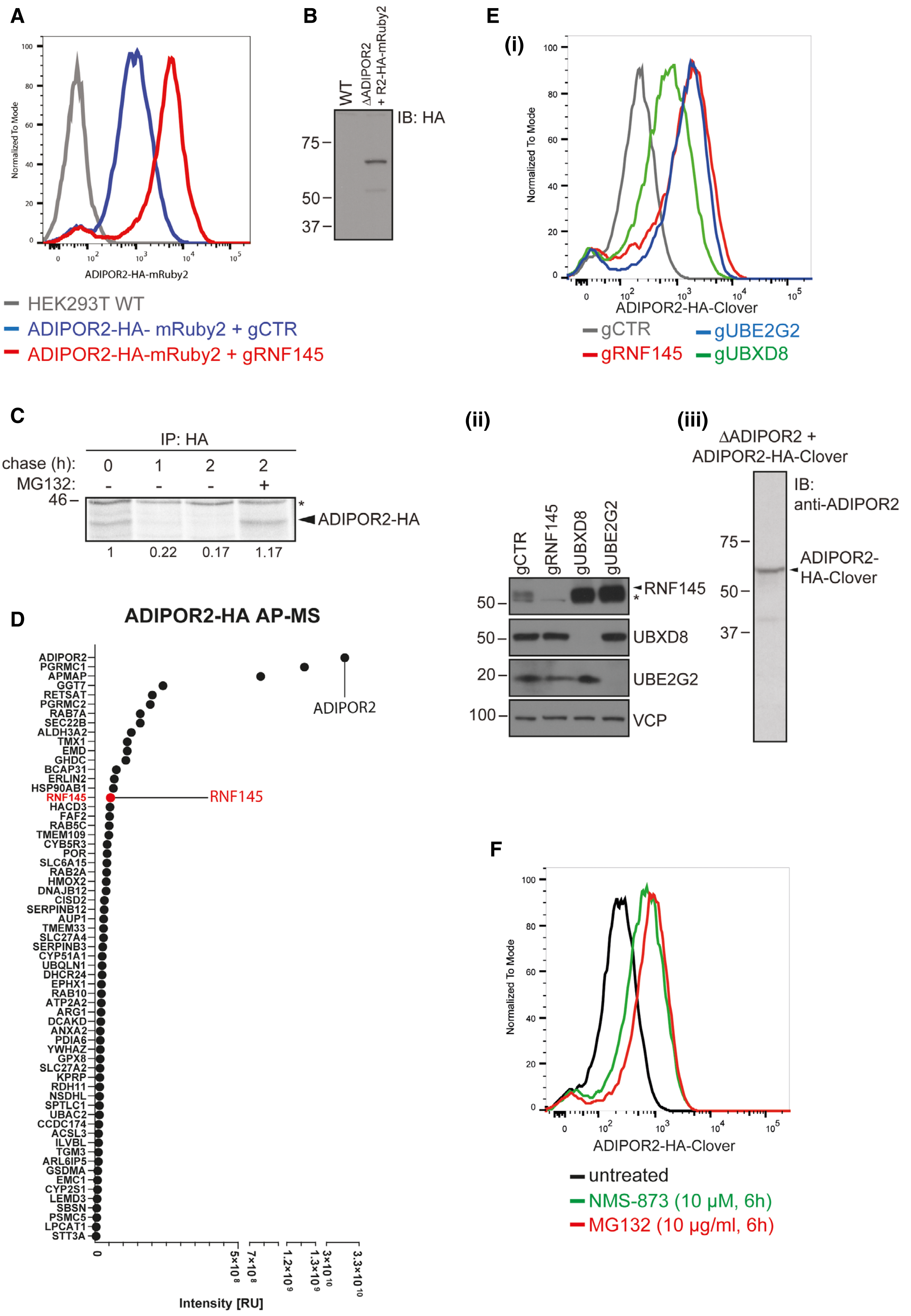

Figure EV2.

 © 2022 The Authors

Figure EV3. Regulation of RNF145 transcript and protein levels in the presence of excess OA/PA.

A HEK-293T cells were treated with PA, OA (each 400 μM, 6 h), or BSA (vehicle control). Quantitative PCR analysis for RNF145 was performed on whole-cell mRNA extracts, normalised to actin transcript levels and compared to BSA treatment (dotted line). Boxplots represent the median, first and third quantiles. Upper and lower whiskers extend to values up to 1.5-fold interquartile range. *P* values were calculated with a Wilcoxon rank-sum test. Benjamini-Hochberg-adjusted *P* values $\leq$ 0.05 are indicated (*).

B Assessment of the involvement of OA sensing by UBXD8 in the RNF145-mediated degradation of ADIPOR2. HEK-293T cells depleted of UBXD8 (gUBXD8) were stably complemented with an empty vector, UBXD8 (UBXD8 WT) or the OA-insensitive $UBXD8^{K167E,R168E,R171E,K239E,R241E,R242E}$ mutant (mUBXD8), treated with PA or OA (200 μM, 6 h) and indicated proteins visualised by immunoblotting. *non-specific bands.

C Two clonal ADIPOR2 knockout HEK-293T cell lines (clone #1 or 2) or wild-type (WT) cells were treated with PA, OA or BSA vehicle control (veh.) and analysed by immunoblotting. LE, long exposure. *non-specific bands.

D Loss of UBE2G2 renders RNF145 levels fatty acid-independent. HEK293 cells were depleted of UBE2G2 (gUBE2G2) or B2M (gCTR) as described in Fig 2D and treated with BSA, PA or OA (200 μM) for 6 h. *non-specific bands.

E HeLa cells expressing HMGCR-Clover or HeLa HMGCR-Clover cells depleted of INSIG1/2 form Menzies *et al* (2018) were treated with BSA (control) or 400 μM PA or OA for 6 h, lysed and prepared for western blot with ADIPOR2 antibodies (i). (ii) Knockout of INSIG1 and 2 was confirmed using flow cytometry to measure HMGCR-Clover stabilisation. Cells were sterol depleted (DMEM +10% LPDS + 10 mM mevastatin + 50 mM mevalonate) for 20 h and treated with sterols (25-hydroxycholesterol (2 mg/ml), cholesterol (20 mg/ml)) for 2 h to trigger INSIG1/2-dependent HMGCR degradation.

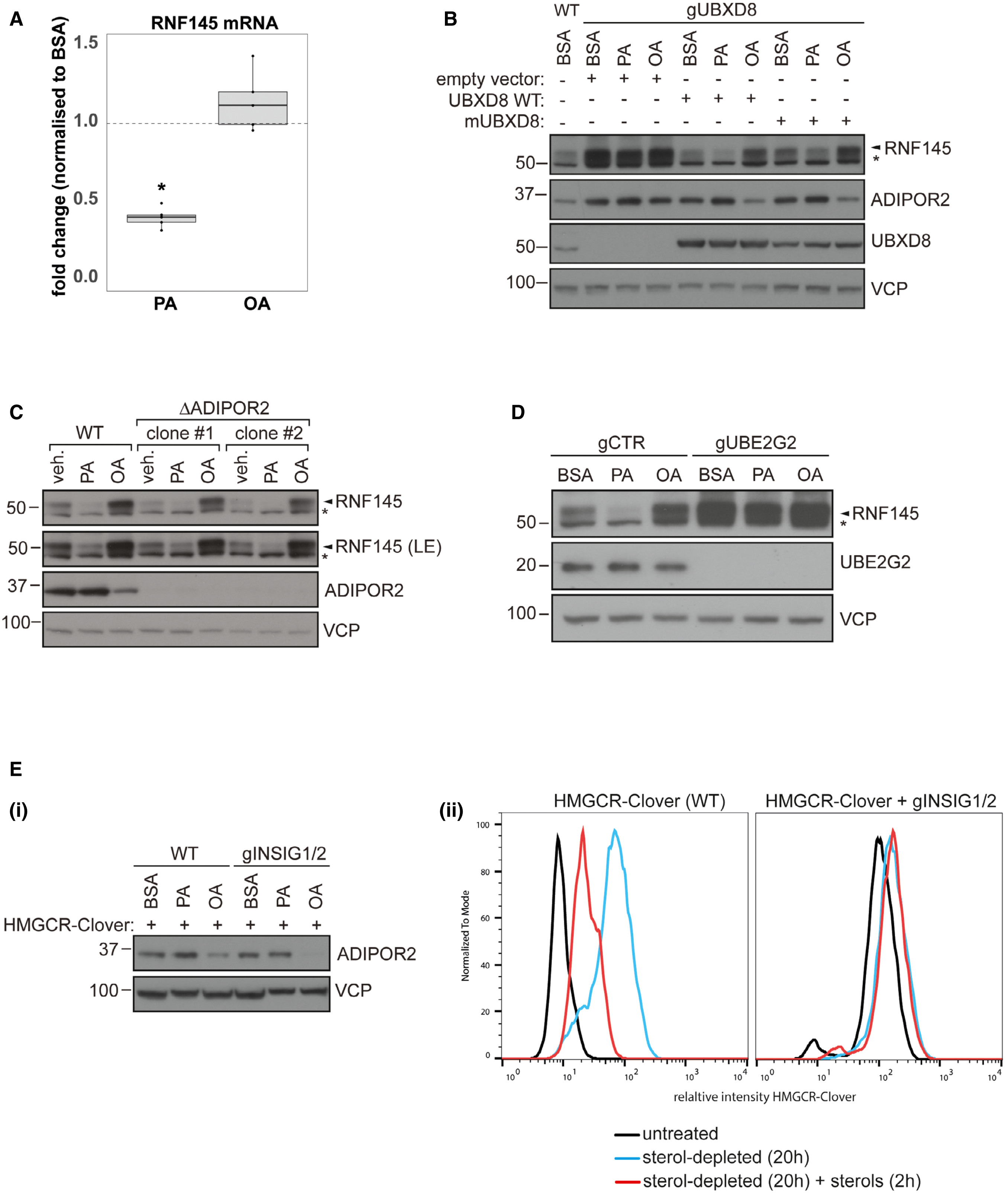

Figure EV3.

 © 2022 The Authors

Figure EV4. Quantitative analysis of lipids, lipid precursors and fatty acids in HEK-293T cells.

A Cells stably depleted of RNF145 (gRNF145), ADIPOR2 (gADIPOR2), or B2M (gCTR) were treated with BSA for 20h. All values were normalised to gCTR (dotted line). Boxplots represent the median, first and third quantiles. Upper and lower whiskers extend to values up to 1.5-fold interquartile range. $N = 6$ biological replicates. Changes were compared to gCTR and significance testing was performed using the Wilcoxon Rank Sum Test. $^{**}q \leq 0.01$.

B Lipidomic analysis of lipids containing deuterated PA (D3-PA) or its derivatives. Heat maps represent abundance changes relative to gCTR at the indicated time points. In PC (32:0), both side chains are deuterated. Statistical significance was calculated using one-way ANOVA. Benjamini-Hochberg-adjusted P-values ≤ 0.05 are indicated (*). PC, phosphatidylcholine; LPC, lysophosphatidylcholine; DAG, diacylglycerol; TAG, triacylglycerol.

© 2022 The Authors

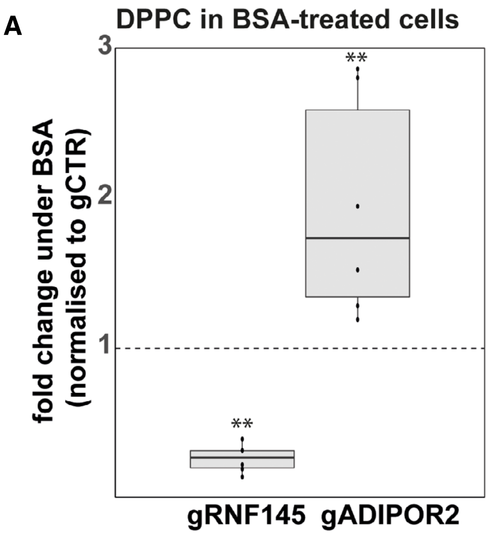

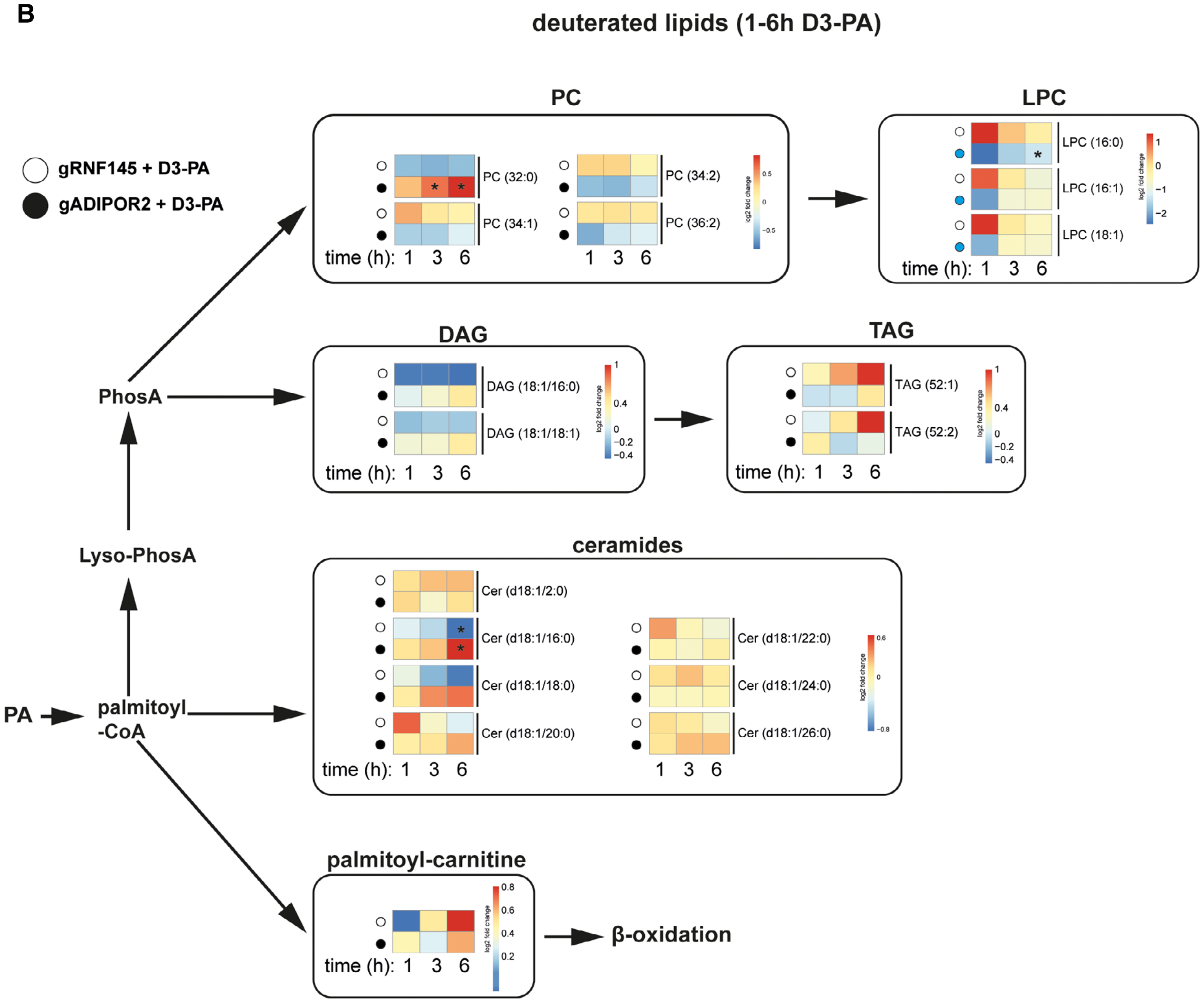

Figure EV4.

© 2022 The Authors

Appendix Figures

Appendix Materials and Methods

Volkmar N. et al.,

Contents	Page
**Appendix Figure S1.** Confirmation of knockdown efficiencies and expression of ADIPOR2 constructs.	**2**
**Appendix Figure S2.** RNF145/ADIPOR2 knockdown efficiencies in cells used for FRAP and SMT analysis.	**3**
**Appendix Materials and Methods:**<br><br>**Collection and processing of Mass spectrometry data.** Workflow (A) and data processing (B) of data represented in **Figure 1A & B** and **EV Fig 1A**.	**4-7**

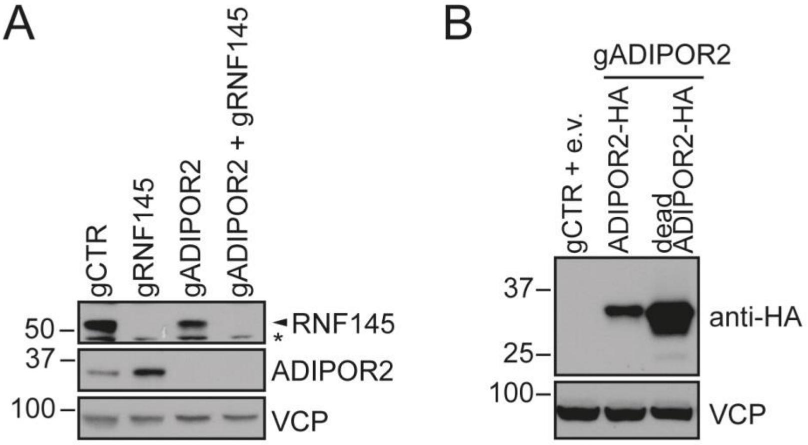

Appendix Figure S1. Confirmation of knockdown efficiencies and expression of ADIPOR2 constructs. (A) Cells (gCTR or gRNF145) in used for experiments in **Figure 5A** were analysed by immunoblotting before treatment with FAs. LE, long exposure; *non-specific bands. **(B)** Immunoblot analysis of cells used in **Figure 5C**. ADIPOR2-depleted cells were stably complemented with epitope-tagged WT ADIPOR2 (ADIPOR2-HA) or a catalytically inactive ADIPOR2 mutant (dead ADIPOR2-HA). Expression levels of recombinant ADIPOR2 variants before exposure to FAs were determined by immunoblot analysis using a specific anti-HA antibody.

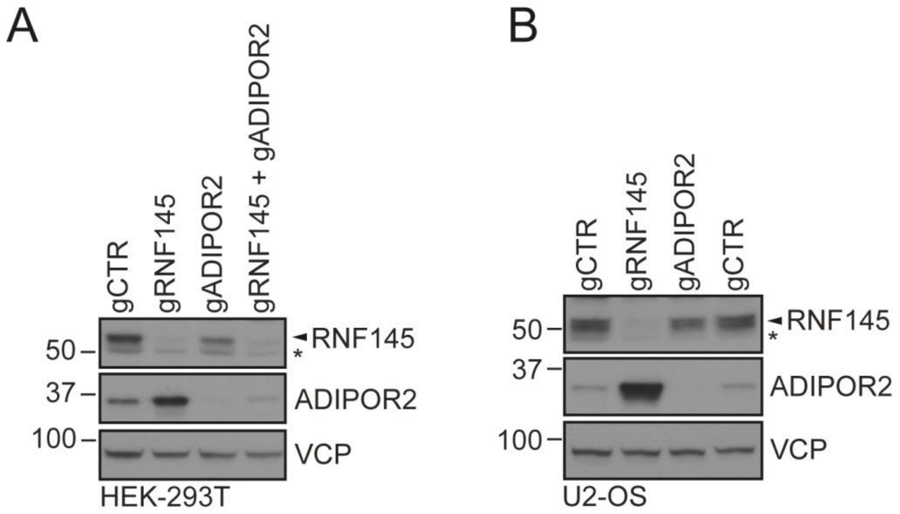

Appendix Figure S2. RNF145/ADIPOR2 knockdown efficiencies in cells used for FRAP and SMT analysis. (A) CRISPR/Cas9-mediated stable depletion of RNF145 (gRNF145), ADIPOR2 (gADIPOR2), or B2M (gCTR) in HEK-293T cells used in FRAP analysis (**Figure 6A**). **(B)** CRISPR/Cas9-mediated stable depletion of RNF145 (gRNF145), ADIPOR2 (gADIPOR2), or B2M (gCTR) in U2-OS cells used for SMT analysis (**Figure 6B**). Cell lines were generated by transfecting U2-OS cells with pools of 4 sgRNAs against the indicated targets. Knockdown cell pools were selected by puromycin for 72h.

(A) MS Method - TMT

Start Time (min): **0**
End Time (min): **190**
Cycle Time (sec): **3**

Master Scan:
- MS OT
  - Detector Type: **Orbitrap**
  - Orbitrap Resolution: **120000**
  - Mass Range: **Normal**
  - Use Quadrupole Isolation: **True**
  - Scan Range (m/z): **400-1500**
  - RF Lens (%): **60**
  - AGC Target: **Custom**
  - Normalized AGC Target (%): **125**
  - Maximum Injection Time Mode: **Custom**
  - Maximum Injection Time (ms): **50**
  - Microscans: **1**
  - Data Type: **Profile**
  - Polarity: **Positive**
  - Source Fragmentation: **Disabled**
  - Scan Description:

Filters:
- MIPS
  - Monoisotopic Peak Determination: **Peptide**
- Charge State
  - Include charge state(s): **2-7**
  - Include undetermined charge states: **False**
- Dynamic Exclusion
  - Use Common Settings: **False**
  - Exclude after n times: **1**
  - Exclusion duration (s): **90**
  - Mass Tolerance: **ppm**
  - Low: **10**
  - High: **10**
  - Exclude Isotopes: **True**
  - Perform dependent scan on single charge state per precursor only: **True**
- Intensity
  - Filter Type: **Intensity Threshold**
  - Intensity Threshold: **5.0e3**

- Data Dependent
  - Data Dependent Mode: **Cycle Time**
  - Time between Master Scans (sec): **3**

- Scan Event Type 1:
  - Scan:
  - ddMS^2 IT CID
    - Isolation Mode: **Quadrupole**
    - Isolation Window (m/z): **0.7**
    - Isolation Offset: **Off**
    - Activation Type: **CID**
    - Collision Energy Mode: **Fixed**
    - CID Collision Energy (%): **35**
    - CID Activation Time (ms): **10**
    - Activation Q: **0.25**
    - Multistage Activation: **False**
    - Detector Type: **Ion Trap**
    - Ion Trap Scan Rate: **Rapid**
    - Mass Range: **Normal**
    - Scan Range Mode: **Auto**
    - AGC Target: **Custom**
    - Normalized AGC Target (%): **80**
    - Maximum Injection Time Mode: **Auto**
    - Microscans: **1**
    - Data Type: **Centroid**
    - Scan Description:

  - Filters:
    - Precursor Selection Range
      - Selection Range Mode: **Mass Range**
      - Mass Range (m/z): **400-2000**
    - Precursor Ion Exclusion
      - Exclusion mass width: **m/z**
      - Low: **19**
      - High: **7**
    - Isobaric Tag Loss Exclusion
      - Reagent: **TMT**

  - Data Dependent
    - Data Dependent Mode: **Scans Per Outcome**

  - Scan Event Type 1:
    - Scan:
    - ddMS^3 OT HCD
      - MS^n Level: **3**
      - Synchronous Precursor Selection: **True**
      - Number of SPS Precursors: **10**
      - MS Isolation Window (m/z): **2**
      - MS2 Isolation Window (m/z): **2**
      - Isolation Offset: **Off**
      - Activation Type: **HCD**
      - HCD Collision Energy (%): **65**
      - Detector Type: **Orbitrap**
      - Orbitrap Resolution: **50000**
      - Mass Range: **Normal**
      - Scan Range Mode: **Define m/z range**
      - Scan Range (m/z): **100-1000**
      - AGC Target: **Custom**
      - Normalized AGC Target (%): **40**
      - Maximum Injection Time Mode: **Custom**
      - Maximum Injection Time (ms): **120**
      - Microscans: **1**
      - Data Type: **Profile**
      - Use EASY-IC™: **False**
      - Scan Description:
      - Number of Dependent Scans: **3**

MS Method – AP-MS

- Start Time (min): **0**
- End Time (min): **87**
- Cycle Time (sec): **2**

Master Scan:

- MS OT
  - Detector Type: **Orbitrap**
  - Orbitrap Resolution: **120000**
  - Mass Range: **Normal**
  - Use Quadrupole Isolation: **True**
  - Scan Range (m/z): **400-1600**
  - RF Lens (%): **60**
  - AGC Target: **Standard**
  - Maximum Injection Time Mode: **Custom**
  - Maximum Injection Time (ms): **50**
  - Microscans: **1**
  - Data Type: **Profile**
  - Polarity: **Positive**
  - Source Fragmentation: **Disabled**
  - Scan Description:

Filters:

- Intensity
  - Filter Type: **Intensity Threshold**
  - Intensity Threshold: **5.0e3**
- MIPS
  - Monoisotopic Peak Determination: **Peptide**
- Dynamic Exclusion
  - Use Common Settings: **False**
  - Exclude after n times: **1**
  - Exclusion duration (s): **20**
  - Mass Tolerance: **ppm**
  - Low: **5**
  - High: **5**
  - Exclude Isotopes: **True**
  - Perform dependent scan on single charge state per precursor only: **True**
- Charge State
  - Include charge state(s): **2-4**
  - Include undetermined charge states: **False**

- Data Dependent
  - Data Dependent Mode: **Cycle Time**
  - Time between Master Scans (sec): **2**

- Scan Event Type 1:
  - Scan:
  - ddMS^2 IT HCD
    - Isolation Mode: **Quadrupole**
    - Isolation Window (m/z): **1.6**
    - Isolation Offset: **Off**
    - Activation Type: **HCD**
    - Collision Energy Mode: **Fixed**
    - HCD Collision Energy (%): **34**
    - Detector Type: **Ion Trap**
    - Ion Trap Scan Rate: **Rapid**
    - Mass Range: **Normal**
    - Scan Range Mode: **Auto**
    - AGC Target: **Standard**
    - Maximum Injection Time Mode: **Auto**
    - Microscans: **1**
    - Data Type: **Centroid**
    - Scan Description:

(B) Searching Schematic

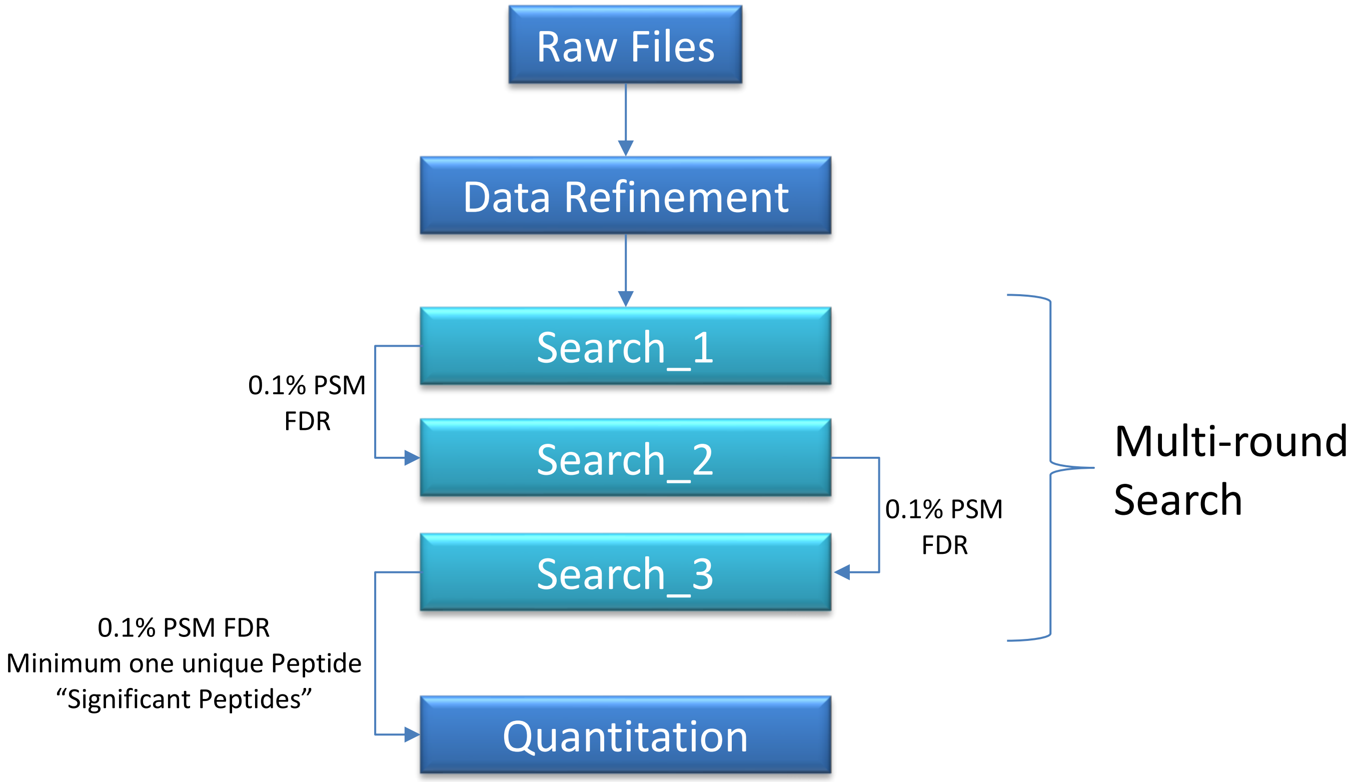

Data Refinement

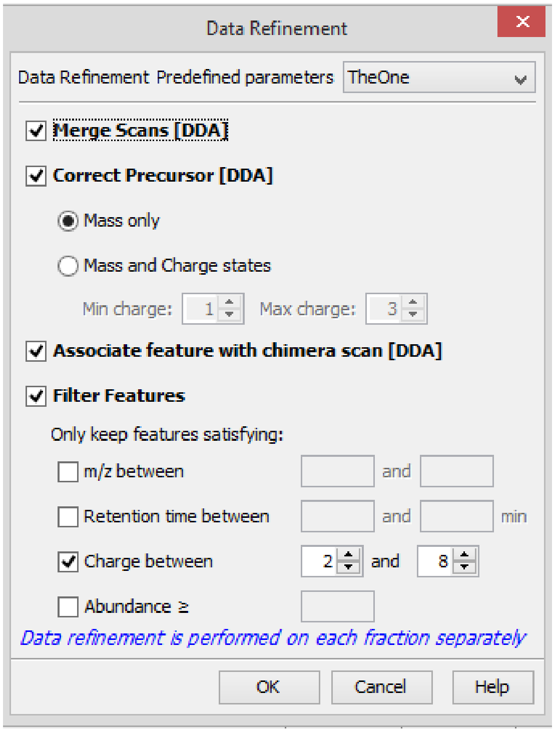

Search_1

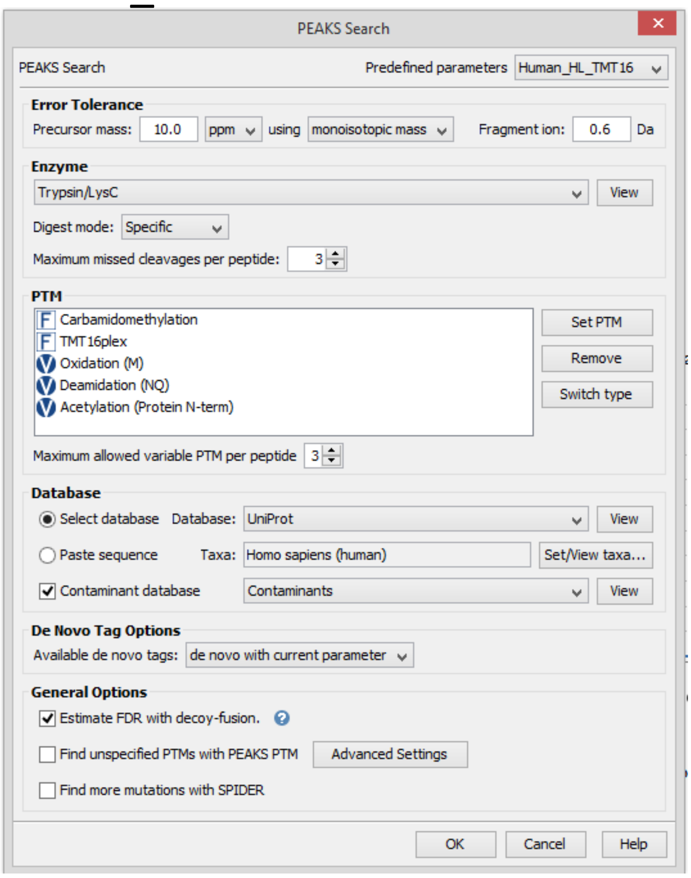

Cleavage Rules

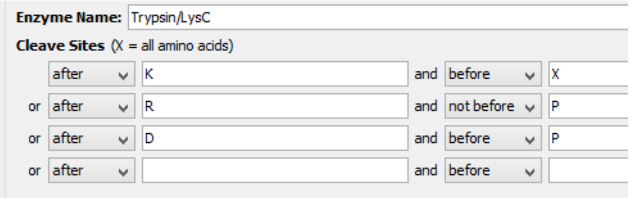

Search_2

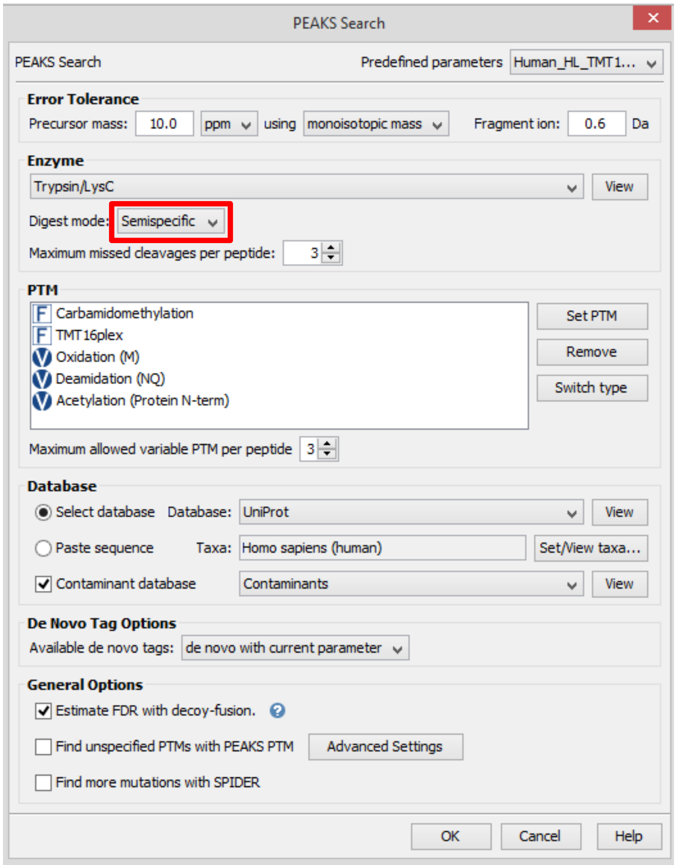

Search_3

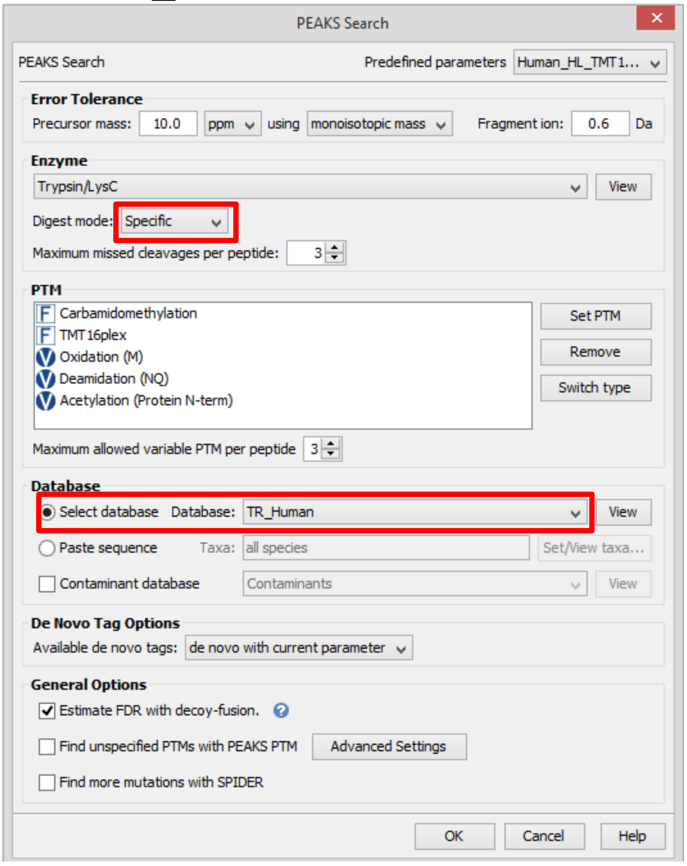

Quantitation

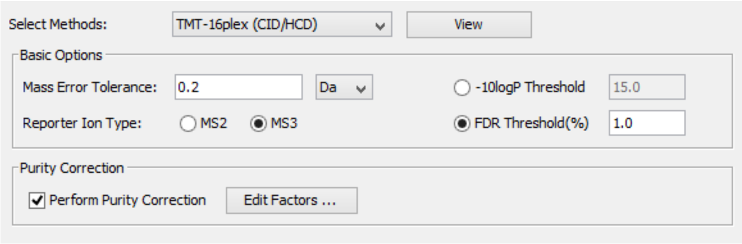

Appendix Materials and Methods; Collection and processing of Mass spectrometry data. Workflow (A) and data processing (B) of data represented in **Figure 1A & B** and **EV Fig 1A**.
