# Supplementary material for: Regulation of membrane fluidity by RNF145‐triggered degradation of the lipid hydrolase ADIPOR2
Source: EMBO J. 2022 Aug 22;41(19):e110777. doi: 10.15252/embj.2022110777 (PMC9531299; doi:10.15252/embj.2022110777)
Supplement: Supplementary file 6 [file EMBJ-41-e110777-s004.pdf]

THE EMBO JOURNAL

Regulation of membrane fluidity by RNF145-triggered degradation of the lipid hydrolase ADIPOR2

Norbert Volkmar, Christian Gawden-Bone, James Williamson, Jonathan Nixon-Abell, James West, Peter St George-Hyslop, Arthur Kaser, and Paul Lehner
DOI: 10.15252/embj.2022110777

Corresponding author(s): Paul Lehner (pjl30@cam.ac.uk)

Review Timeline:

Submission Date:	26th Jan 22
Editorial Decision:	25th Feb 22
Revision Received:	28th Jun 22
Editorial Decision:	12th Jul 22
Revision Received:	18th Jul 22
Accepted:	20th Jul 22

Editor: Hartmut Vodermaier

Transaction Report:

(Note: With the exception of the correction of typographical or spelling errors that could be a source of ambiguity, letters and reports are not edited. Depending on transfer agreements, referee reports obtained elsewhere may or may not be included in this compilation. Referee reports are anonymous unless the Referee chooses to sign their reports.)

Thank you for submitting your study on RNF145 and membrane phospholipid sensing/regulation for our consideration. Given the generally positive comments by three expert referees, whose reports are copied below, we shall be happy to pursue an adequately revised version of the manuscript further for EMBO Journal publication.

As you will see, referee 3 has a number of presentational issues that I consider all well-taken - in particular, please capitalize on the extended format of an EMBO Journal article (with 7-9 main figures, and up to 5 Expanded View figures that would also be typeset and directly available in the HTML version), in order to make the article more reader-friendly.

In addition, referee 2 raises several experimental queries, and while I realize that some of them might be further-reaching/beyond the scope of the present analysis, I would nevertheless ask you to carefully consider and respond to all of these points. In particular, it would be important to improve the (co-)localization data pointed out by the reviewer, and to follow up on some of their suggestions for deepening the understanding of saturation sensing by RNF145. Once you had a chance to consider the reports in detail, I would be happy to briefly discuss with you how the various points might be addressable within the scope of a revision.

Detailed information on preparing, formatting and uploading a revised manuscript can be found below and in our Guide to Authors. Thank you again for the opportunity to consider this work for The EMBO Journal, and I look forward to hearing from you in due time.

Referee #1:

Membrane lipid composition has broad roles in the regulation of cell biology, influencing signal transduction, organelle biology and function, vesicular trafficking, and many other important cellular processes. Many current paradigms focus on the sensing and regulation of cholesterol. In contrast, how cells sense phospholipid saturate remains more poorly understood.

The current manuscript addresses this gap in knowledge. Initial unbiased proteomic analyses revealed a strong reduction of the ER E3 ligase RNF145 and accumulation of ADIPOR2 in response to treatment with the saturated fatty acid palmitate. In addition, ADIPOR2 accumulated in proteomics experiments examining RNF145 KO cells. This observed anti-correlation suggested a connection between the two proteins. ADIPOR2 is a lipid hydrolase known to regulate membrane saturate and rigidification. Volkmar proceed to characterize the relationship of RNF145 and ADIPOR2 in a beautiful series of rigorous and well controlled experiments. Their data strongly support a model in which RNF145-ADIPOR2 constitute a regulatory pathway to govern membrane lipid homeostasis. Under periods of membrane saturation (e.g., palmitate treatment) the interaction between RNF145 and ADIPOR2 is reduce and RNF145 is degraded through autoubiquitination. ADIPOR2 accumulates and, through its lipid hydrolase activity, regulates membrane lipid saturation. Under conditions in which unsaturated fatty acids are present (e.g., oleate treatment), ADIPOR2 is targeted by RNF145 for ERAD together with UBE2G2, UBXD8, and VCP. Lipidomics, FRAP, and single molecule analysis support the conclusions that this pathway governs membrane saturation and rigidity.

Overall, this is an exceptional study. The experiments are well designed, the conclusions are well supported, and the manuscript is well written. I appreciate how the authors judiciously consider various models in the discussion section. The findings are important and impactful, raising many significant questions for future studies. I commend the authors, have no concerns, and strongly recommend this article for publication.

-James Olzmann

Referee #2:

In this manuscript, the authors report that FAs regulate the auto-ubiquitination and stability of RNF145. In unsaturated lipid membranes, RNF145 promotes the ubiquitination and degradation of ADIPOR2. In membranes enriched in saturated FAs, RNF145 is rapidly degraded and therefore ADIPOR2 is stabilized to restore membrane lipid homeostasis by its hydrolase activity.

Here are the questions and suggestions:

What is the mechanism for RNF145 sensing the FA saturation? Is the transmembrane domain of RNF145 important for this event? TRC8 was not sensitive to FAs according to the authors' report. The difference between RNF145 and TRC8 may be helpful to address this question. Is the FA compositions in membrane sensed by RNF145, UBXD8 or other proteins? Is FA directly sensed or the FA in phospholipids sensed?

The authors should map the exact binding sequences of ADIPOR2 and RNF145.

The authors should map the exact ubiquitination site of ADIPOR2.

The ADIPOR1 and ADIPOR2 proteins are plasma membrane proteins. In figure S2C, the authors show that ADIPOR2 accumulated in the ER in RNF145 depleted cells. The image quality is poor, please show high quality images. As RNF145 is an ER protein, the authors should show the colocalization between RNF145 and ADIPOR2 with high quality image.

I noticed that the RNF145 transcript level decreased much in PA treatment (Figure S5). As RNF145 transcription is regulated by LXR, it seems that the PA treatment inhibited LXR activity. LXR also regulates fatty acid synthesis by regulating SREBP-1c and FASN expression; I think LXR may be important in the model reported here. So I suggest the authors try LXR agonist to activates RNF145 transcription, and check the ADIPOR2 protein level and the membrane fluidity.

Is insig required for the RNF145-mediated degradation of ADIPOR? Can UBXD8 regulate the level of ADIPOR2 under different FAs stimulation?

Referee #3:

Review on "Regulation of membrane fluidity by RNF145-triggered degradation of the lipid hydrolase ADIPOR2" submitted to EMBO J.

In this elegant study the authors use a proteomic approach to identify proteins affected by the supplementation of either saturated or unsaturated fatty acids to the medium. This pragmatic approach leads to the identification of the lipid hydrolase ADIPOR2 as the most upregulated and the E3 ubiquitin ligase RNF145 as the most downregulated protein under conditions of palmitate (PA) supplementation. Remarkably, the lipid hydrolase ADIPOR2 has been previously involved in protecting cells from an overly saturated membrane lipid composition, as properly referenced by the authors.

The initial observation that RNF145 and ADIPOR2 are affected by lipid metabolic cues, is elegantly followed up upon by experiments providing strong evidence that RNF145 directly regulates the level of ADIPOR2 in a lipid-dependent fashion. The authors convincingly show that the E3 ubiquitin ligase RNF145 mediates ADIPOR2 ubiquitylation as a signal for its degradation via an ER-associated degradation (ERAD) pathway. Elegant controls including knockout- and rescue experiments with lysine-free and catalytically dead variants (of either RNF145 and/or ADIPOR2) in the presence and the absence of the relevant E2 enzyme UBE2G2 suggest that RNF145 is the key regulator acting in a lipid-dependent fashion on ADIPOR2 as the executive branch. In fact, the authors present evidence that RNF145 and ADIPOR2 interact in a lipid-dependent fashion and that RNF145 undergoes rapid auto-ubiquitylation and degradation in overly saturated membrane environments. The authors investigate the role of RNF145 and ADIPOR2 on cell viability, the de novo lipid biosynthesis, and membrane fluidity of in the endoplasmic reticulum. The data are consistent with a key role of both RNF145 and ADIPOR2 on the cellular lipid composition in metabolically stressed and even in unstressed cells.

This study is a very important, extremely well executed study. The manuscript is fun to read , follows a clear logic, and tackles a challenging question with simple experiments performed at highest technical standards. The finding that RNF145 is a key regulator of lipid saturation in mammalian cells is important for the broad readership of EMBO J. I am convinced that this study will have long-lasting impact on the wider fields of membrane biochemistry, lipid metabolism, and membrane cell biology. Even though this manuscript provides no detailed information on the molecular mechanism by which membrane aberrancies are being sensed (which would clearly be beyond the scope of this manuscript), I strongly recommend this manuscript for publication in

EMBO J after minor corrections.

MINOR POINTS:

Organization of figures:
I had great joy and pleasure reading this well-written manuscript and I do not want to urge the authors to make excessive changes. Nevertheless, I felt that some of the data presented in supplementary figures were crucial enough to 'deserve' a place in a main figure. Moving between the main manuscript and the supplementary materials was sometimes distracting Especially the important data in Figure S7 and S8B may be better placed in the respective main figure.

Data availability:
The proteomic data are intriguing. In fact, they are likely to contain other important findings for follow-up research. it would be extremely helpful for the community of all the all relevant data (fold change and p-value) for all data points represented in Figure 1A, 1B, S1A, and S1B could be found in a supplementary tab-delimited data sheet.

Reference to previous work in S. cerevisiae:
Given that several components of the ubiquitin proteasome pathway and the ERAD pathway have been identified based on a lipid saturation sensitive pathway in S. cerevisiae, I would strongly recommend the authors to refer to the pioneering works by Stefan Jentsch and colleagues on the OLE pathway (PMID: 11007476; PMID: 11733065).
Furthermore, my own laboratory has uncovered the molecular basis for lipid saturation sensing (PMID: 27320200; PMID: 32029718), which may be highly relevant for RNF145/ADIPOR2 system. We also studied the role of ERAD pathway via Ubx2p (homolog of UBXD8) on lipid saturation and lipid bilayer stress (PMID: 23891562) and how precisely lipid bilayer stress (e.g. from lipid oversaturation) triggers the UPR (PMID: 28689662).

Because the authors speculate on the role of membrane fluidity on the RNF145/ADIPOR2 system it is important to discuss their findings in the light of the data on the Mga2 lipid saturation sensor in the ER of S. cerevisiae, which is not regulated by membrane fluidity/viscosity (PMID: 32029718).

I want to finish my review by congratulating the authors for very elegant work and a beautifully written manuscript.
Robert Ernst

EMBO Journal ref no. **EMBOJ-2022-110777**

Please find an updated version of our manuscript:

Regulation of membrane fluidity by RNF145-triggered degradation of the lipid hydrolase ADIPOR2

We are grateful for the suggestions of the reviewers and yourself and following our discussion we have included changes as agreed, with further experiments where possible.

These additions include several experiments (microscopy and biochemistry) suggested by reviewer 2, the transfer of several supplementary figures to the main section, a discussion of the OLE1 yeast pathway in the context of the manuscript and referencing of several contributors. The deposition of data sets from our proteomics will be made available as requested by reviewer 3. Also included in this response letter is additional data which addresses the concerns of reviewer 2.

All changes to the manuscript are highlighted in yellow.

Below are the direct responses to reviewers' points:

Reviewer 1: We are grateful to this reviewer for their helpful comments.

Reviewer 2:

(1) What is the mechanism for RNF145 sensing the FA saturation? Is the transmembrane domain of RNF145 important for this event? The authors should map the exact binding sequences of ADIPOR2 and RNF145.

As the reviewer will know, this suggestion goes far beyond the scope of the current manuscript. We are currently investigating the exact molecular mechanism underlying RNF145's responsiveness to FAs. We are aware that the intrinsic properties of RNF145's 14 predicted transmembrane domains will play a prominent role, but a detailed mechanistic description is compounded by the lack of a high-confidence atomic structure of RNF145. Similarly, mapping the exact binding sites of RNF145 and ADIPOR2 is related to the question of how RNF145 senses FAs and is a non-trivial matter for complex membrane proteins. To address this question requires a minimum 1-2 years work and is therefore outside of the scope of this paper.

However, in an attempt to address these questions within the timeframe of this revision, we have performed a number of experiments using RNF145 and ADIPOR2 deletion mutants to

narrow down the domains necessary for their interaction. We have included the data below (**Fig R1 and Fig R2**). We demonstrate that: 1) a portion of the RNF145 TMD region is involved in binding ADIPOR2 (**Fig R1**) and 2) that the highly divergent N-terminus of ADIPOR2 (amino acids 1-140) is dispensable for the interaction with RNF145 (**Fig R2**).

To investigate whether the transmembrane domain of RNF145 was strictly required for binding to ADIPOR2, we first removed the C- and N-terminal cytoplasmic domains of RNF145 (aa 1-51 and aa 507-663, respectively) (**Fig R1A).** RNF145 was still able to bind ADIPOR2 when deleting RNF145's cytoplasmic N-terminal region (amino acids 1–51 directly preceding the first TMD), while deletion of the cytoplasmic C-terminus (aa 507-663) lead to complete destabilisation of the ligase and prevented further assessment (**Figure R1B (i)**). While removal of TMDs 9-14, together with the entire C-terminus (aa 315-663) led to a stable protein that was unable to interact with ADIPOR2 (**Figure R1B (ii), lane 2**), deletion of aa 390 – 663 (TMDs 11-14 and the cytoplasmic C-terminus) or aa 480-663 maintained binding to ADIPOR2 (**Figure R1B (ii), lanes 3 and 4**). All RNF145 TMD deletion mutants were expressed at lower levels than WT RNF145 and bound a lower molecular weight ADIPOR2 species, likely corresponding to a posttranslational modification of ADIPOR2 (**Figure R1B (i/ii)**).

We next investigated whether the extensive cytoplasmic N-terminal region of ADIPOR2 was necessary for the interaction with RNF145. We generated two ADIPOR2 deletion mutants lacking aa 1-116 (retaining the trafficking motif) and aa 1-140 (the majority of the N-terminal sequence preceding the first TMD) (**Figure R2A**). Both deletion mutants were still able to bind RNF145 (**Figure R2B**). Moreover, full length ADIPOR2 and all the N-terminal deletion mutants (including an additional deletion mutant lacking amino acids 1-81) were strongly stabilised following RNF145 depletion (**Figure R2C**). RNF145 is therefore still able to target these ADIPOR2 mutants for degradation. These findings demonstrate a first step towards a more comprehensive mapping of the exact regions mediating RNF145 and ADIPOR2 interaction.

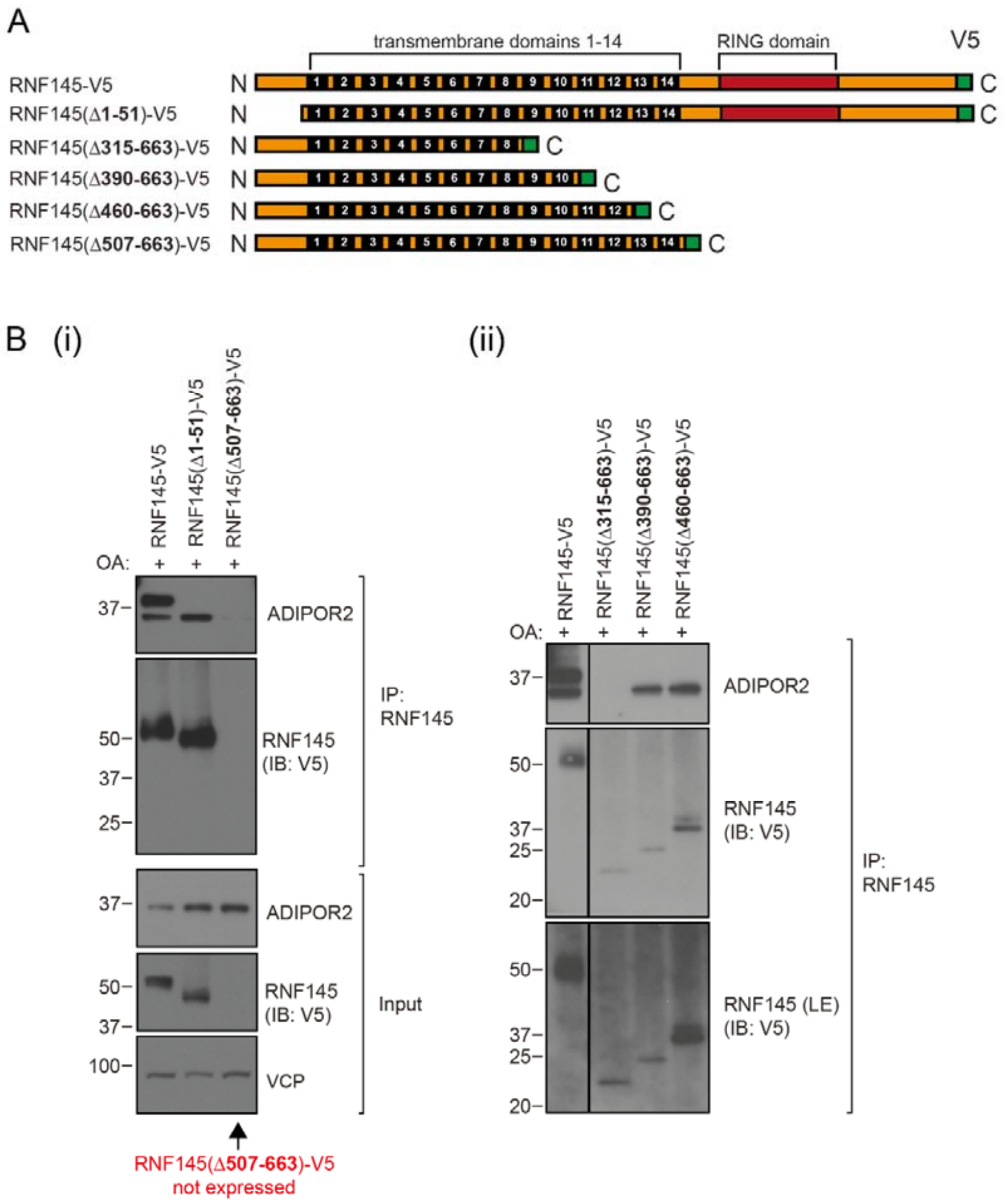

Figure R1. The transmembrane (TMD) region of RNF145 is necessary for ADIPOR2 binding. (A) RNF145 lacking the N-terminus preceding the first TMD interacts with ADIPOR2. RNF145 knockout HEK-293T cells were stably reconstituted with the indicated RNF145 mutants and used for co-immunoprecipitation studies. **(B)** The transmembrane region of RNF145 is involved in ADIPOR2 binding. Cells were treated with OA (200 μM, 3h) and RNF145 was immunoprecipitated (IP) from whole-cell lysate. Deletion mutants were detected by immunoblot analysis.

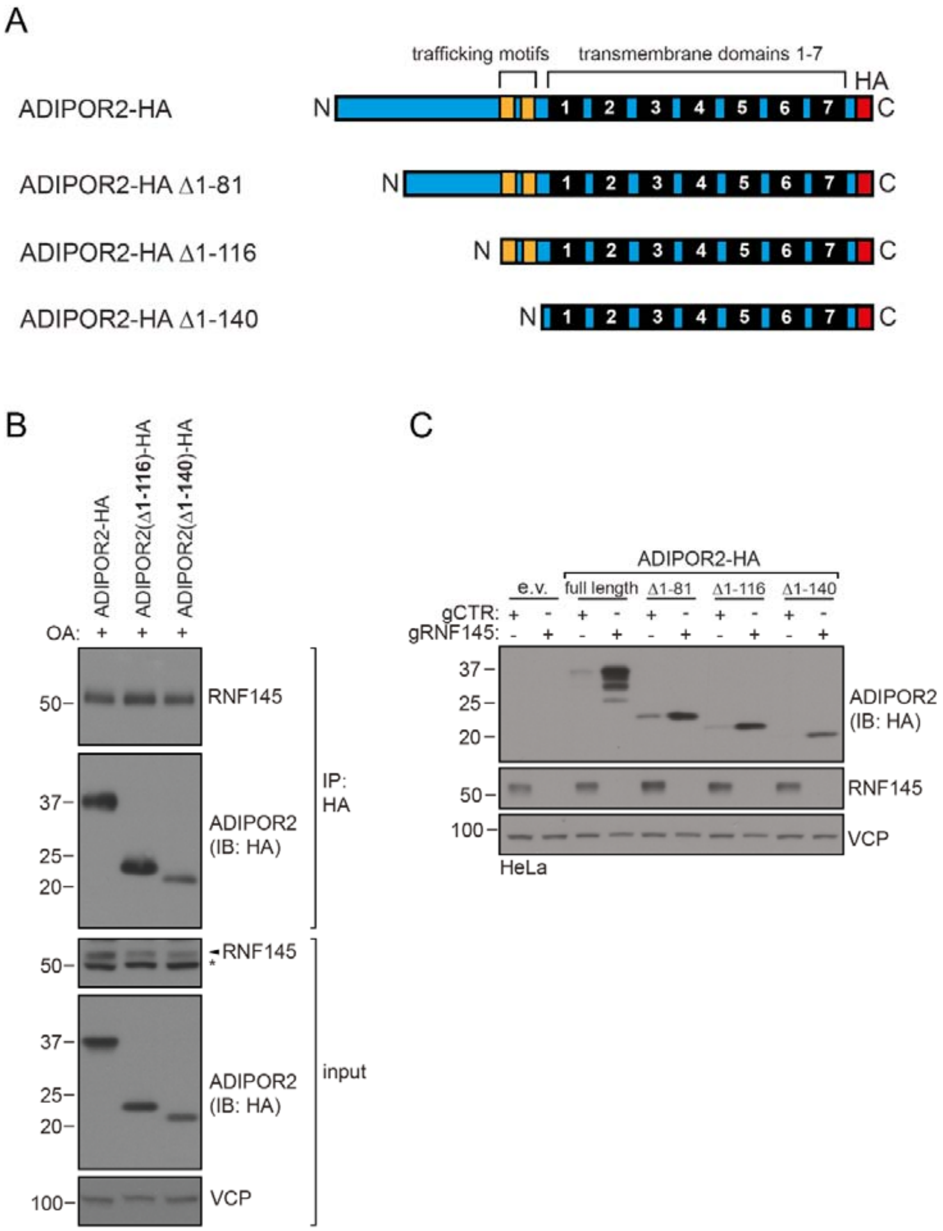

Figure R2. The cytosolic N-terminus of ADIPOR2 is dispensable for the interaction with and regulation by RNF145. (A) Schematic of ADIPOR2 deletion mutants used in (B) and (C). **(B)** N-terminal ADIPOR2 deletion mutants retain interaction with RNF145. HEK-293T cells stably expressing the indicated ADIPOR2 variants were treated with OA (200 μM, 3h) and ADIPOR2 was immunoprecipitated (IP) from whole-cell lysate using an anti-HA antibody. Indicated proteins were detected by immunoblot analysis. **(C)** ADIPOR2 N-terminal deletion mutants retain regulation by RNF145. HeLa cells stably expressing the indicated ADIPOR2 deletion constructs or containing an empty vector control (e.v.) were transiently depleted of RNF145 (gRNF145) or B2M (gCTR). ADIPOR2 levels were detected by immunoblot analysis.

(2) TRC8 was not sensitive to FAs according to the authors' report. The difference between RNF145 and TRC8 may be helpful to address this question [of the role of RNF145's transmembrane domain in FA sensing]

It is correct that levels of Trc8 did not change upon addition of PA or OA as shown in the unbiased approach in **Fig 1A/B** and directly in **Fig EV1C**. The idea of using Trc8 to assess the role of RNF145 is indeed a good one. However, this becomes technically challenging as RNF145 and TRC8 are related but still share only 27% sequence identity. A large proportion of homology is restricted to the C-terminal RING domain, which, as we show in **Fig R1 B(ii),** is not required for RNF145's interaction with ADIPOR2. This relative lack of sequence identity and the paucity of structural data on either RNF145 or Trc8 hinders a rigorous sequence- and/or structure-based definition of which regions of the protein are most likely to play a role in the regulation of RNF145. We suggest that the experiments delineated in (1) (see above) are sufficient to address this question within the remit of this manuscript.

(3) Is the FA compositions in the membrane sensed by RNF145, UBXD8 or other proteins? Can UBXD8 regulate the level of ADIPOR2 under different FAs stimulation*?*

We show in this manuscript that RNF145 is absolutely essential for adjusting ADIPOR2 levels in response to PA/OA (**Fig 4A**), while deleting ADIPOR2 did not prevent FA-mediated regulation of RNF145 (**Fig EV3C**). We also observed that both ADIPOR2 and RNF145 levels are modulated by the E2 enzyme UBE2G2 (which is required for efficient RNF145 turnover as well as RNF145-mediated degradation of ADIPOR2), and components of the VCP complex (e.g. UBXD8) (**Fig 3B, EV2E, EV3B/D**).

We cannot categorically rule out that additional proteins might be involved in the sensing of membrane FA composition by RNF145 and/or ADIPOR2, but the search for additional factors – if indeed there are any – would require an elaborate set of genetic screens and is beyond the scope of this manuscript.

We agree that UBXD8 is an important cofactor of the VCP complex and necessary for the efficient extraction and degradation of ADIPOR2/RNF145 in response to FAs. A report in the literature shows that UBXD8 can respond to OA, which releases UBXD8 from Insig-1 and thereby prevents Insig-1 degradation (Lee et al, 2008, PMID: 18835813).

In contrast, our observation confirms that ADIPOR2 is efficiently targeted for degradation under OA (and not stabilised as is the case for Insig-1). Therefore, the reported model of UBXD8-mediated regulation of Insig-1 does not directly apply to ADIPOR2 and we conclude that UBXD8 is very unlikely to be the FA sensor triggering OA-mediated ADIPOR2 degradation.

However, to experimentally determine whether the OA-sensing ability of UBXD8 plays a role in OA-triggered ADIPOR2 degradation, we examined the effects of a reported OA-insensitive UBXD8 mutant (Kim et al, 2013, PMID: 23720822) on RNF145's ability to bind and degrade ADIPOR2 in response to elevated OA levels. The results of this experiment have been added to the manuscript (**Figure EV3B**) and are discussed in detail in **lines 181 – 191 of the manuscript**:

`[…] UBXD8, which links ubiquitinated proteins with the VCP complex, is required for the degradation of RNF145 and ADIPOR2 (**Fig 2E**). Unsaturated FAs are known to reduce binding between UBXD8 and Insig-1 and leading to reduced Insig1 extraction from the ER membrane and consequently its stabilisation (Lee *et al*, 2008; Kim *et al*, 2013; Lee *et al*, 2010). To test whether a similar UBXD8-mediated mechanism might operate in the stabilisation and accumulation of RNF145 in response to OA, we complemented UBXD8-depleted cells with either WT UBXD8 or an FA-insensitive UBXD8 mutant (Kim *et al*, 2013) and challenged them with OA. While reintroduction of the FA-insensitive UBXD8 variant lead to a global decrease in RNF145 levels, it could not prevent OA-mediated RNF145 stabilisation (**Fig EV3B**). These findings suggest that UBXD8 does not directly regulate the OA-induced engagement of ADIPOR2 by RNF145.'

Our data clearly show that the OA-insensitive mutant did not affect the response of either RNF145 or ADIPOR2 to OA or PA treatment (**Fig EV3B**). Of note, RNF145 was constitutively increased upon UBXD8 depletion and restored upon re-expression of UBXD8 (**Fig EV3B**). This is consistent with UBXD8's prominent role as an accessory factor of the VCP protein extraction complex.

In summary, our data strongly suggest that OA-sensing by UBXD8 does not play a significant role in the sensing of FA composition by RNF145.

(4) Is FA directly sensed or the FA in phospholipids sensed?

In the absence of an *in vitro* reconstitution system and due to the technical difficulties and requirement of removing all the enzymes involved in the fatty acid incorporation pathway (6 fatty acid CoA ligases), this is beyond the scope of the paper.

After entering the plasma membrane exogenous fatty acids rapidly (minutes to hours) accumulate in phospholipids to remarkably high levels (Bruce et al, 1996, PMID: 8670161), making it difficult to differentiate the effects of free fatty acids from phospholipids on RNF145 or ADIPOR2. Experimental tools to inhibit the incorporation of free FAs into phospholipids are blunt (i.e., long fatty acyl CoA synthetase inhibitors such as triacsin C) and we are not

aware of an effective way to completely block their incorporation into phospholipids in live cells.

The question can only be adequately addressed using an *in vitro* reconstitution system of RNF145, ADIPOR2 and the requisite ubiquitination machinery. This is not a trivial undertaking, considering that both RNF145 and ADIPOR2 are polytopic transmembrane domains and would have to be both reconstituted into proteoliposomes with defined lipid content. Furthermore, controlled adjustment of the proteoliposome lipid composition is technically challenging and would require a major effort.

(5) The authors should map the exact ubiquitination site of ADIPOR2. There are 11 lysines in the ADIPOR2 protein, of which 2 are localised within the transmembrane region and 1 faces the ER lumen. Therefore, we mutated all lysines that feature as 'mutant K>R all' in the manuscript Figure 4D. Further attempts to mutate lysines in the cytoplasmic region of ADIPOR2 in an attempt to understand whether a key lysine regulates ADIPOR2 degradation gave mixed results and suggested that there is no single key lysine residue which regulates the turnover of ADIPOR2 – an observation seen with other ubiquitination substrates destined for degradation (Mattiroli & Sixma, 2014, PMID: 24699079). This also suggests that RNF145 may target ADIPOR2 in a relatively promiscuous fashion and does not need a specific lysine for RNF145 mediated turnover. This 'promiscuity' is commonly observed with other E3 ubiquitin ligases (Mattiroli & Sixma, 2014, PMID: 24699079).

(6) The ADIPOR1 and ADIPOR2 proteins are plasma membrane proteins. In figure S2C, the authors show that ADIPOR2 accumulated in the ER in RNF145 depleted cells. The image quality is poor, please show high quality images. As RNF145 is an ER protein, the authors should show the colocalisation between RNF145 and ADIPOR2 with high quality image.

We agree that this is an important point, in particular to determine the subcellular localisation of ADIPOR2. Detailed work by Jon Whitehead's group shows that, in comparison to ADIPOR1-HA, very little, if any, ADIPOR2 traffics to the cell surface in HEK cells overexpressing HA tagged ADIPOR2. SIM super resolution of CHO cells expressing the same ADIPOR1-HA and ADIPOR2-HA constructs confirmed that the majority of ADIPOR2 resides in the ER as opposed to ADIPOR1 which was more abundant in the plasma membrane (Keshvari et al, 2013, PMID: 23376713, Keshvari et al, 2015, PMID: 25892445).

To further confirm the localisation of ADIPOR2 and RNF145, we have included new imaging data showing that both proteins are (1) restricted to the ER and nuclear envelope and (2) co-localise with one another.

Although good antibodies exist for detection of RNF145 and ADIPOR2 by western blot and for protein biochemistry these reagents did not work for microscopy (ICC/IF) and gave diffuse background staining. Therefore, we expressed epitope-tagged versions of RNF145 and ADIPOR2 and now provide detailed images of the localisation of ADIPOR2 and RNF145 and their distribution throughout the ER in **Figure 1C**.

The new data is now described in **lines 118 – 122** of the manuscript:

`[…] The majority of S-tagged ADIPOR2 co-localised with an ER-restricted mEmerald marker (SP–KDEL-Em) (**Fig 1C (i)**) or with HA-tagged RNF145 which also localised in the ER (**Fig 1C (ii)**), but not with the plasma membrane marker farnesylated EGFP (**Fig EV1H**). Thus, in HEK-293T cells, ADIPOR2 is a predominantly ER-localised membrane protein that co-localises with RNF145.'

Cells expressing ADIPOR2-S and Farnesylated-mEmerald (mEmerald-Farnsyl-5 RRID: Addgene_54093), which inserts into the inner leaflet of the plasma membrane, show very little, if any, signal overlap (co-localisation) (**Fig EV1H**). These data demonstrate that, under the conditions tested, very little, if any ADIPOR2 resides at the plasma membrane.

To show colocalisation of RNF145 and ADIPOR2 we transiently depleted cells of UBE2G2 – a prerequisite to stabilise ADIPOR2-S to levels sufficient for visualisation in the presence of RNF145-HAx3. Both proteins show extensive co-localisation and a reticular distribution corresponding to the ER (**Figure 1C(ii)**).

(7) I noticed that the RNF145 transcript level decreased much in PA treatment (Figure S5). As RNF145 transcription is regulated by LXR, it seems that the PA treatment inhibited LXR activity. LXR also regulates fatty acid synthesis by regulating SREBP-1c and FASN expression; I think LXR may be important in the model reported here. So I suggest the authors try LXR agonist to activates RNF145 transcription, and check the ADIPOR2 protein level and the membrane fluidity.

We have carefully considered the involvement of LXR in the PA-dependent transcriptional regulation of RNF145. However, our current data do not support this hypothesis for the following reasons:

(a) ectopically expressed RNF145 (lacking any LXR binding sites in the promoter region) still responds to PA, implying PA's inhibition of LXRs does not play a notable role in the fatty-acid mediated regulation of RNF145 levels.

(b) In a previous report (Menzies et al, 2018, PMID 30543180) we did not observe a significant effect of the LXR agonist GW3965 on RNF145 transcript and protein levels (Fig 5 Supplement 2 therein).

(c) We see multiple caveats with the suggested experiment: PA would likely be in significant excess to GW3965 and we have observed pleotropic effects of GW3965 on RNF145 at higher concentrations of the LXR agonist. Moreover, the comparatively short time frame of treatment with PA cannot be compared to that of our previous experiment or other experiments such as those reported in Cook et al, 2017, PMID 28231341. The implication from these caveats is that it is more likely that if PA antagonises the LXR signalling axis, it is unclear how we could generate a series of experiments in which a readout would give the reviewer's suggested response.

(8) Is insig required for the RNF145-mediated degradation of ADIPOR?

This is a good question as the INSIG proteins play a role in RNF145-mediated degradation of HMG-CoA reductase. We addressed this by measuring OA-mediated ADIPOR2 turnover in INSIG-1/2 double knockout cells. The new data (**see Fig EV3E**) show that INSIGs do not play a role in the RNF145 mediated degradation of ADIPOR2. In particular, **Fig EV3E (i)** demonstrates that in cells lacking INSIG1 and 2, ADIPOR2 levels are still regulated in response to PA/OA.

The new data is now described in **lines 213 – 217 of the manuscript** as follows:

'[…] The insulin induced genes 1 and 2 (INSIG1/2) are intermediaries, allowing RNF145 to interact with its substrate 3-hydroxy-3-methylglutaryl-coenzyme A reductase (HMGCR) (Menzies *et al*, 2018; Jiang *et al*, 2018). We therefore tested whether the INSIG proteins were required for the RNF145-dependent regulation of ADIPOR2 and show that a double knockout of INSIG1/2, did not affect RNF145-mediated regulation of ADIPOR2 (**Fig EV3E**).
'

And in lines **375 – 407**:

HMG-CoA reductase (HMGCR) controls the rate-limiting step of the cholesterol *de novo* biosynthesis pathway. In excess sterols, RNF145 and gp78 are recruited to, ubiquitinate and promote the degradation of HMGCR, via the INSIG proteins. The involvement of RNF145 in regulating both HMGCR and ADIPOR2 might reflect a close co-ordination between the regulation of FAs and sterols. Our current data suggest that although RNF145 uses its

transmembrane region to interact with ADIPOR2, INSIGs are not required for ADIPOR2 regulation (**Fig EV3E**). RNF145 may therefore integrate sterol and FA information differently. It is interesting to note that the 'INSIG-dependent' regulation of HMGCR is shared with another E3 ligase, gp78, which can compensate for the loss of RNF145. Conversely, gp78 does not play a major role in ADIPOR2 regulation and RNF145 seems the only E3 ligase sufficient and necessary for ADIPOR2 turnover in the cell lines tested.

Under conditions of excess sterols, INSIG proteins are essential for the recruitment of RNF145 and gp78 to their substrate HMG-CoA reductase (HMGCR) which controls the rate-limiting step of the cholesterol *de novo* biosynthesis pathway.

Involvement of RNF145 in regulating both HMGCR and ADIPOR2 might reflect that the regulation of FAs and sterols are closely coordinated. However, our current data suggest that although RNF145 needs its transmembrane region for interaction with ADIPOR2, INSIGs are not required for ADIPOR2 regulation (**Fig EV3F**). This is exciting as it suggests that RNF145 does indeed integrate sterol and FA information differently. It is interesting to note that the 'INSIG-dependent' regulation of HMGCR is shared with another E3 ligase, gp78, which can compensate for the loss of RNF145. Conversely, gp78 does not play a major role in ADIPOR2 regulation (also see **Fig EV1D**) and RNF145 seems the only E3 ligase sufficient and necessary for ADIPOR2 turnover in the cell lines tested.

Sensing changes in membrane lipid composition provides a substantial challenge as lipids, the substrates to be regulated, are themselves embedded within the membrane bilayer. Integral membrane proteins are ideally suited to constantly monitor physicochemical membrane properties and communicate with downstream effectors via a conformational relay system. RNF145 appears particularly sensitive to FA saturation rather than chain length and likely responds to global changes in membrane properties (i.e. membrane fluidity/viscosity and thickness) instead of individual lipid species. Whether and exactly how RNF145 can directly sense membrane properties through its extensive transmembrane domains is an exciting avenue of future research.

REVIEWER #3

We are grateful to this reviewer for their helpful and encouraging comments.

(9) I felt that some of the data presented in supplementary figures were crucial enough to 'deserve' a place in a main figure. Moving between the main manuscript and the supplementary materials was sometimes distracting Especially the important data in Figure S7 and S8B may be better placed in the respective main figure.

We thank the reviewer for these suggestions and have now moved these supplementary data into the main figures with several other changes that were needed to comply to EMBO J formatting. Former Data from 'former Supplementary Figure S8B' has been moved to the main section and can now be found in **Fig 5B**. We also moved Supplementary Figure 9 to Fig 5 as **Fig 5D**. Data of the previous Supplementary Figure S7 can now be found in **Fig EV3D** (we decided against moving it to the main figure so as to maintain the flow of Fig 4).

(10) The proteomic data are intriguing. In fact, they are likely to contain other important findings for follow-up research. it would be extremely helpful for the community of all the all relevant data (fold change and p-value) for all data points represented in Figure 1A, 1B, S1A, and S1B could be found in a supplementary tab-delimited data sheet.

We now provide additional excel spreadsheets in the Auxiliary data for Figure 1A, 1B, S1A, and S1B and these can be found in **Aux Fig 1**, along with spreadsheets for the lipidomics (**Aux Fig. 5**) all of the proteomics data is uploaded to the PRIDE database

Project accession: REDACTED

Reviewer account details:

Username: REDACTED

Password: REDACTED

Reference to previous work in S. cerevisiae:

(11) Given that several components of the ubiquitin proteasome pathway and the ERAD pathway have been identified based on a lipid saturation sensitive pathway in S. cerevisiae, I would strongly recommend the authors to refer to the pioneering works by Stefan Jentsch and colleagues on the OLE pathway (PMID: 11007476; PMID: 11733065). Furthermore, my own laboratory has uncovered the molecular basis for lipid saturation sensing (PMID: 27320200; PMID: 32029718), which may be highly relevant for RNF145/ADIPOR2 system. We also studied the role of ERAD pathway via Ubx2p (homolog of UBXD8) on lipid saturation and lipid bilayer stress (PMID: 23891562) and how precisely lipid bilayer stress (e.g. from lipid oversaturation) triggers the UPR (PMID: 28689662).
Because the authors speculate on the role of membrane fluidity on the RNF145/ADIPOR2 system it is important to discuss their findings in the light of the data on the Mga2 lipid saturation sensor in the ER of S. cerevisiae, which is not regulated by membrane fluidity/viscosity (PMID: 32029718).

We apologise for the oversight in referencing this important information.

We have now included the following references:

(PMID: 11007476 Hoppe et al., 2000) at lines 346/347

(PMID: 11733065 Rape et al., 2001) at lines 346/347

(PMID: 27320200 Covino et al 2016) at lines 343

(PMID: 32029718 Ballweg et al., 2020) at lines 342, 346/347

(PMID: 23891562Surma et al., 2013) at lines 346/347

(PMID: 23891562 Halbleib et al., 2017) at lines 340

We also add discussions on the yeast OLE1/VCP system to our discussion as follows (**lines 331-373**):

Sensing changes in membrane lipid composition provides a substantial challenge as lipids, the substrates to be regulated, are themselves embedded within the membrane bilayer. Integral membrane proteins are ideally suited to constantly monitor physicochemical membrane properties and communicate with downstream effectors via a conformational relay system. RNF145 appears particularly sensitive to FA saturation rather than chain length and may respond to global changes in membrane properties (i.e. membrane fluidity/viscosity and thickness) instead of individual lipid species. Exactly how RNF145 can directly sense membrane properties through its extensive transmembrane domains is an exciting avenue of future research.

The role of ERAD proteins in lipid homeostasis is best understood in yeast, where the ER-resident, transcription factors Mga2 and Spt23 regulate expression of the yeast acyl-CoA desaturase (*OLE1*) to enable fatty acid desaturation (Covino *et al*, 2016; Ballweg *et al*, 2020). Homodimeric, membrane-bound Mga2 and Spt23 are proteolytically activated in an FA-dependent manner by the combined action of the cytosolic E3 ligase Rsp5 and yeast ERAD machinery (Rape *et al*, 2001; Surma *et al*, 2013; Ballweg *et al*, 2020; Hoppe *et al*, 2000; Ballweg & Ernst, 2017).

Mga2 senses the lipid-packing density in the membrane, rather than membrane fluidity itself. Although mammals lack orthologues of the yeast Mga2 and Spt23 membrane sensors, the increased complexity of membrane lipid composition suggest that mammalian cells are likely to have evolved additional analogous lipid sense-and-response modules. These would register distinct lipid species and membrane parameters to initiate an integrated cellular response. The sensing modalities are likely to differ, depending on which membrane properties are being monitored. Nevertheless, they seem to utilize a shared subset of ERAD components. We observed the involvement of VCP and UBXD8 in the FA-dependent (and

steady-state) degradation of RNF145/ADIPOR2, similar to the role of Ubx2 in Mga2/Spt23 processing.

Like Mga2, the regulated turnover of ADIPOR2 by RNF145 is contingent on a lipid environment that changes steady state membrane properties. It will be interesting to determine how RNF145 senses changes in membrane lipid properties. The RNF145/ADIPOR2 sense-and-response module seems distinct from that of Rsp5/Mga2 since RNF145 is itself an integral membrane protein and its auto-ubiquitination and turnover have an important role in the modulation of ADIPOR2.

Reminiscent of the OLE1 transcription pathway, but in response to cholesterol, the ER-resident SREBP cleavage-activating chaperone (SCAP), in complex with the SREBP2 transcription factor, couples membrane cholesterol sensing to cellular cholesterol demand. SCAP senses excess sterols *via* a loop in its sterol-sensing domain (SSD). Sterol binding induces a conformation which sequesters the SCAP-SREBP2 complex in an inactive state with INSIG1/2 and thereby suppresses transcription of SREBP2 target genes (Brown *et al*, 2002; Radhakrishnan *et al*, 2004; Brown *et al*, 2018).

1st Revision - Editorial Decision 12th Jul 2022

Thank you for submitting your revised manuscript to The EMBO Journal. Both referees 2 and 3 have now re-reviewed it, and are, as you will see from their comments below, fully satisfied with the revisions. We shall be happy to accept the manuscript for publication, as soon as a few remaining editorial points listed below have been addressed:

Referee #2:

The authors have done their best to address my questions. I appreciate their effects and the manuscript has been dramatically improved. The data quality is high. I have no further question.

Referee #3:

The conclusions of this study are of general interest by identifying and dissecting a pathway that control lipid saturation in mammalian cells. The findings of this study are highly relevant for conditions of lipotoxicity, ER-stress, and lipid bilayer stress.

The authors have carefully and impressively addressed the concerns of all three reviewers. The points raised by reviewer 2 and 3 were very reasonably addressed.

I must state: This manuscript is among the best manuscripts I read (and will read) this year. I strongly recommend the publication of this study and want to congratulate the authors for excellent work.

EMBO Press Author Checklist

Corresponding Author Name: Professor Paul J Lehner	
Journal Submitted to: EMBO Journal	
Manuscript Number: EMBOJ-2022-110777	

USEFUL LINKS FOR COMPLETING THIS FORM
The EMBO Journal - Author Guidelines
EMBO Reports - Author Guidelines
Molecular Systems Biology - Author Guidelines
EMBO Molecular Medicine - Author Guidelines

Reporting Checklist for Life Science Articles (updated January 2022)
This checklist is adapted from Materials Design Analysis Reporting (MDAR) Checklist for Authors. MDAR establishes a minimum set of requirements in transparent reporting in the life sciences (see Statement of Task: 10.31222/osf.io/9sm4x). Please follow the journal's guidelines in preparing your manuscript.
Please note that a copy of this checklist will be published alongside your article.

Abridged guidelines for figures
1. Data
The data shown in figures should satisfy the following conditions:
- the data were obtained and processed according to the field's best practice and are presented to reflect the results of the experiments in an accurate and unbiased manner.
- ideally, figure panels should include only measurements that are directly comparable to each other and obtained with the same assay.
- plots include clearly labeled error bars for independent experiments and sample sizes. Unless justified, error bars should not be shown for technical replicates.
- if n<5, the individual data points from each experiment should be plotted. Any statistical test employed should be justified.
- Source Data should be included to report the data underlying figures according to the guidelines set out in the authorship guidelines on Data Presentation.

2. Captions
Each figure caption should contain the following information, for each panel where they are relevant:
- a specification of the experimental system investigated (eg cell line, species name).
- the assay(s) and method(s) used to carry out the reported observations and measurements.
- an explicit mention of the biological and chemical entity(ies) that are being measured.
- an explicit mention of the biological and chemical entity(ies) that are altered/varied/perturbed in a controlled manner.
- the exact sample size (n) for each experimental group/condition, given as a number, not a range;
- a description of the sample collection allowing the reader to understand whether the samples represent technical or biological replicates (including how many animals, litters, cultures, etc.).
- a statement of how many times the experiment shown was independently replicated in the laboratory.
- definitions of statistical methods and measures:
  - common tests, such as t-test (please specify whether paired vs. unpaired), simple $\chi 2$ tests, Wilcoxon and Mann-Whitney tests, can be unambiguously identified by name only, but more complex techniques should be described in the methods section;
  - are tests one-sided or two-sided?
  - are there adjustments for multiple comparisons?
  - exact statistical test results, e.g., P values = x but not P values < x;
  - definition of 'center values' as median or average;
  - definition of error bars as s.d. or s.e.m.

Please complete ALL of the questions below.
Select "Not Applicable" only when the requested information is not relevant for your study.

Materials

Newly Created Materials	Information included in the manuscript?	In which section is the information available? (Reagents and Tools Table, Materials and Methods, Figures, Data Availability Section)
New materials and reagents need to be available; do any restrictions apply?	Yes	Material and Methods

Antibodies	Information included in the manuscript?	In which section is the information available? (Reagents and Tools Table, Materials and Methods, Figures, Data Availability Section)
For **antibodies** provide the following information:<br>- Commercial antibodies: RRID (if possible) or supplier name, catalogue number and or/clone number<br>- Non-commercial: RRID or citation	Yes	Materials and Methods

DNA and RNA sequences	Information included in the manuscript?	In which section is the information available? (Reagents and Tools Table, Materials and Methods, Figures, Data Availability Section)
**Short novel DNA or RNA including primers, probes**: provide the sequences.	Yes	Material and Methods

Cell materials	Information included in the manuscript?	In which section is the information available? (Reagents and Tools Table, Materials and Methods, Figures, Data Availability Section)
**Cell lines:** Provide species information, strain. Provide accession number in repository **OR** supplier name, catalog number, clone number, and/**OR** RRID.	Yes	Materials and Methods
**Primary cultures:** Provide species, strain, sex of origin, genetic modification status.	Yes	Materials and Methods
Report if the cell lines were recently **authenticated** (e.g., by STR profiling) and tested for mycoplasma contamination.	Yes	Materials and Methods

Experimental animals	Information included in the manuscript?	In which section is the information available? (Reagents and Tools Table, Materials and Methods, Figures, Data Availability Section)
**Laboratory animals or Model organisms:** Provide species, strain, sex, age, genetic modification status. Provide accession number in repository **OR** supplier name, catalog number, clone number, **OR** RRID.	Not Applicable	
**Animal observed in or captured from the field:** Provide species, sex, and age where possible.	Not Applicable	
Please detail **housing and husbandry conditions**.	Not Applicable	

Plants and microbes	Information included in the manuscript?	In which section is the information available? (Reagents and Tools Table, Materials and Methods, Figures, Data Availability Section)
**Plants:** provide species and strain, ecotype and cultivar where relevant, unique accession number if available, and source (including location for collected wild specimens).	Not Applicable	
**Microbes:** provide species and strain, unique accession number if available, and source.	Not Applicable	

Human research participants	Information included in the manuscript?	In which section is the information available? (Reagents and Tools Table, Materials and Methods, Figures, Data Availability Section)
If collected and within the bounds of privacy constraints report on age, sex and gender or ethnicity for all study participants.	Not Applicable	

Core facilities	Information included in the manuscript?	In which section is the information available? (Reagents and Tools Table, Materials and Methods, Figures, Data Availability Section)
If your work benefited from core facilities, was their service mentioned in the acknowledgments section?	Yes	Acknowledgments

Design

Study protocol	Information included in the manuscript?	In which section is the information available? (Reagents and Tools Table, Materials and Methods, Figures, Data Availability Section)
If study protocol has been **pre-registered, provide DOI in the manuscript**. For clinical trials, provide the trial registration number **OR** cite DOI.	Not Applicable	
Report the **clinical trial registration number** (at ClinicalTrials.gov or equivalent), where applicable.	Not Applicable	

Laboratory protocol	Information included in the manuscript?	In which section is the information available? (Reagents and Tools Table, Materials and Methods, Figures, Data Availability Section)
Provide DOI OR other citation details if **external detailed step-by-step protocols** are available.	Not Applicable	

Experimental study design and statistics	Information included in the manuscript?	In which section is the information available? (Reagents and Tools Table, Materials and Methods, Figures, Data Availability Section)
Include a statement about **sample size** estimate even if no statistical methods were used.	Yes	Figure legends and Materials and Methods
Were any steps taken to minimize the effects of subjective bias when allocating animals/samples to treatment (e.g. **randomization procedure**)? If yes, have they been described?	Not Applicable	
Include a statement about **blinding** even if no blinding was done.	Yes	Material and Methods
Describe **inclusion/exclusion criteria** if samples or animals were excluded from the analysis. Were the criteria pre-established?<br><br>If sample or data points were omitted from analysis, report if this was due to attrition or intentional exclusion and provide justification.	Not Applicable	
For every figure, are **statistical tests** justified as appropriate? Do the data meet the assumptions of the tests (e.g., normal distribution)? Describe any methods used to assess it. Is there an estimate of variation within each group of data? Is the variance similar between the groups that are being statistically compared?	Yes	Figure legends and Materials and Methods

Sample definition and in-laboratory replication	Information included in the manuscript?	In which section is the information available? (Reagents and Tools Table, Materials and Methods, Figures, Data Availability Section)
In the figure legends: state number of times the experiment was **replicated** in laboratory.	Yes	Figure legends
In the figure legends: define whether data describe **technical or biological replicates**.	Yes	Figure legends

Ethics

Ethics	Information included in the manuscript?	In which section is the information available? (Reagents and Tools Table, Materials and Methods, Figures, Data Availability Section)
Studies involving **human participants**: State details of **authority granting ethics approval** (IRB or equivalent committee(s), provide reference number for approval.	Not Applicable	
Studies involving **human participants**: Include a statement confirming that **informed consent** was obtained from all subjects and that the experiments conformed to the principles set out in the WMA Declaration of Helsinki and the Department of Health and Human Services Belmont Report.	Not Applicable	
Studies involving **human participants:** For publication of **patient photos**, include a statement confirming that consent to publish was obtained.	Not Applicable	
Studies involving experimental **animals**: State details of **authority granting ethics approva**l (IRB or equivalent committee(s), provide reference number for approval. Include a statement of compliance with ethical regulations.	Not Applicable	
Studies involving **specimen and field samples:** State if relevant **permits** obtained, provide details of authority approving study; if none were required, explain why.	Not Applicable	

Dual Use Research of Concern (DURC)	Information included in the manuscript?	In which section is the information available? (Reagents and Tools Table, Materials and Methods, Figures, Data Availability Section)
Could your study fall under dual use research restrictions? Please check biosecurity documents and list of **select agents and toxins** (CDC): https://www.selectagents.gov/sat/list.htm	Not Applicable	
If you used a select agent, is the security level of the lab appropriate and reported in the manuscript?	Not Applicable	
If a study is subject to dual use research of concern regulations, is the name of the **authority granting approval and reference number** for the regulatory approval provided in the manuscript?	Not Applicable	

Reporting

The MDAR framework recommends adoption of discipline-specific guidelines, established and endorsed through community initiatives. Journals have their own policy about requiring specific guidelines and recommendations to complement MDAR.

Adherence to community standards	Information included in the manuscript?	In which section is the information available? (Reagents and Tools Table, Materials and Methods, Figures, Data Availability Section)
State if relevant guidelines or checklists (e.g., **ICMJE, MIBBI, ARRIVE, PRISMA**) have been followed or provided.	Not Applicable	
For **tumor marker prognostic studies,** we recommend that you follow the **REMARK** reporting guidelines (see link list at top right). See author guidelines, under 'Reporting Guidelines'. Please confirm you have followed these guidelines.	Not Applicable	
For **phase II and III randomized controlled trials**, please refer to the **CONSORT** flow diagram (see link list at top right) and submit the CONSORT checklist (see link list at top right) with your submission. See author guidelines, under 'Reporting Guidelines'. Please confirm you have submitted this list.	Not Applicable	

Data Availability

Data availability	Information included in the manuscript?	In which section is the information available? (Reagents and Tools Table, Materials and Methods, Figures, Data Availability Section)
Have **primary datasets** been deposited according to the journal's guidelines (see 'Data Deposition' section) and the respective accession numbers provided in the Data Availability Section?	Yes	Materials and Methods, Figures and Data availability section
Were **human clinical and genomic datasets** deposited in a public access-controlled repository in accordance to ethical obligations to the patients and to the applicable consent agreement?	Not Applicable	
Are **computational models** that are central and integral to a study available without restrictions in a machine-readable form? Were the relevant accession numbers or links provided?	Not Applicable	
If publicly available data were reused, provide the respective **data citations in the reference list**.	Not Applicable	
